# Supplementary figures and images for: Wnt induces FZD5/8 endocytosis and degradation and the involvement of RSPO-ZNRF3/RNF43 and DVL
Source: eLife. 2025 Oct 10;14:RP103996. doi: 10.7554/eLife.103996 (PMC12513720; doi:10.7554/eLife.103996)

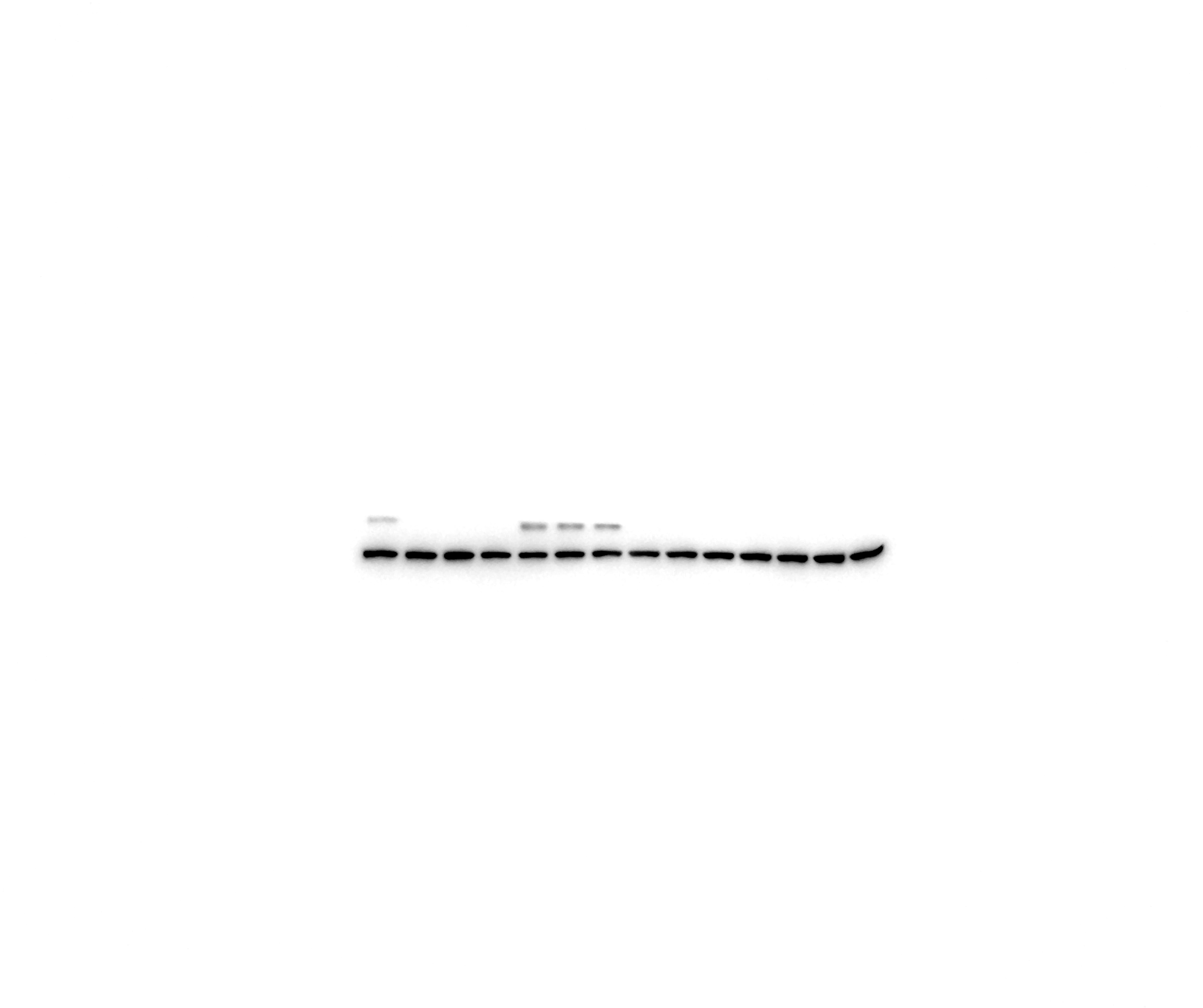

Supplement: Figure 1—source data 1. [file elife-103996-fig1-data1.zip › elife-103996-fig1-data1-v1/Figure 1B/Actin(FZD1-3).tif]

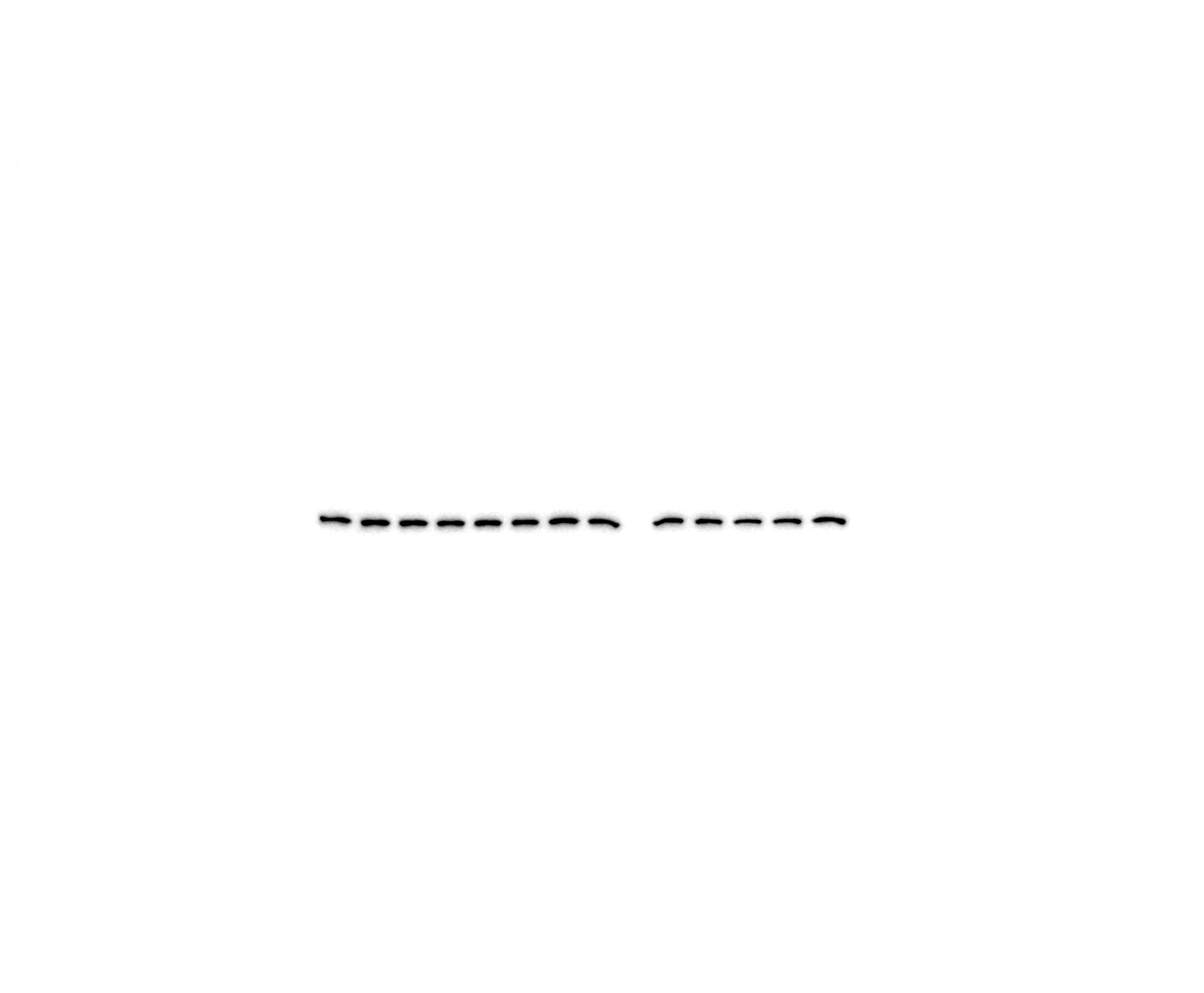

Supplement: Figure 1—source data 1. [file elife-103996-fig1-data1.zip › elife-103996-fig1-data1-v1/Figure 1B/Actin(FZD4 FZD7 FZD8).tif]

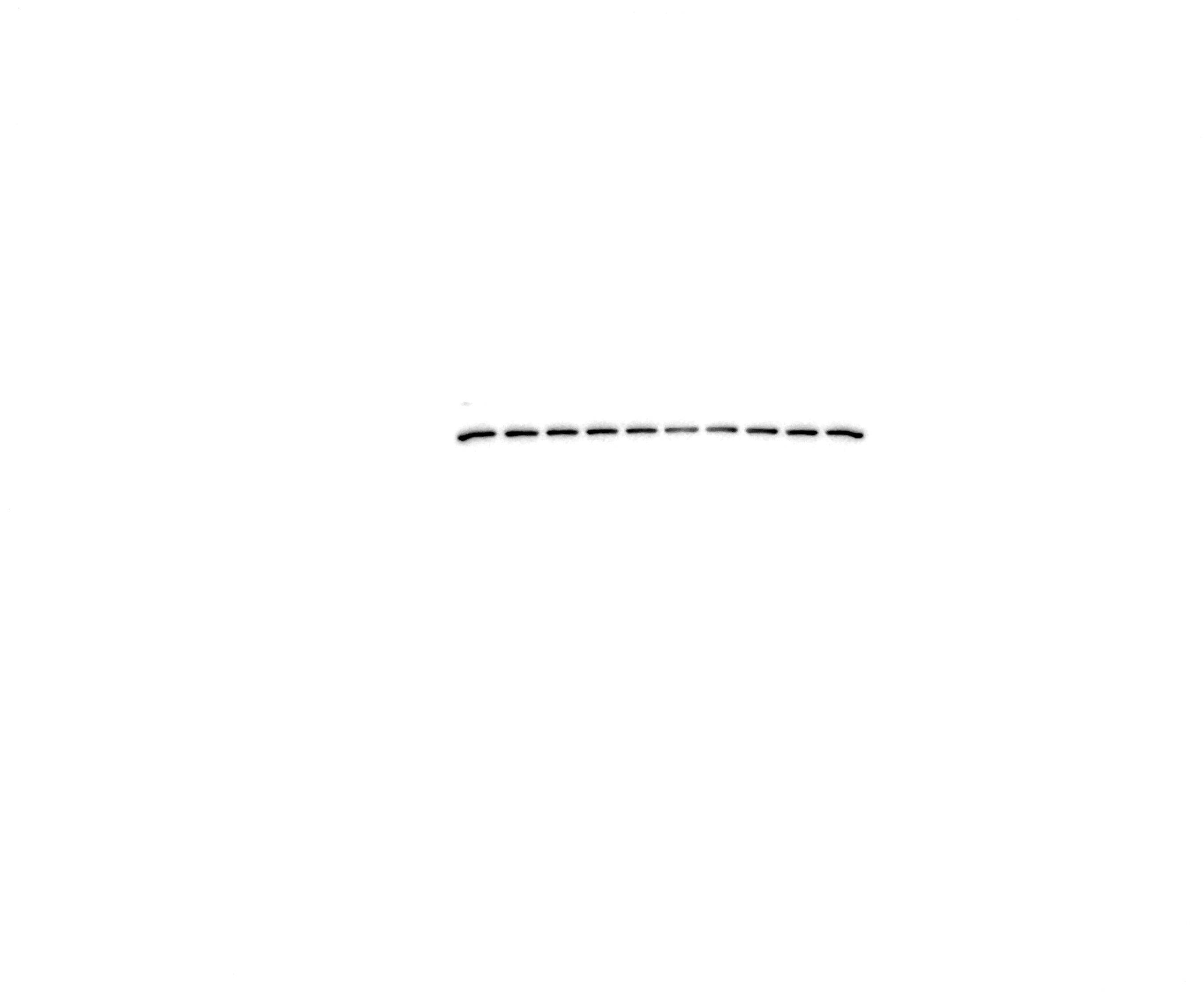

Supplement: Figure 1—source data 1. [file elife-103996-fig1-data1.zip › elife-103996-fig1-data1-v1/Figure 1B/Actin(FZD5-6).tif]

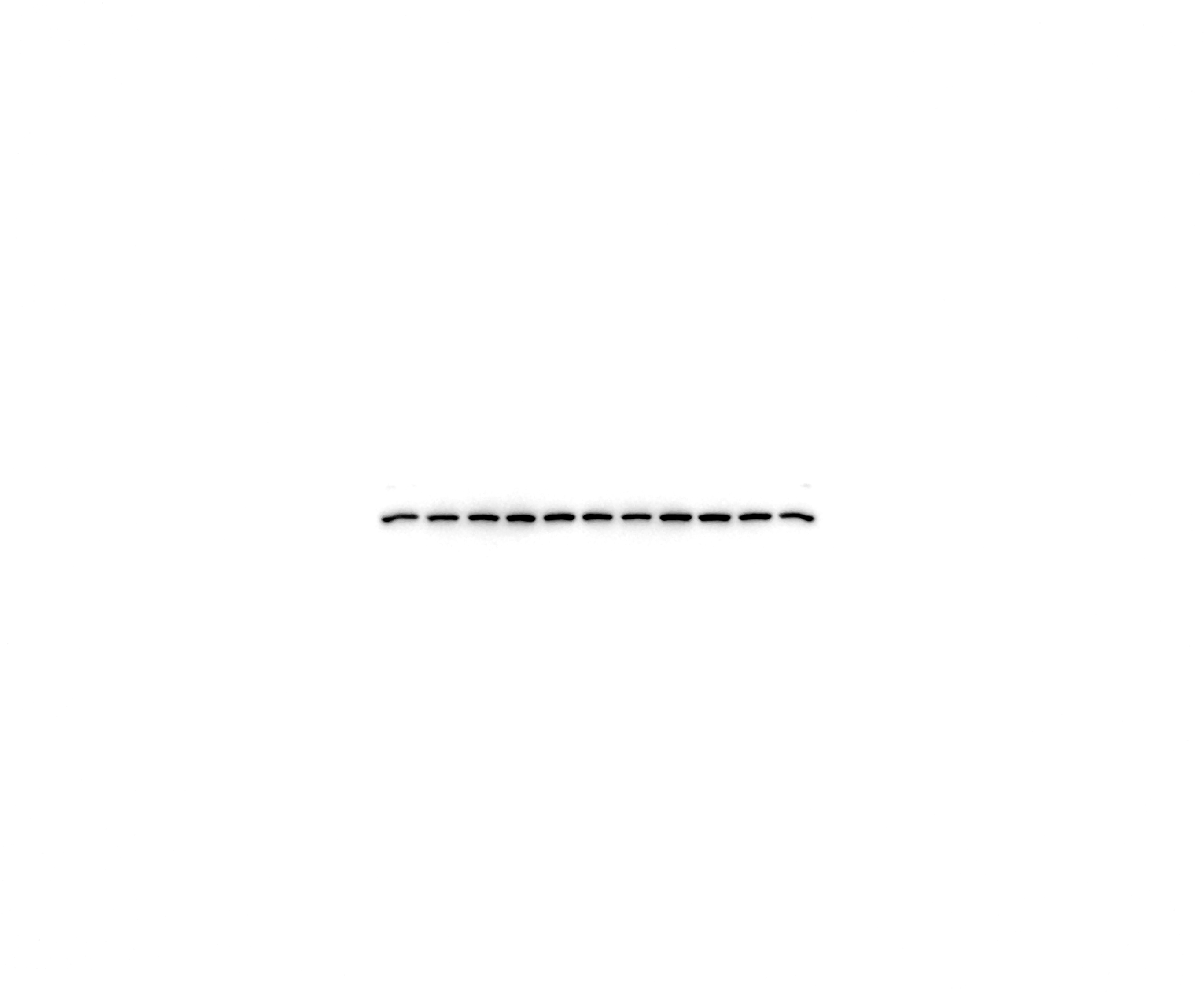

Supplement: Figure 1—source data 1. [file elife-103996-fig1-data1.zip › elife-103996-fig1-data1-v1/Figure 1B/Actin(FZD9-10).tif]

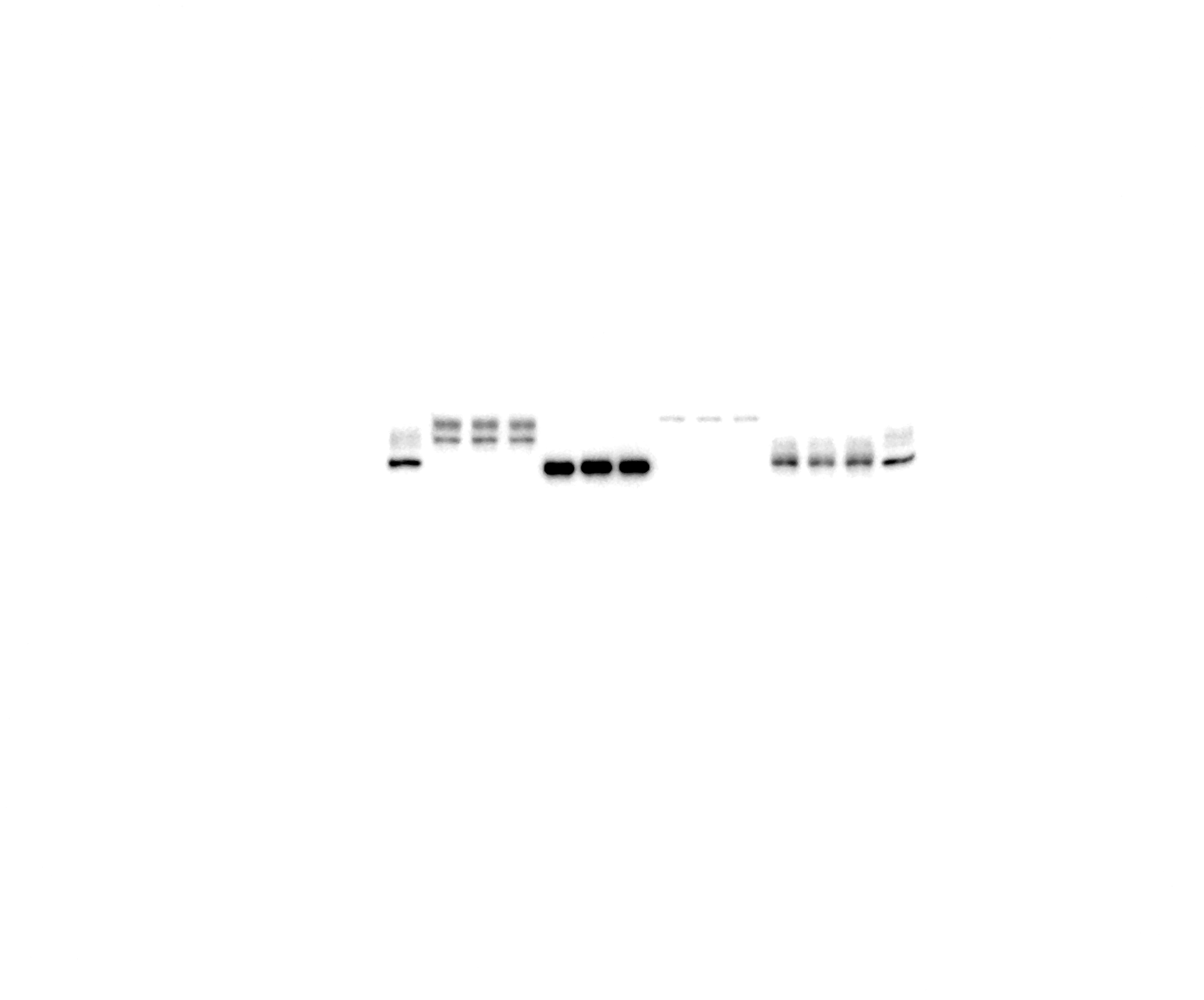

Supplement: Figure 1—source data 1. [file elife-103996-fig1-data1.zip › elife-103996-fig1-data1-v1/Figure 1B/V5(FZD1-3).tif]

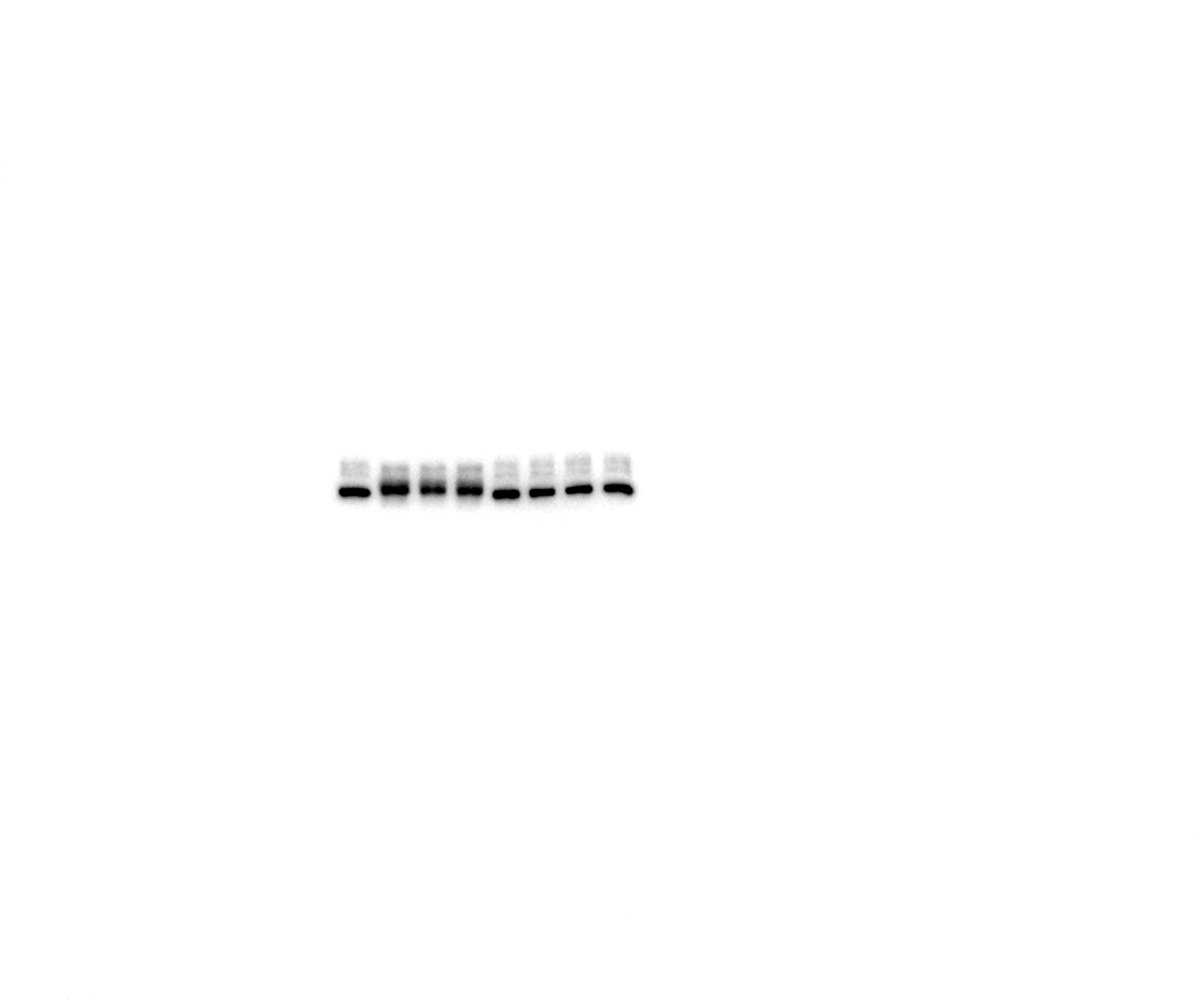

Supplement: Figure 1—source data 1. [file elife-103996-fig1-data1.zip › elife-103996-fig1-data1-v1/Figure 1B/V5(FZD4 FZD7).tif]

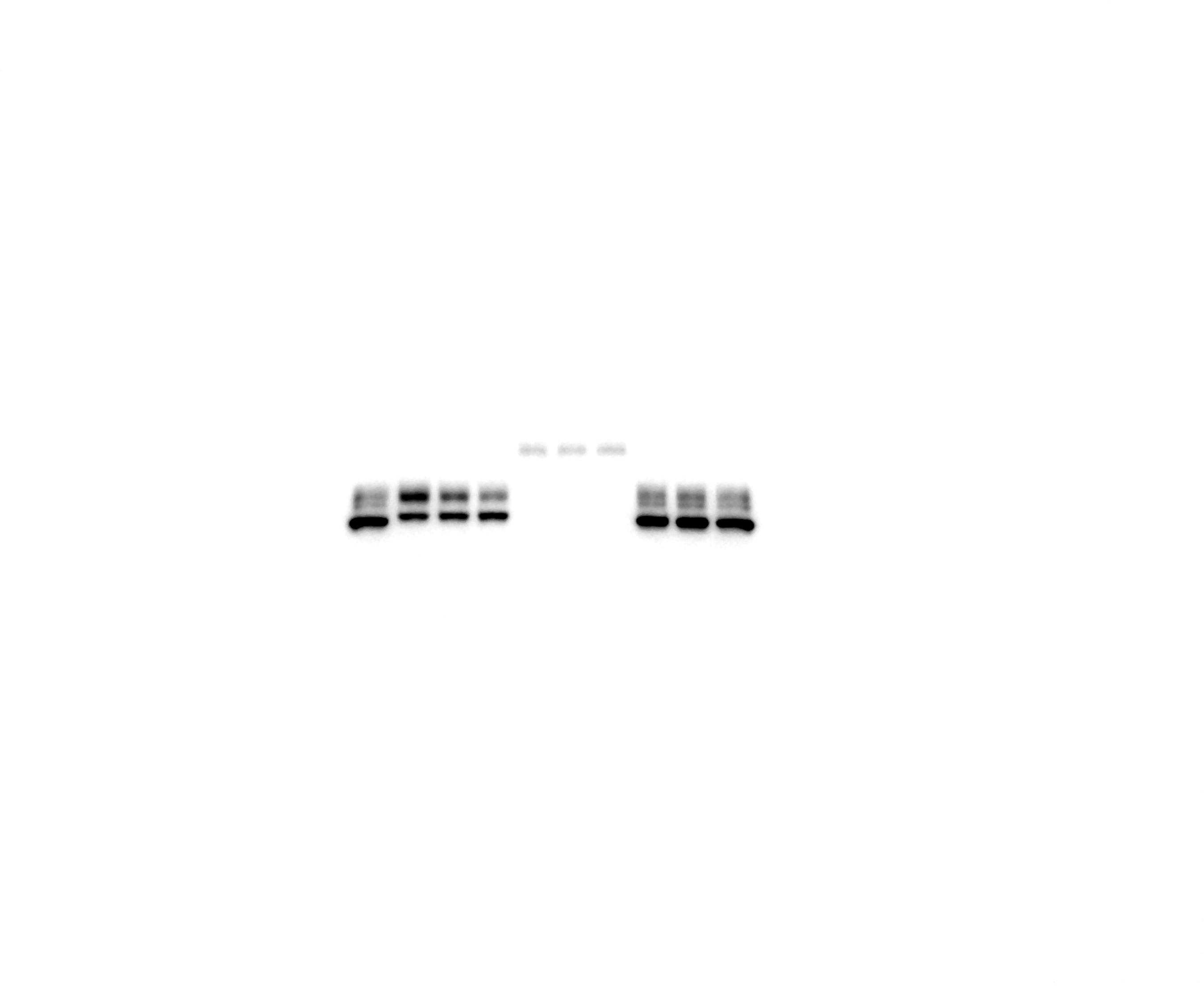

Supplement: Figure 1—source data 1. [file elife-103996-fig1-data1.zip › elife-103996-fig1-data1-v1/Figure 1B/V5(FZD5-6).tif]

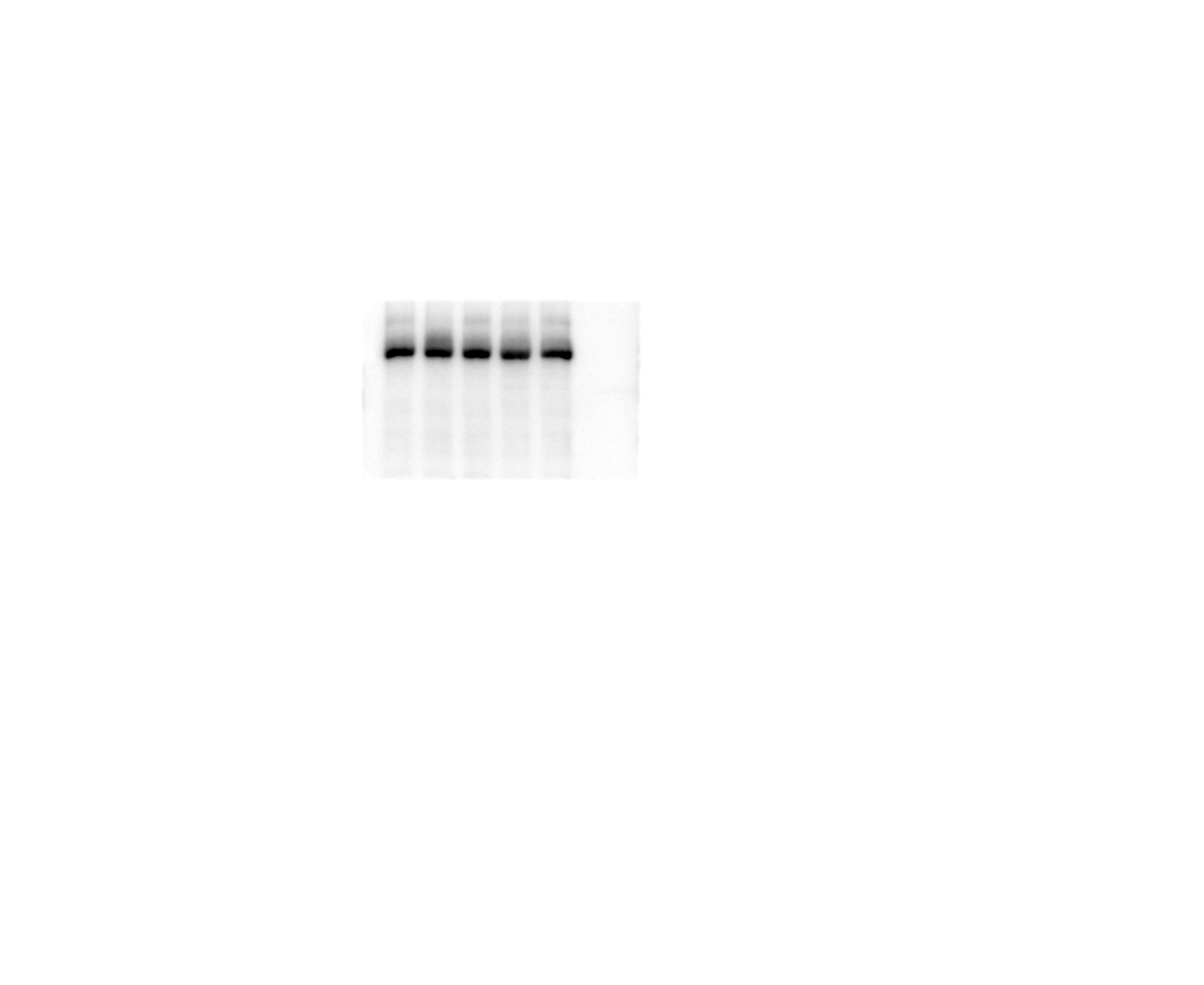

Supplement: Figure 1—source data 1. [file elife-103996-fig1-data1.zip › elife-103996-fig1-data1-v1/Figure 1B/V5(FZD8).tif]

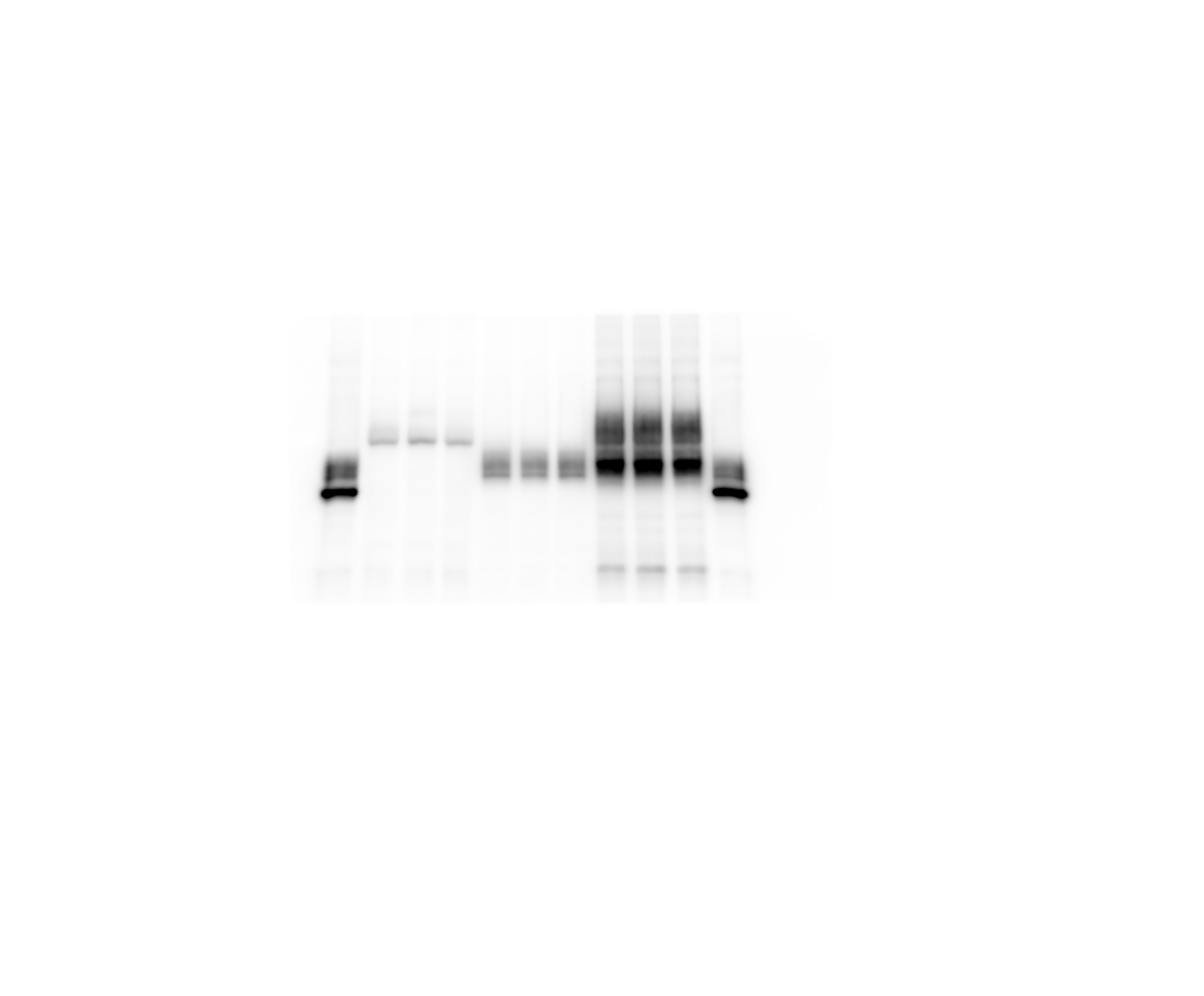

Supplement: Figure 1—source data 1. [file elife-103996-fig1-data1.zip › elife-103996-fig1-data1-v1/Figure 1B/V5(FZD9-10).tif]

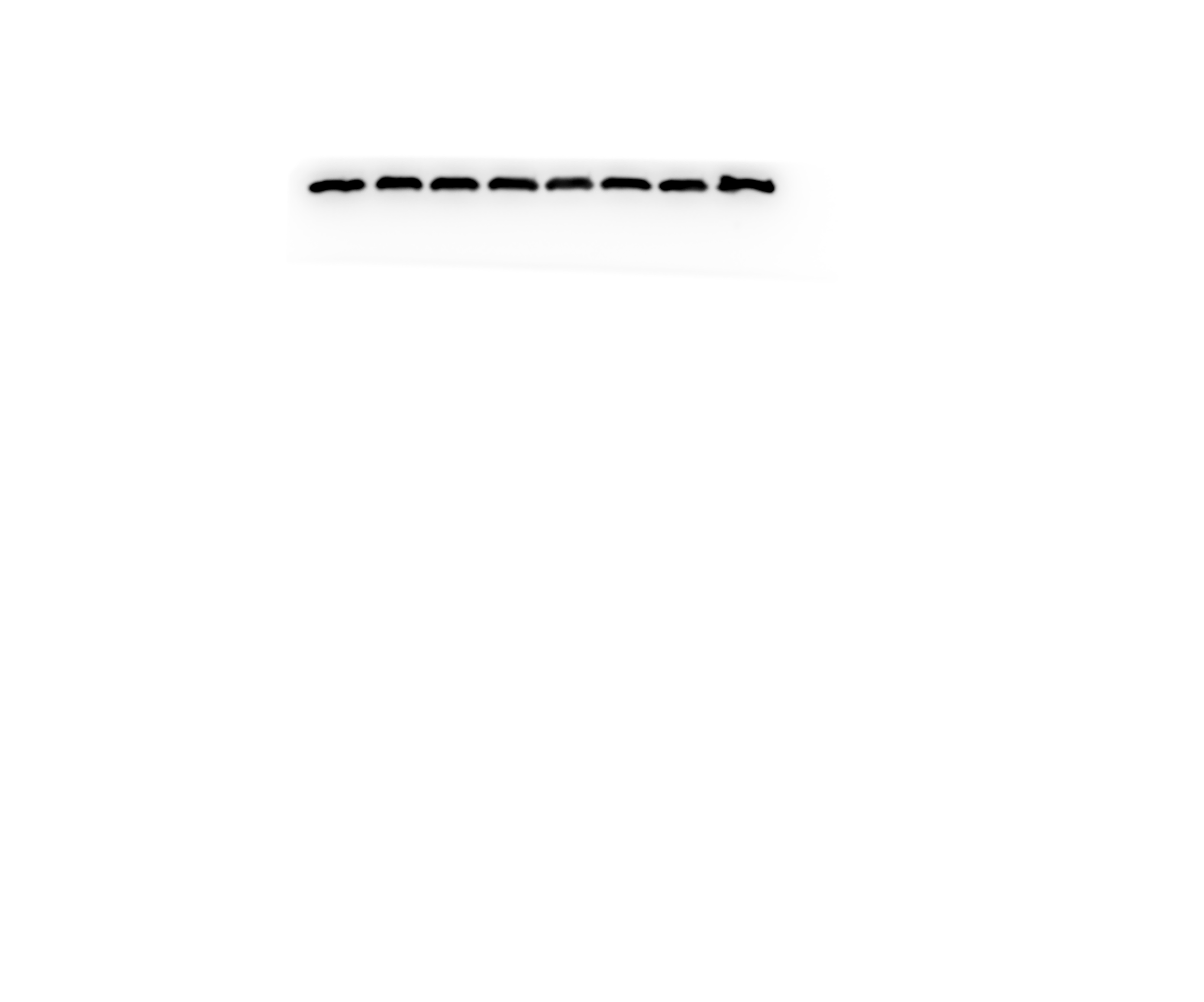

Supplement: Figure 1—source data 1. [file elife-103996-fig1-data1.zip › elife-103996-fig1-data1-v1/Figure 1C/Actin.tif]

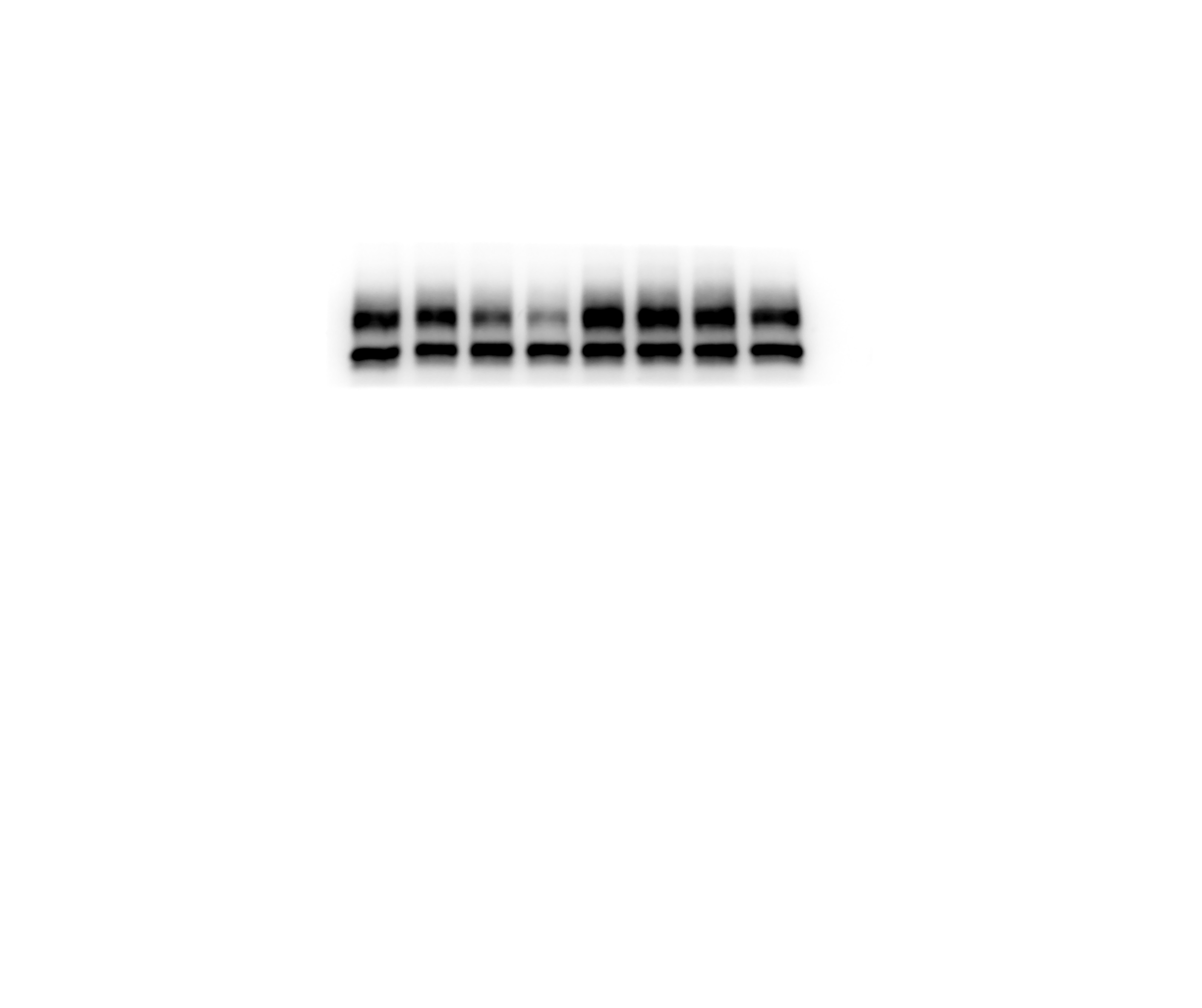

Supplement: Figure 1—source data 1. [file elife-103996-fig1-data1.zip › elife-103996-fig1-data1-v1/Figure 1C/V5.tif]

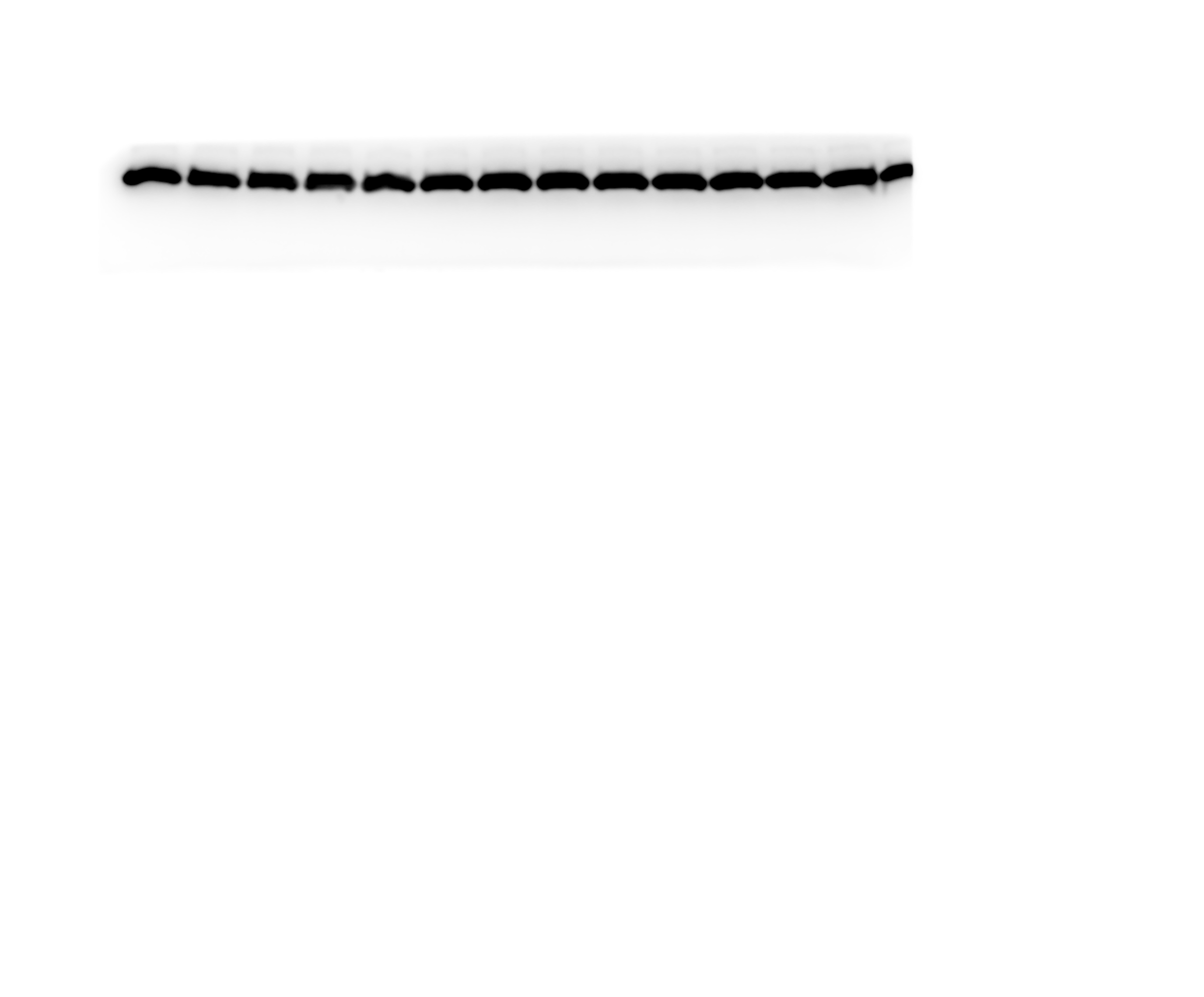

Supplement: Figure 1—figure supplement 1—source data 1. [file elife-103996-fig1-figsupp1-data1.zip › elife-103996-fig1-figsupp1-data1-v1/U2OS Actin(FZD1 FZD4).tif]

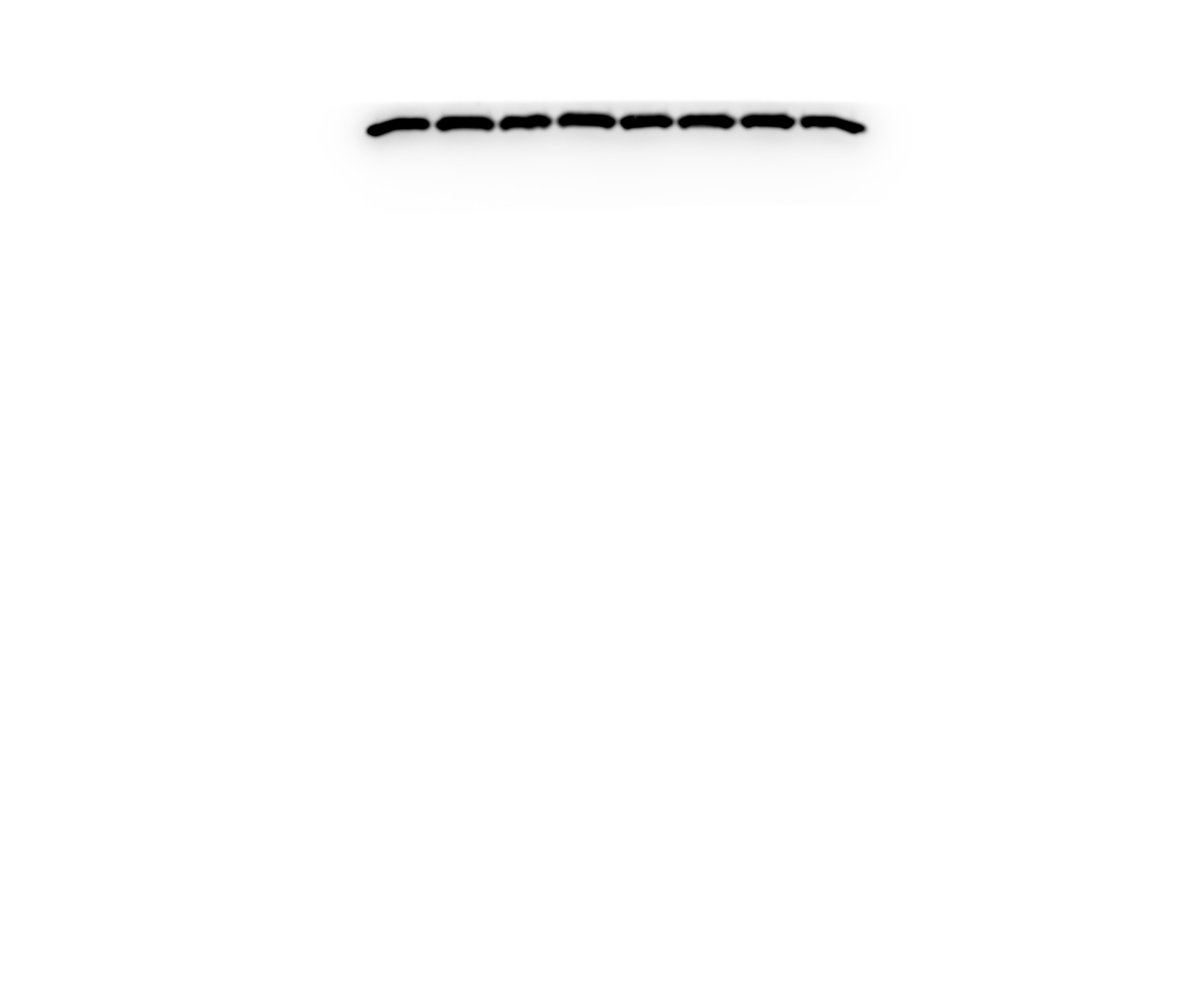

Supplement: Figure 1—figure supplement 1—source data 1. [file elife-103996-fig1-figsupp1-data1.zip › elife-103996-fig1-figsupp1-data1-v1/U2OS Actin(FZD5).tif]

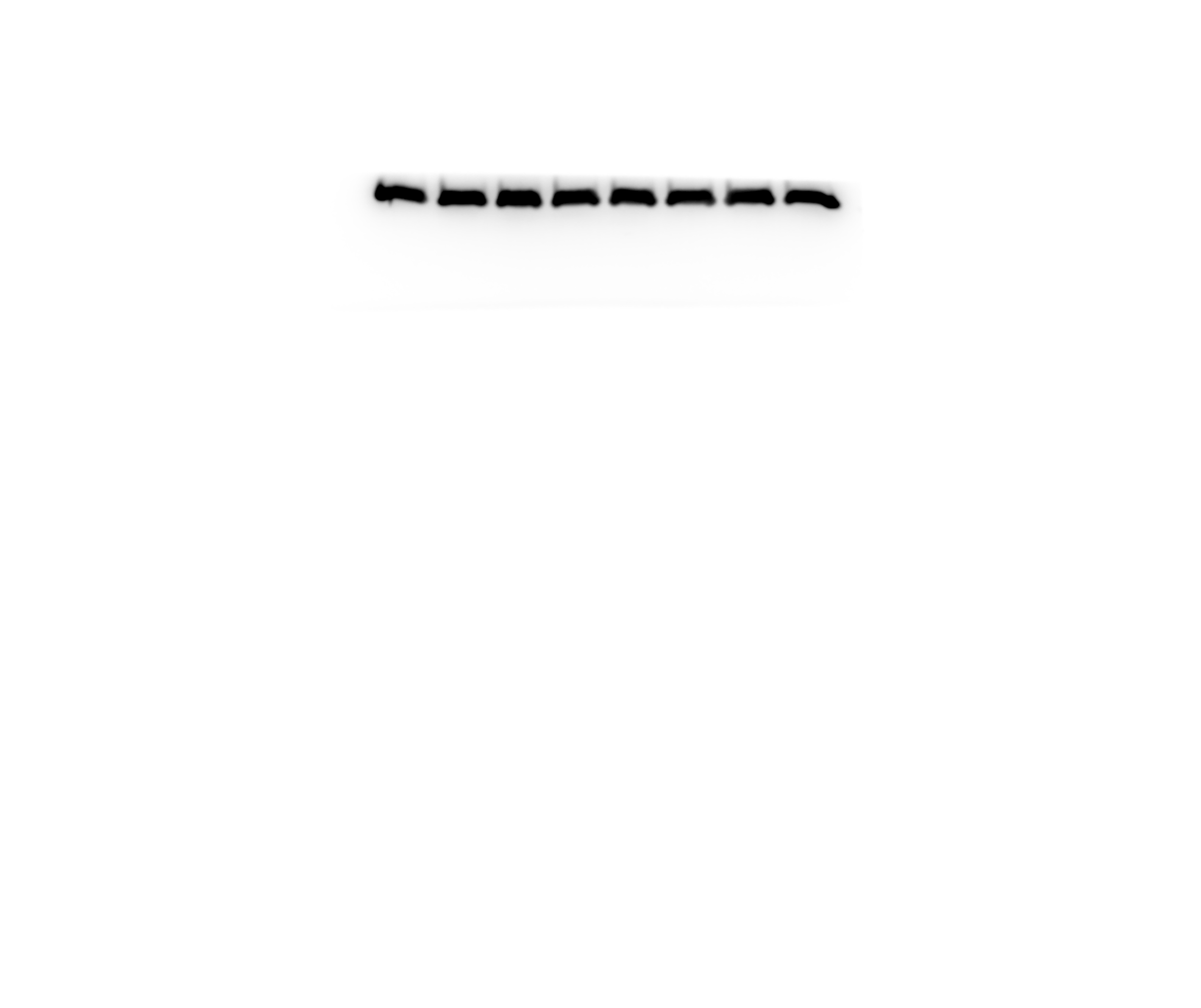

Supplement: Figure 1—figure supplement 1—source data 1. [file elife-103996-fig1-figsupp1-data1.zip › elife-103996-fig1-figsupp1-data1-v1/U2OS Actin(FZD7).tif]

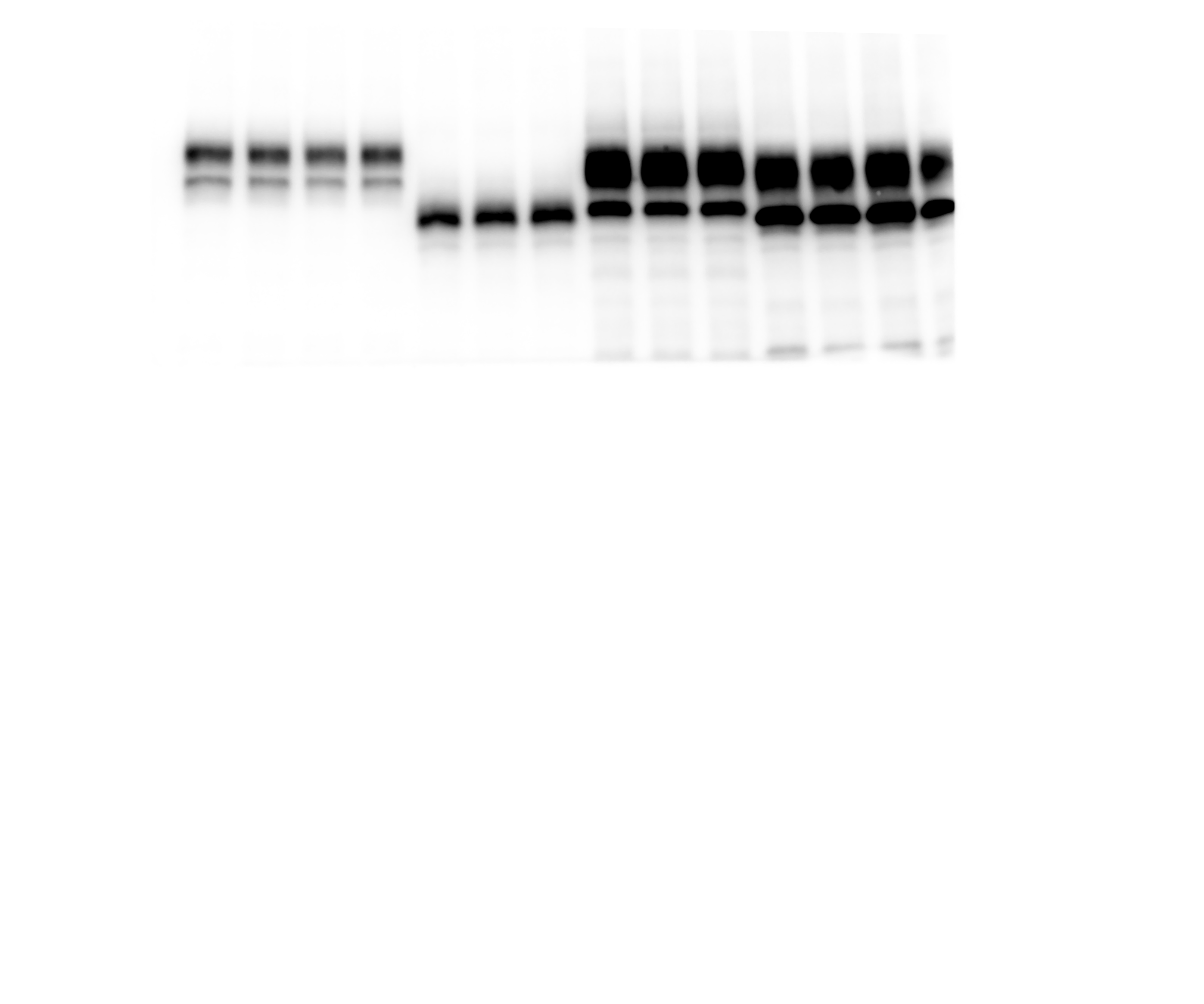

Supplement: Figure 1—figure supplement 1—source data 1. [file elife-103996-fig1-figsupp1-data1.zip › elife-103996-fig1-figsupp1-data1-v1/U2OS V5(FZD1 FZD4).tif]

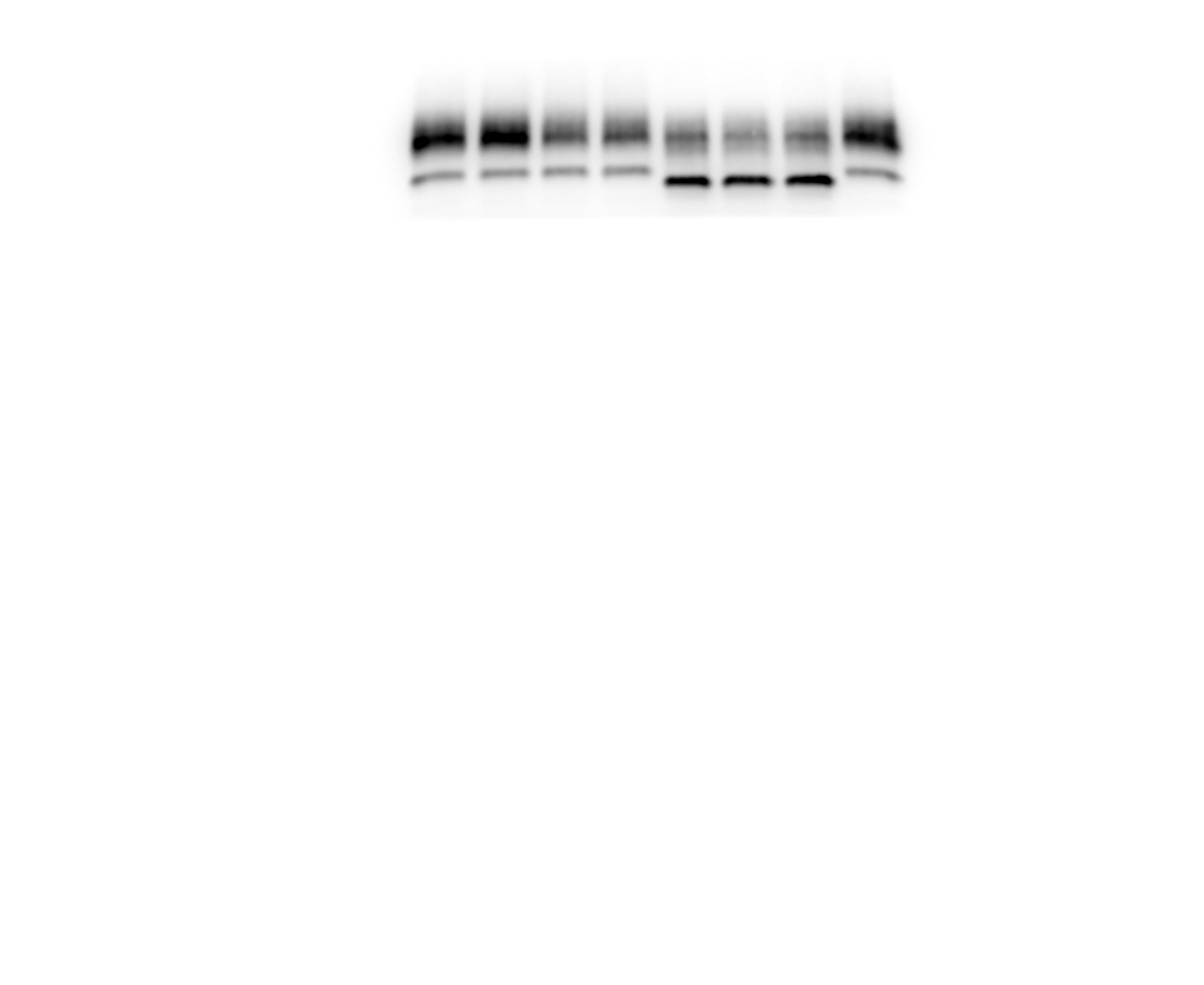

Supplement: Figure 1—figure supplement 1—source data 1. [file elife-103996-fig1-figsupp1-data1.zip › elife-103996-fig1-figsupp1-data1-v1/U2OS V5(FZD5).tif]

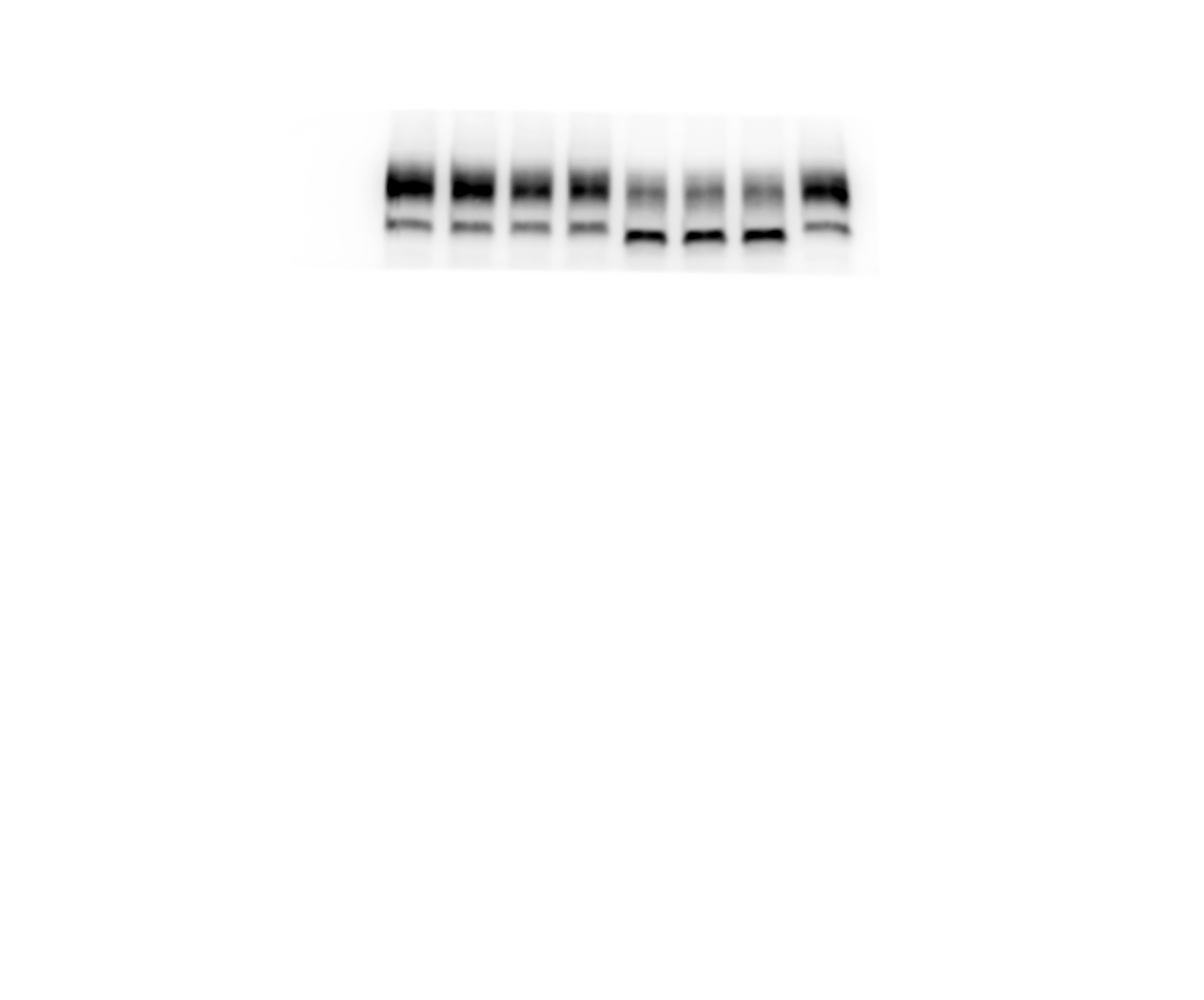

Supplement: Figure 1—figure supplement 1—source data 1. [file elife-103996-fig1-figsupp1-data1.zip › elife-103996-fig1-figsupp1-data1-v1/U2OS V5(FZD7).tif]

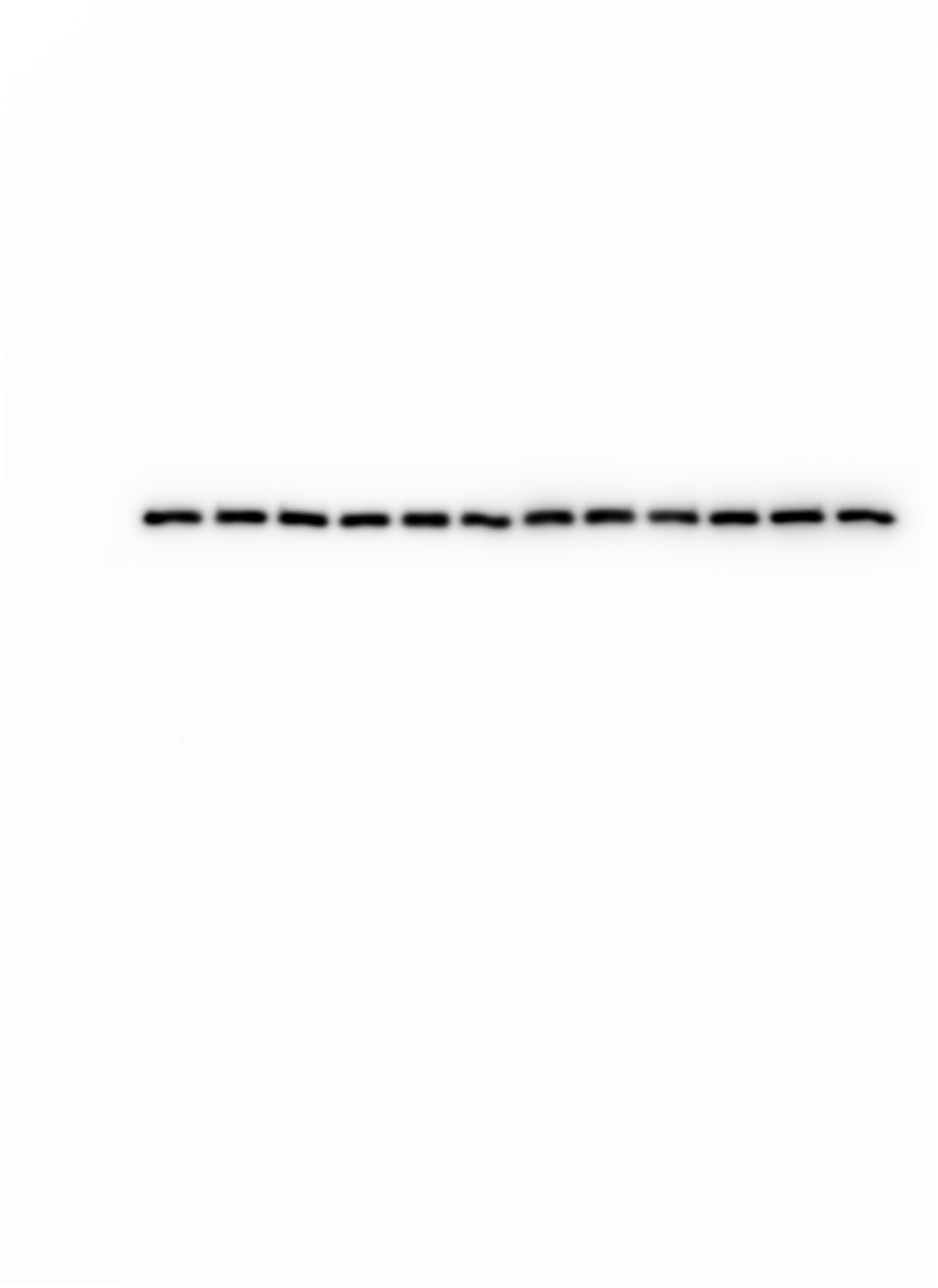

Supplement: Figure 2—source data 1. [file elife-103996-fig2-data1.zip › elife-103996-fig2-data1-v1/Figure 2A/Actin.tif]

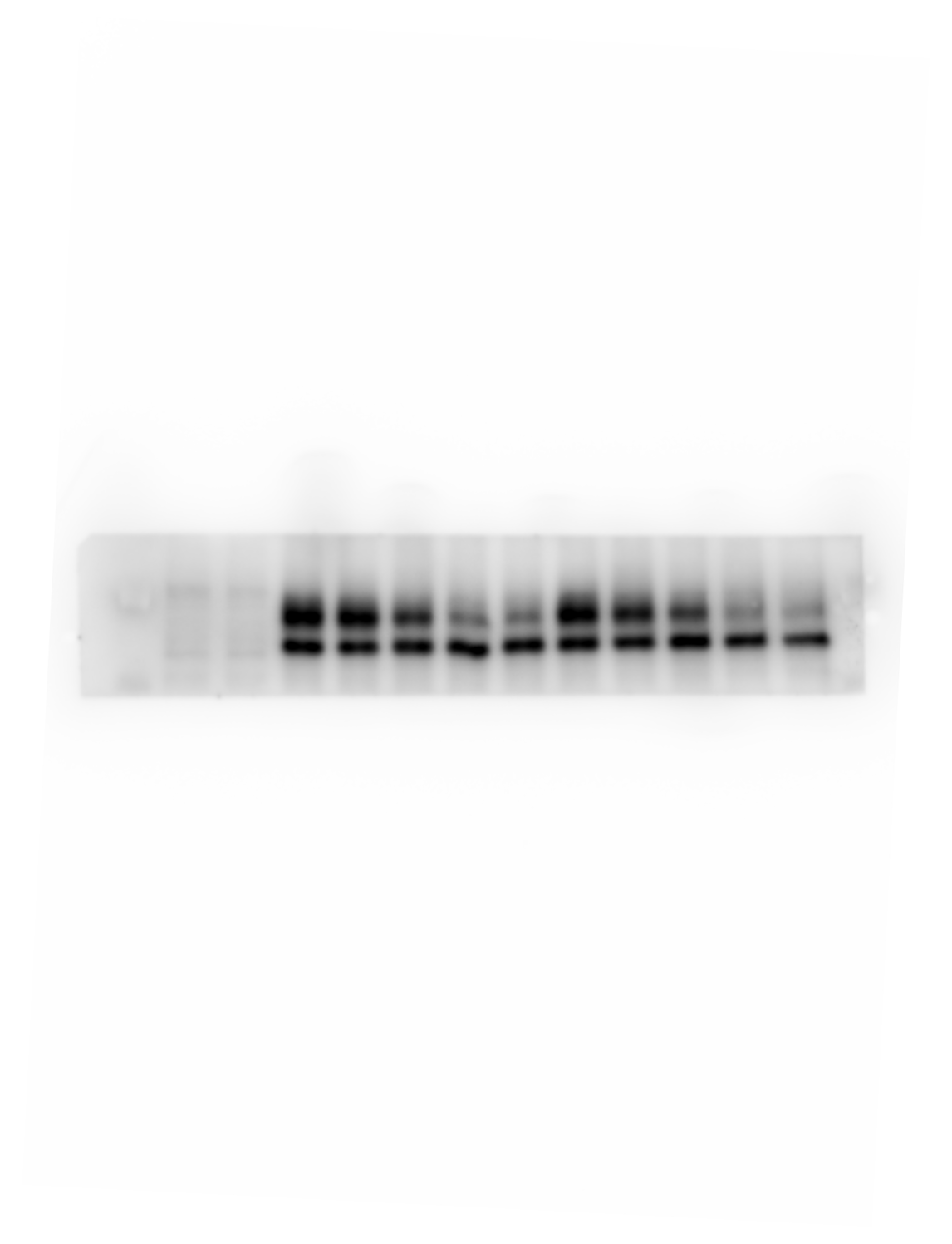

Supplement: Figure 2—source data 1. [file elife-103996-fig2-data1.zip › elife-103996-fig2-data1-v1/Figure 2A/V5.tif]

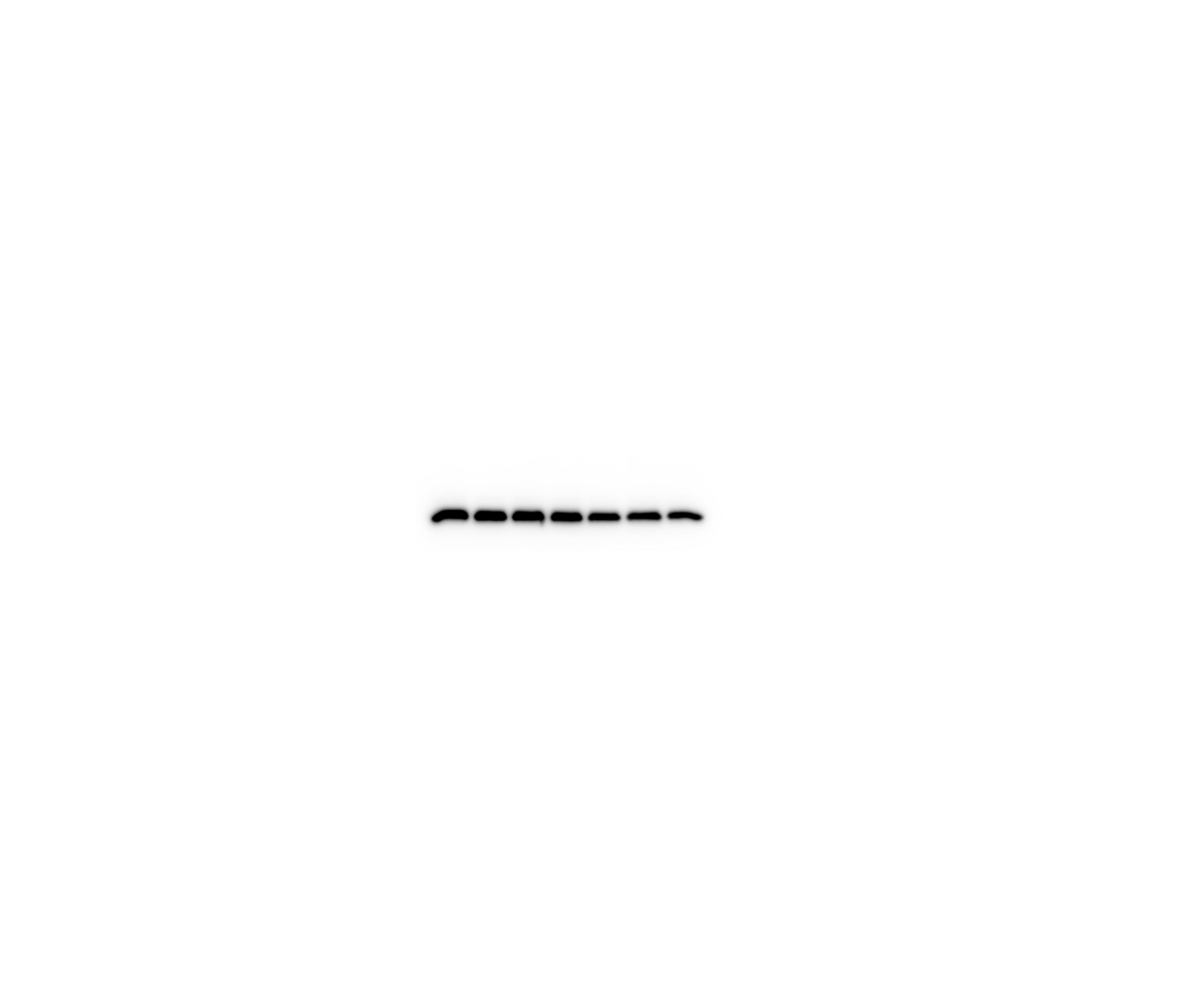

Supplement: Figure 2—source data 1. [file elife-103996-fig2-data1.zip › elife-103996-fig2-data1-v1/Figure 2B/Figure 2B Actin.tif]

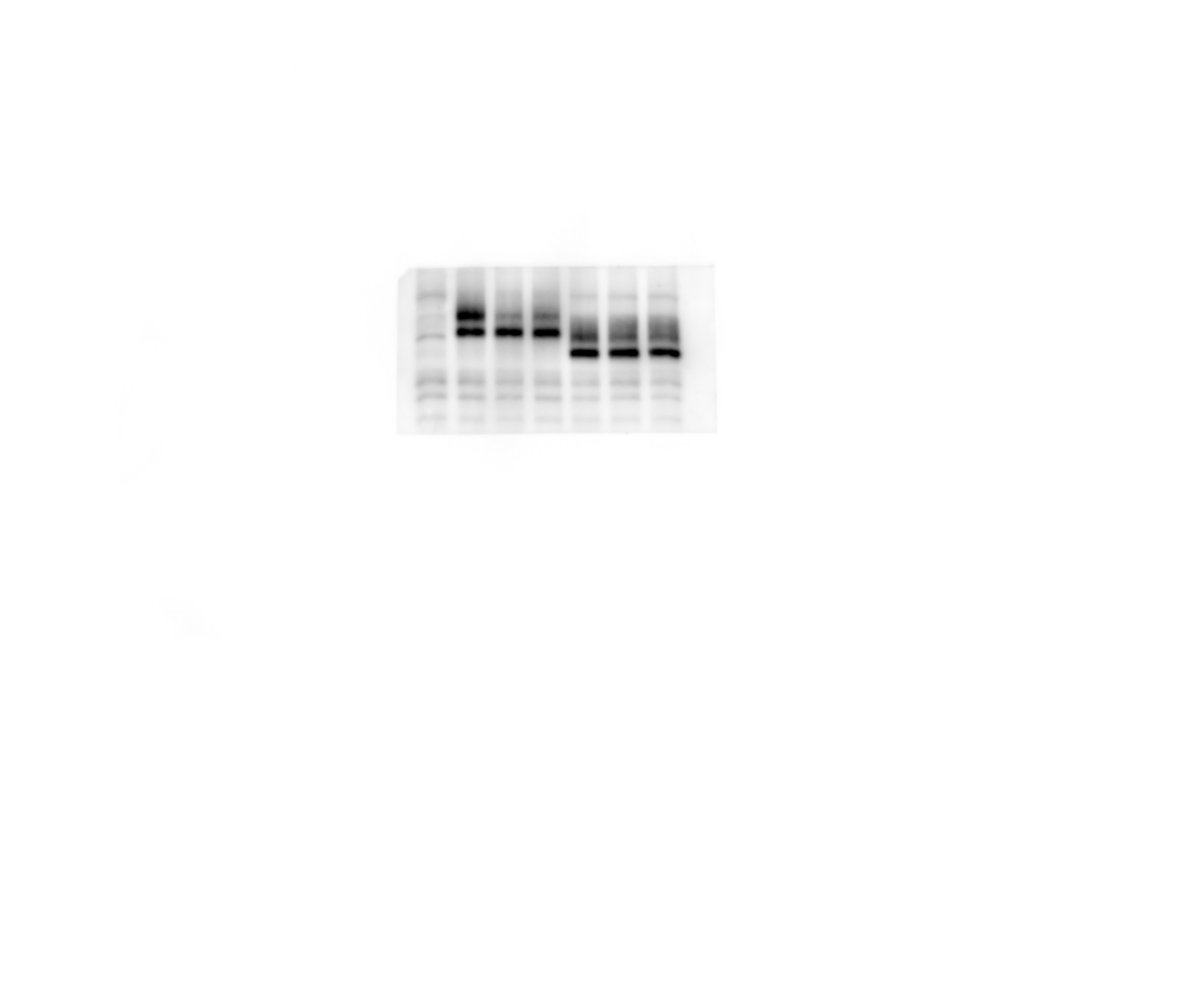

Supplement: Figure 2—source data 1. [file elife-103996-fig2-data1.zip › elife-103996-fig2-data1-v1/Figure 2B/Figure 2B V5.tif]

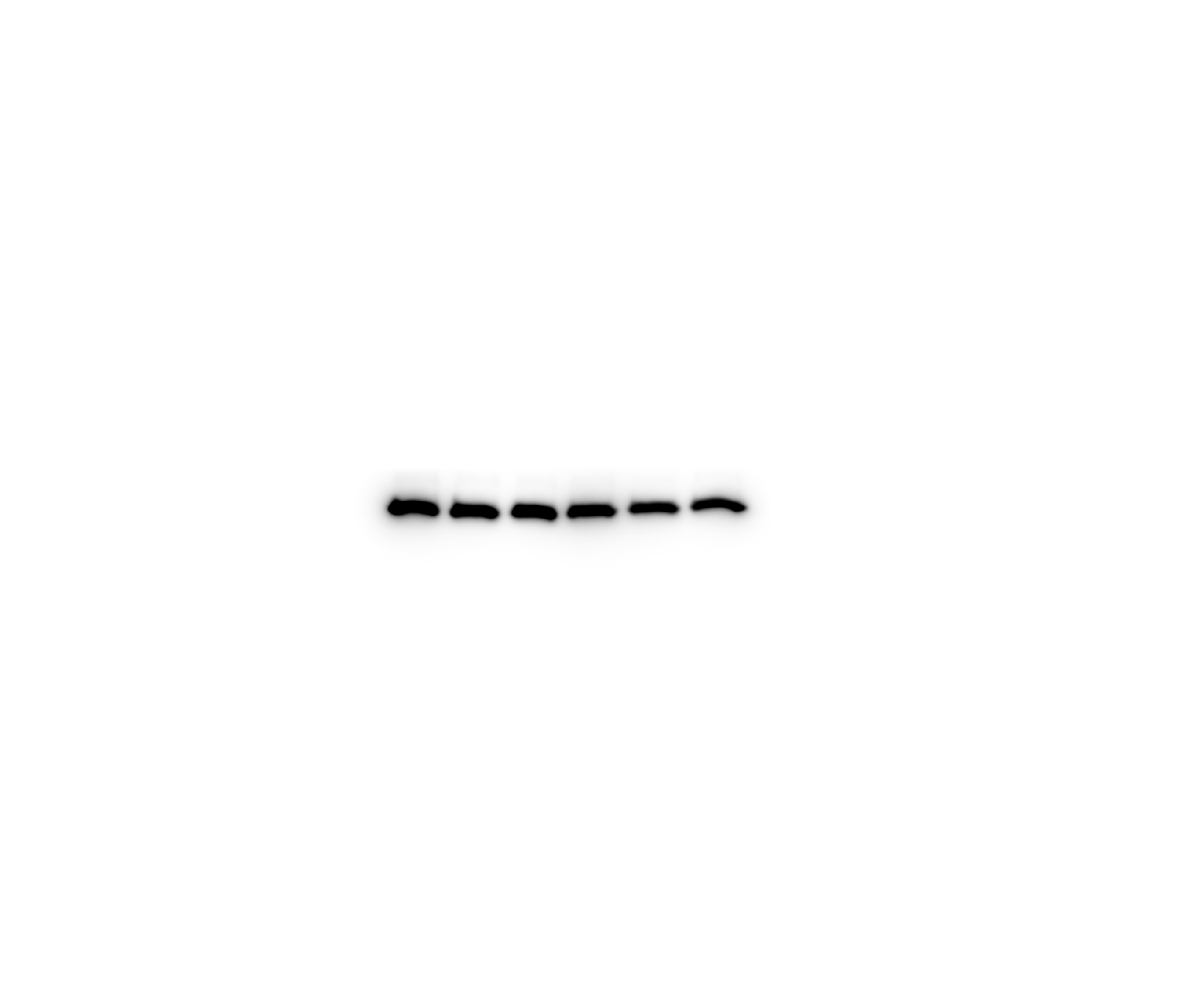

Supplement: Figure 2—source data 1. [file elife-103996-fig2-data1.zip › elife-103996-fig2-data1-v1/Figure 2C/Figure 2C Actin.tif]

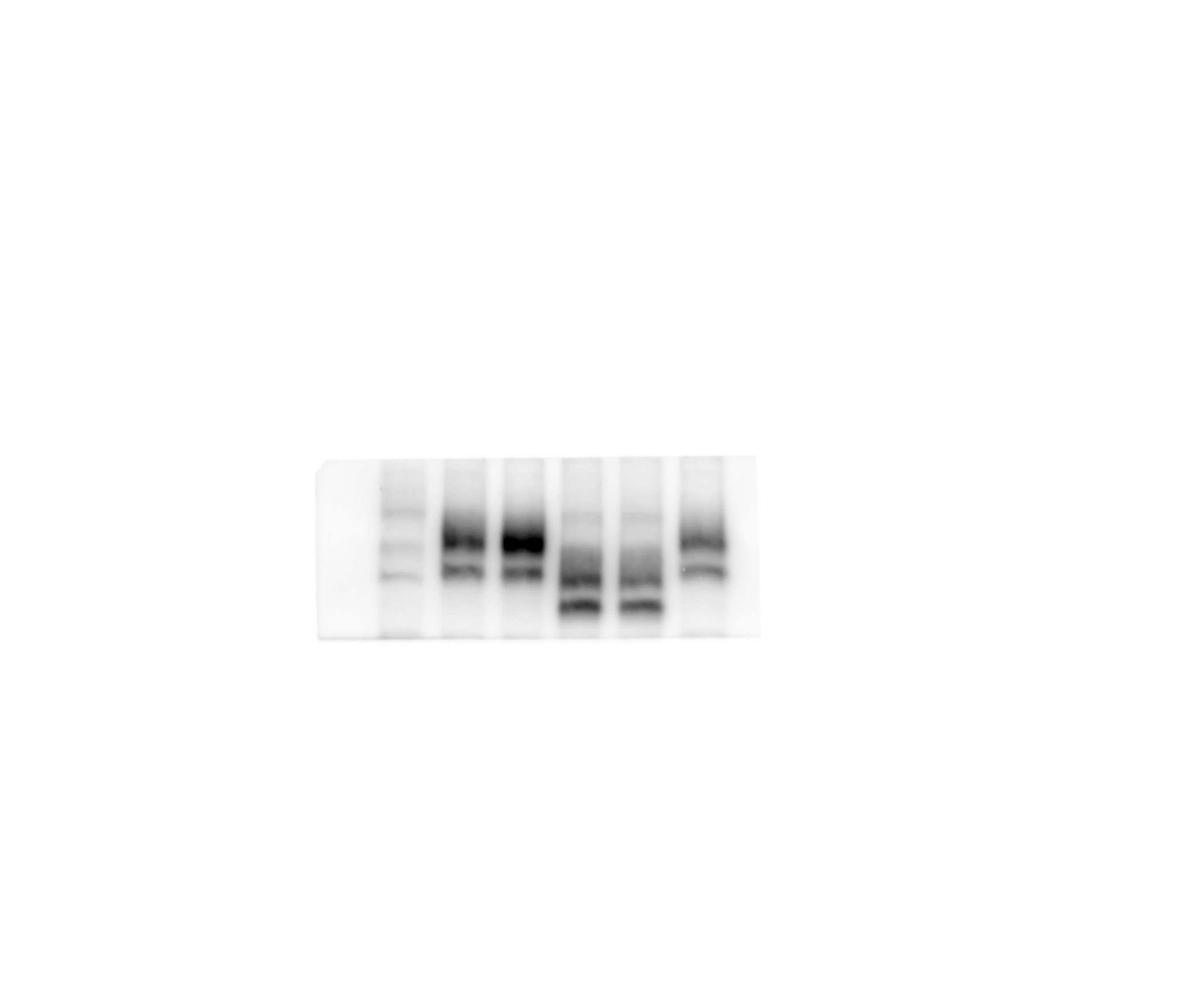

Supplement: Figure 2—source data 1. [file elife-103996-fig2-data1.zip › elife-103996-fig2-data1-v1/Figure 2C/Figure 2C V5.tif]

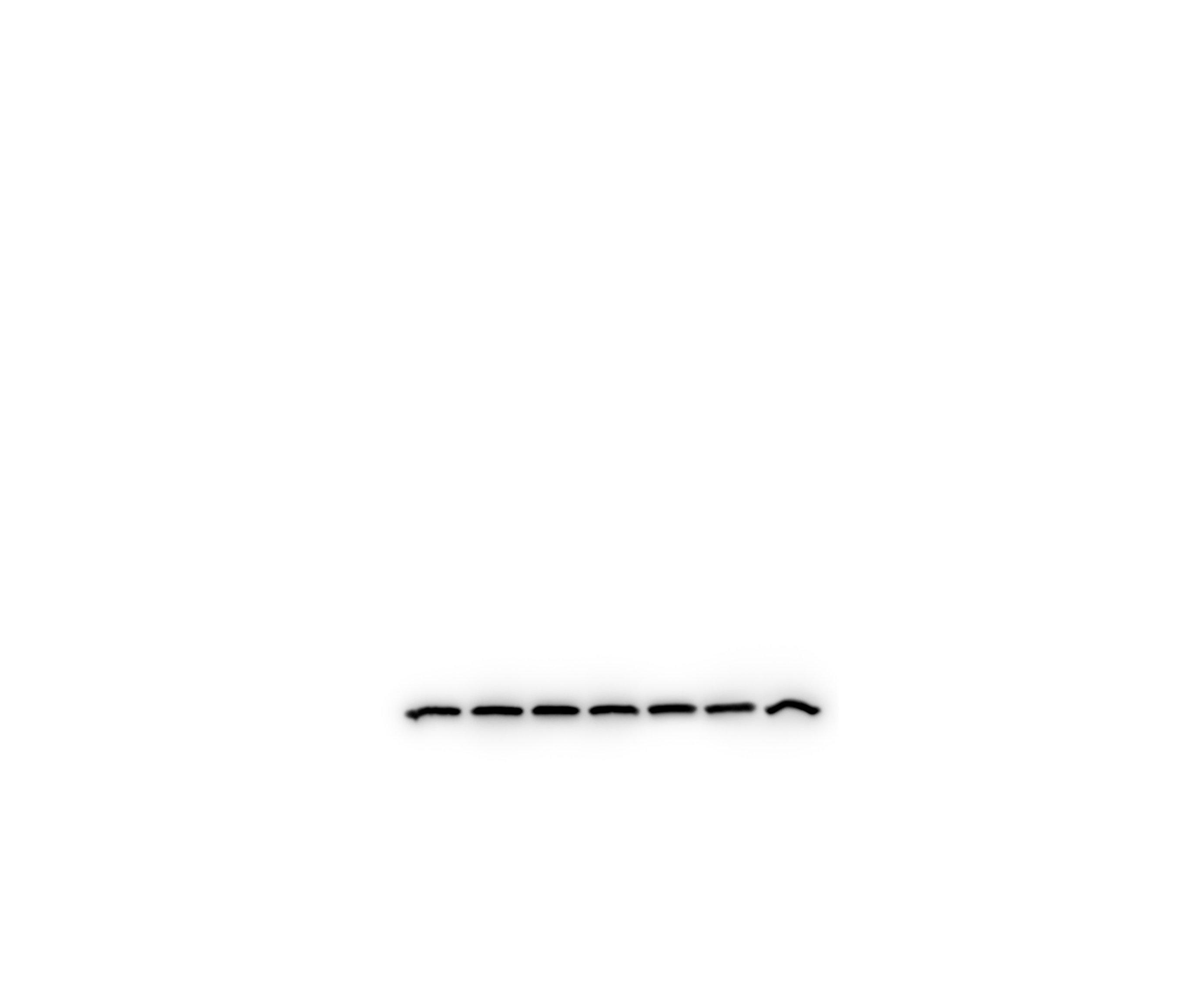

Supplement: Figure 2—source data 1. [file elife-103996-fig2-data1.zip › elife-103996-fig2-data1-v1/Figure 2H/Figure 2H Actin.tif]

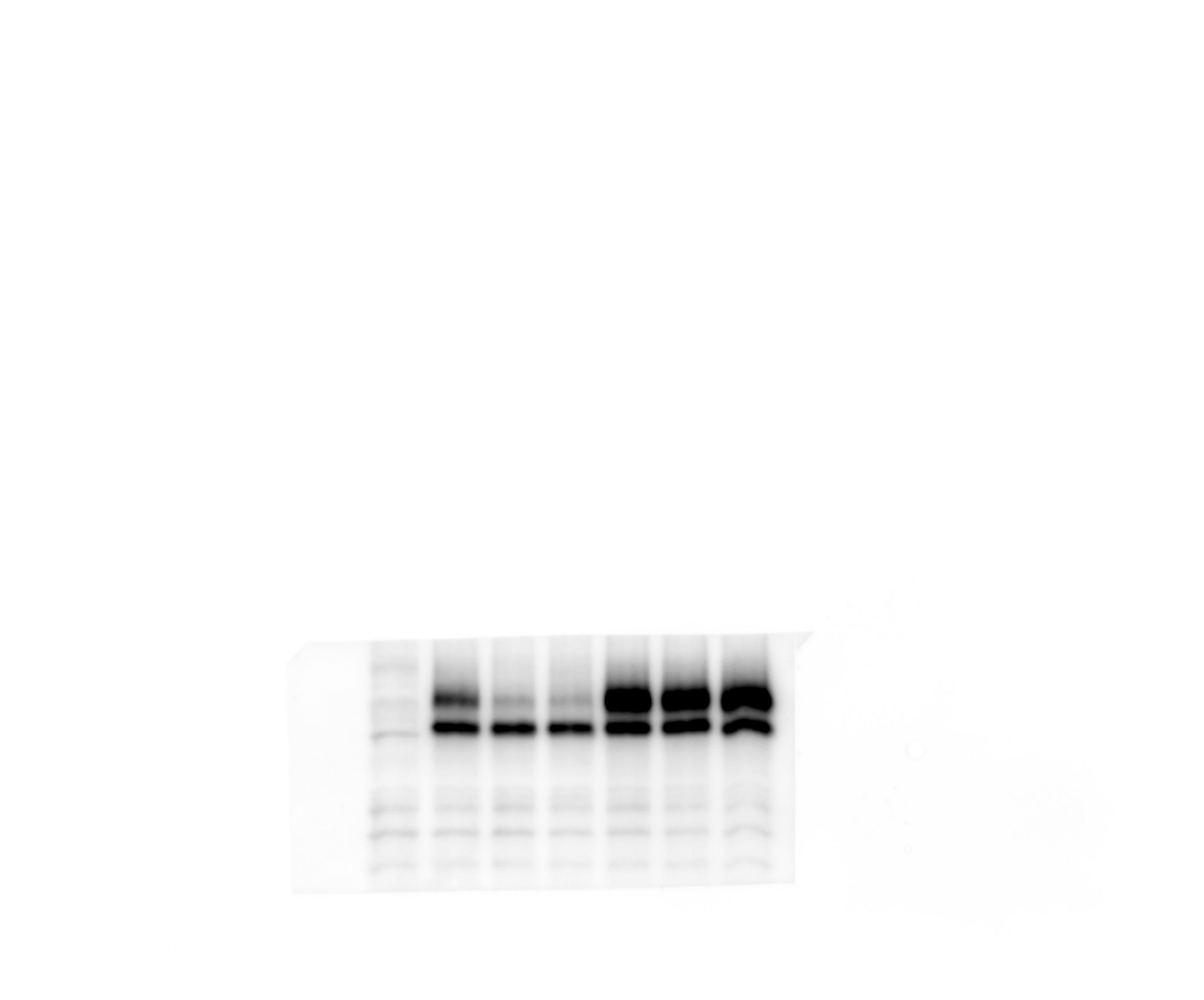

Supplement: Figure 2—source data 1. [file elife-103996-fig2-data1.zip › elife-103996-fig2-data1-v1/Figure 2H/Figure 2H V5.tif]

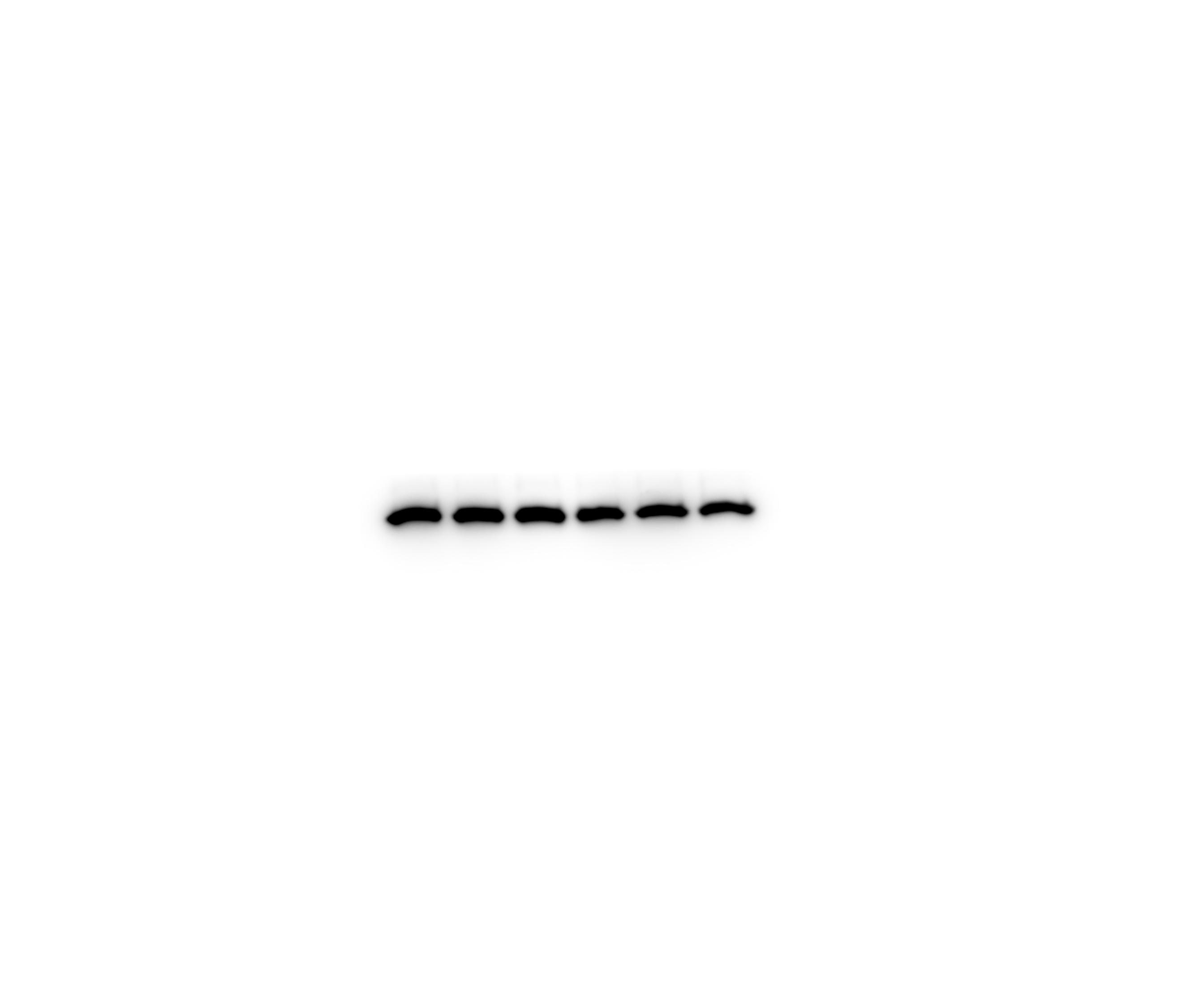

Supplement: Figure 2—source data 1. [file elife-103996-fig2-data1.zip › elife-103996-fig2-data1-v1/Figure 2I/Figure 2I Actin.tif]

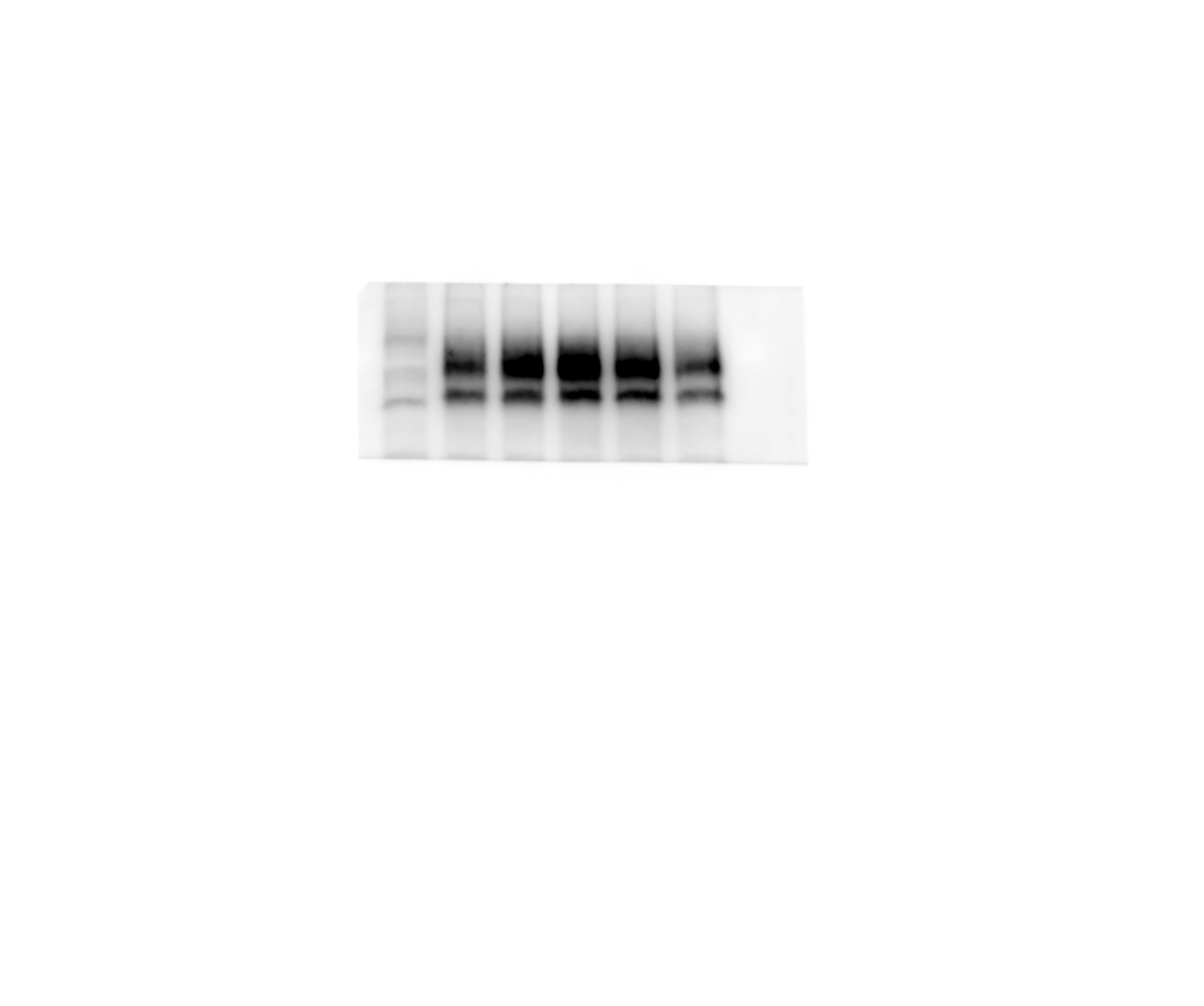

Supplement: Figure 2—source data 1. [file elife-103996-fig2-data1.zip › elife-103996-fig2-data1-v1/Figure 2I/Figure 2I V5.tif]

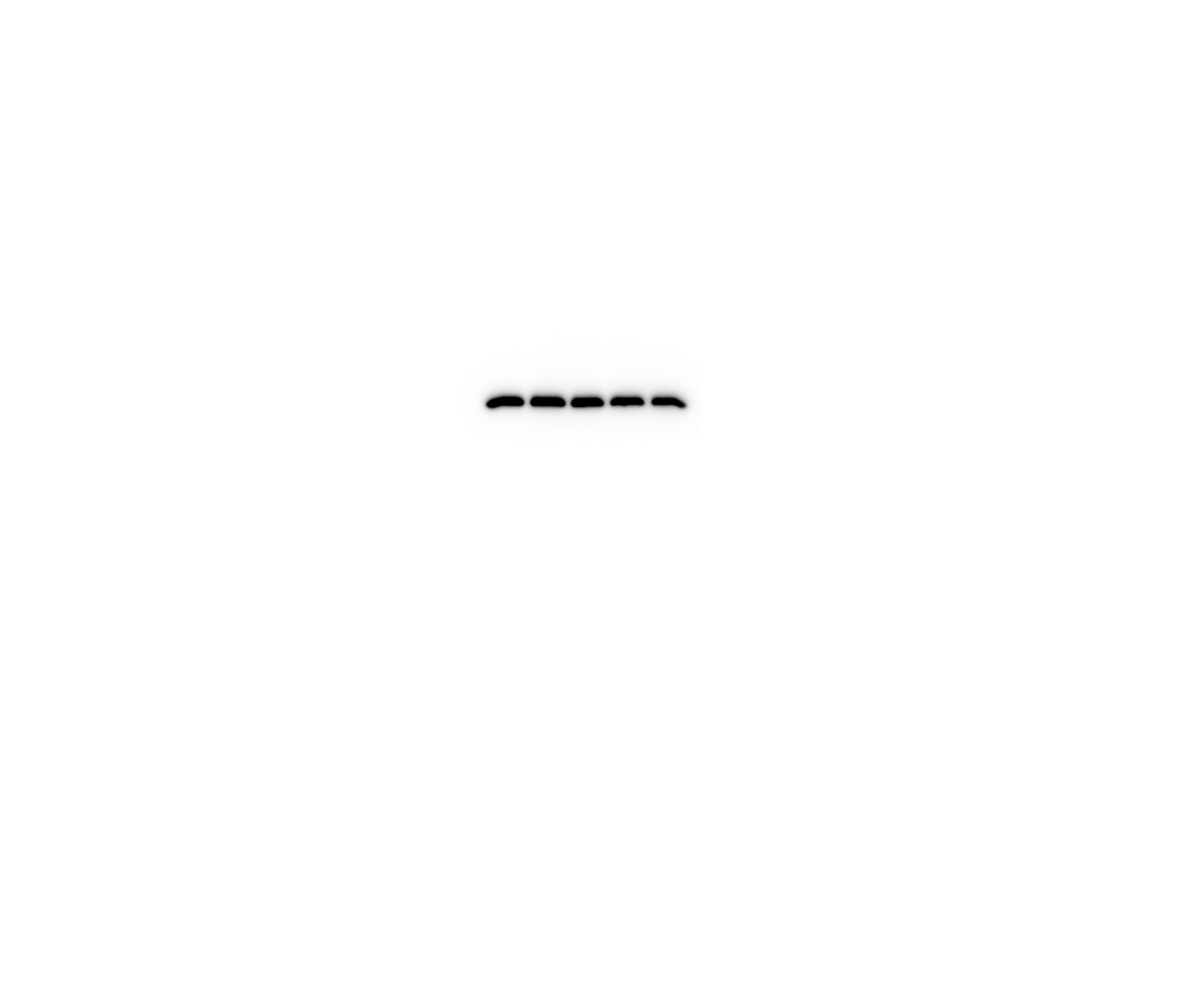

Supplement: Figure 2—source data 1. [file elife-103996-fig2-data1.zip › elife-103996-fig2-data1-v1/Figure 2J/Figure 2J Actin.tif]

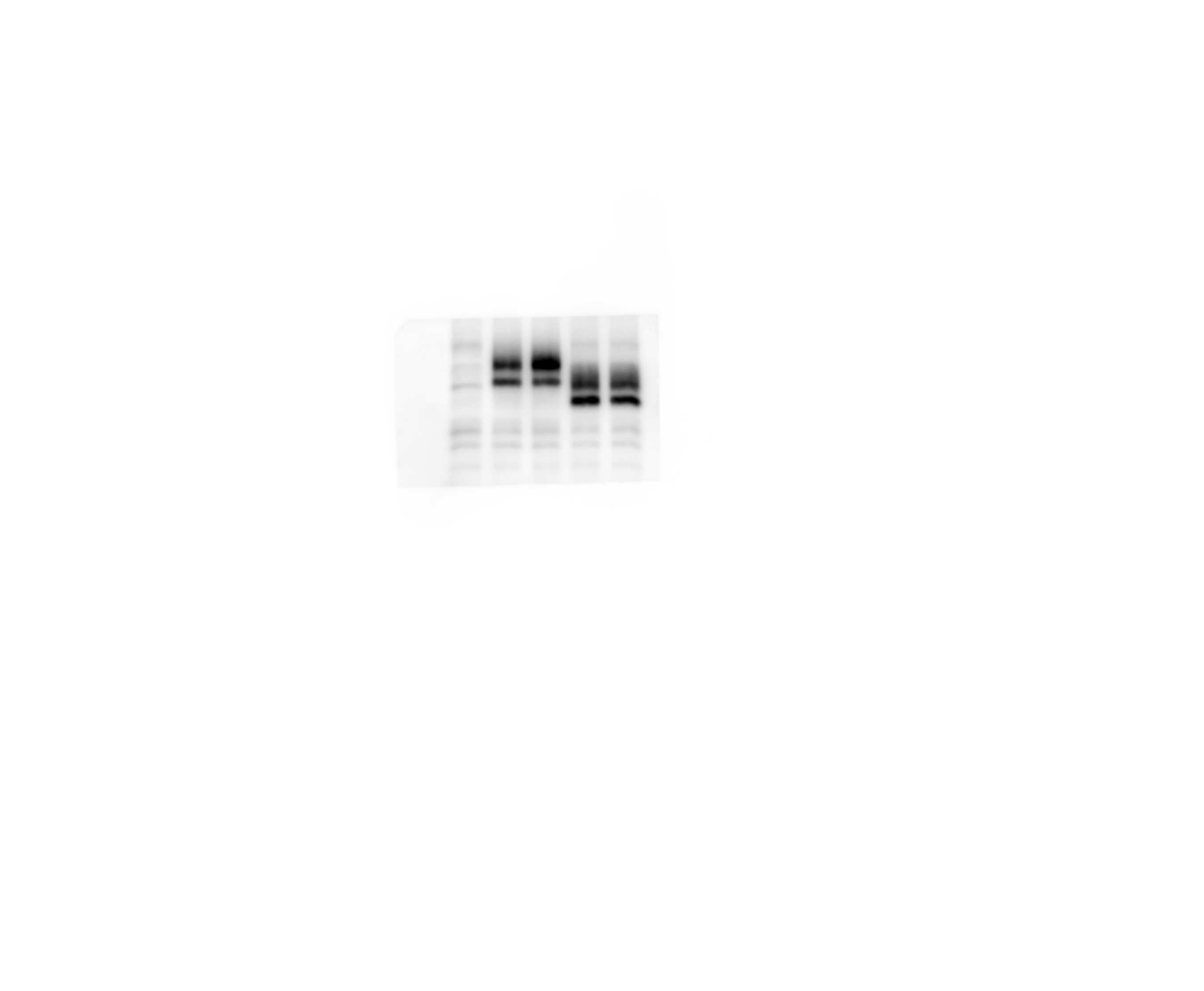

Supplement: Figure 2—source data 1. [file elife-103996-fig2-data1.zip › elife-103996-fig2-data1-v1/Figure 2J/Figure 2J V5.tif]

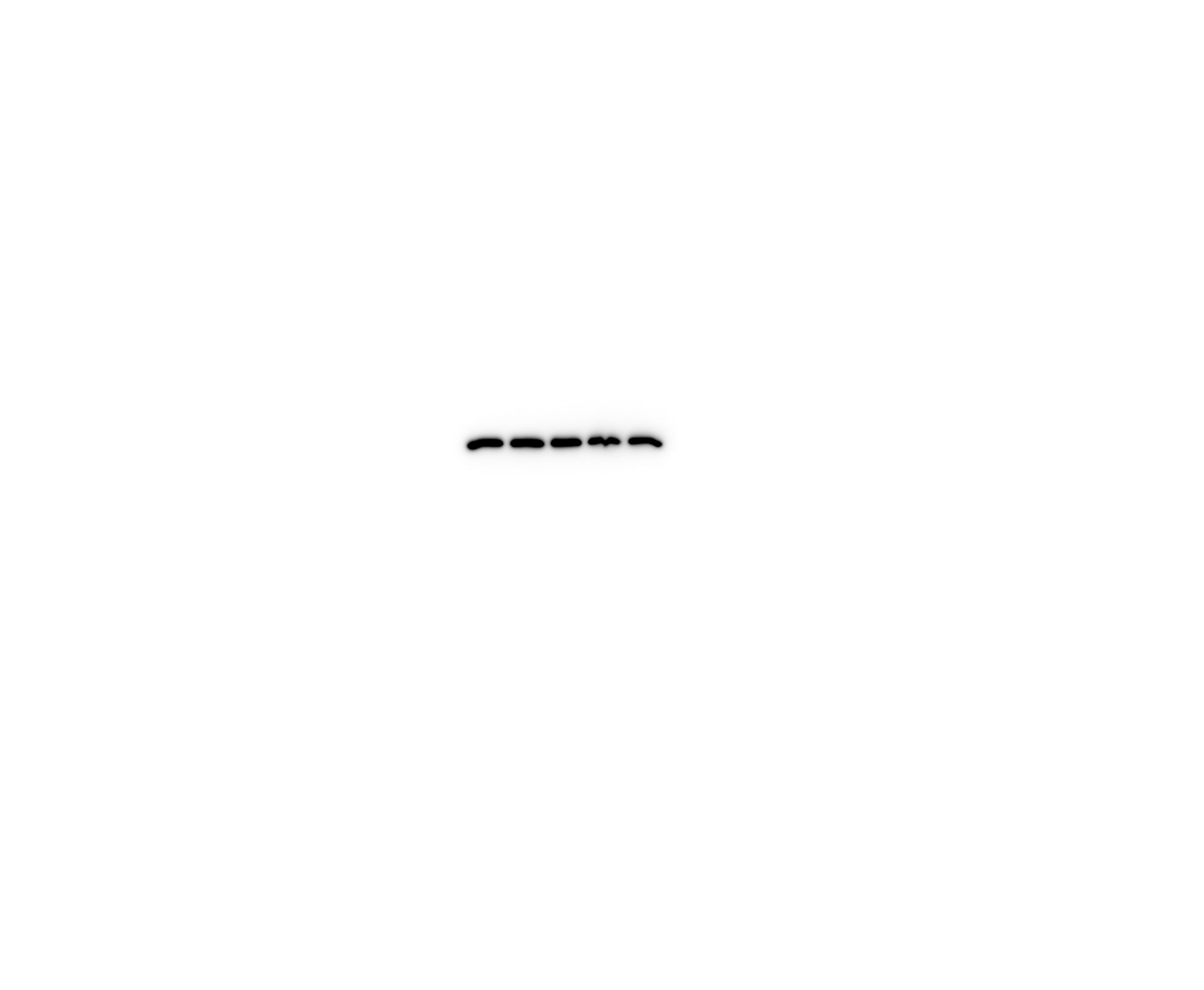

Supplement: Figure 2—source data 1. [file elife-103996-fig2-data1.zip › elife-103996-fig2-data1-v1/Figure 2K/Figure 2K Actin.tif]

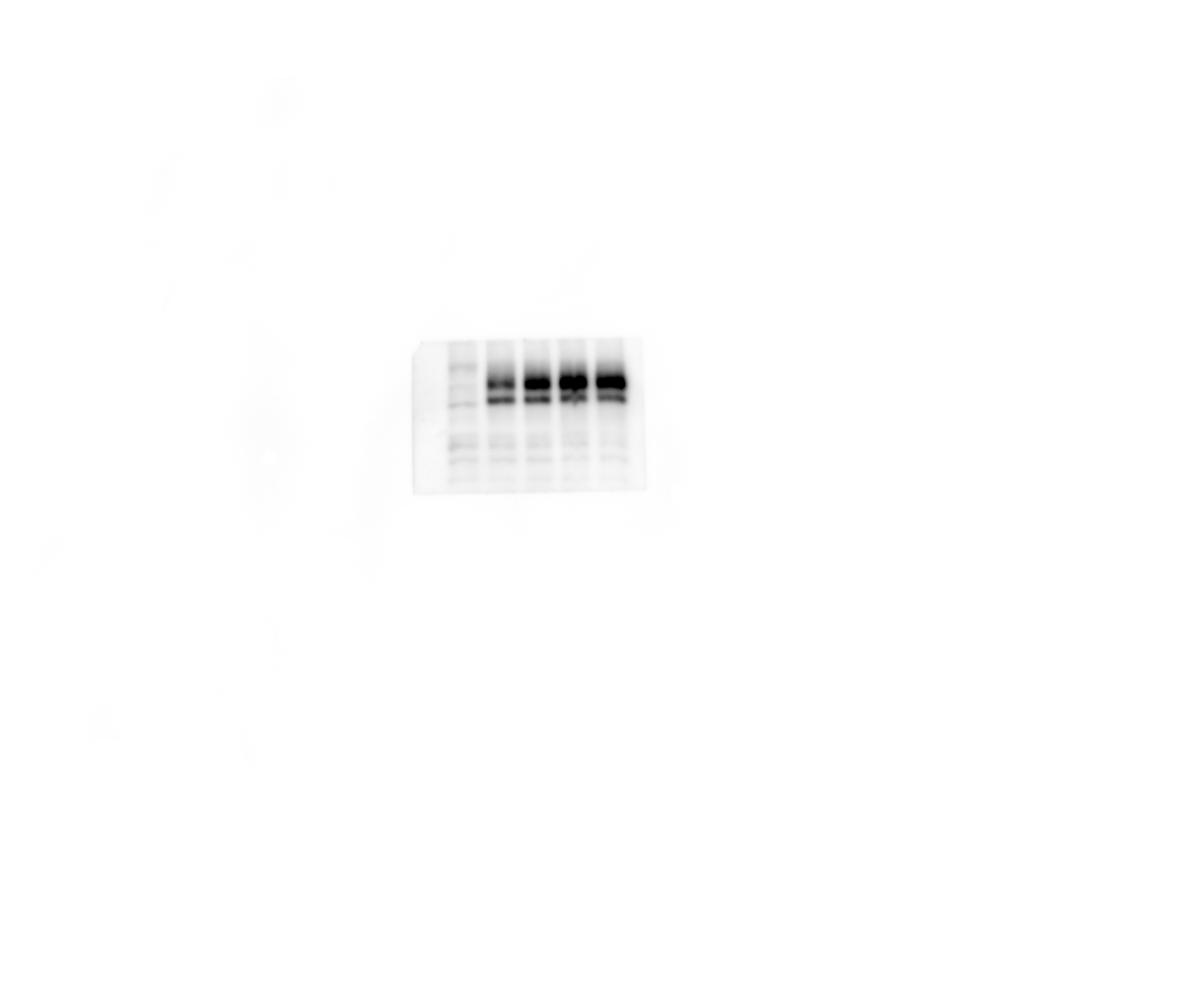

Supplement: Figure 2—source data 1. [file elife-103996-fig2-data1.zip › elife-103996-fig2-data1-v1/Figure 2K/Figure 2K V5.tif]

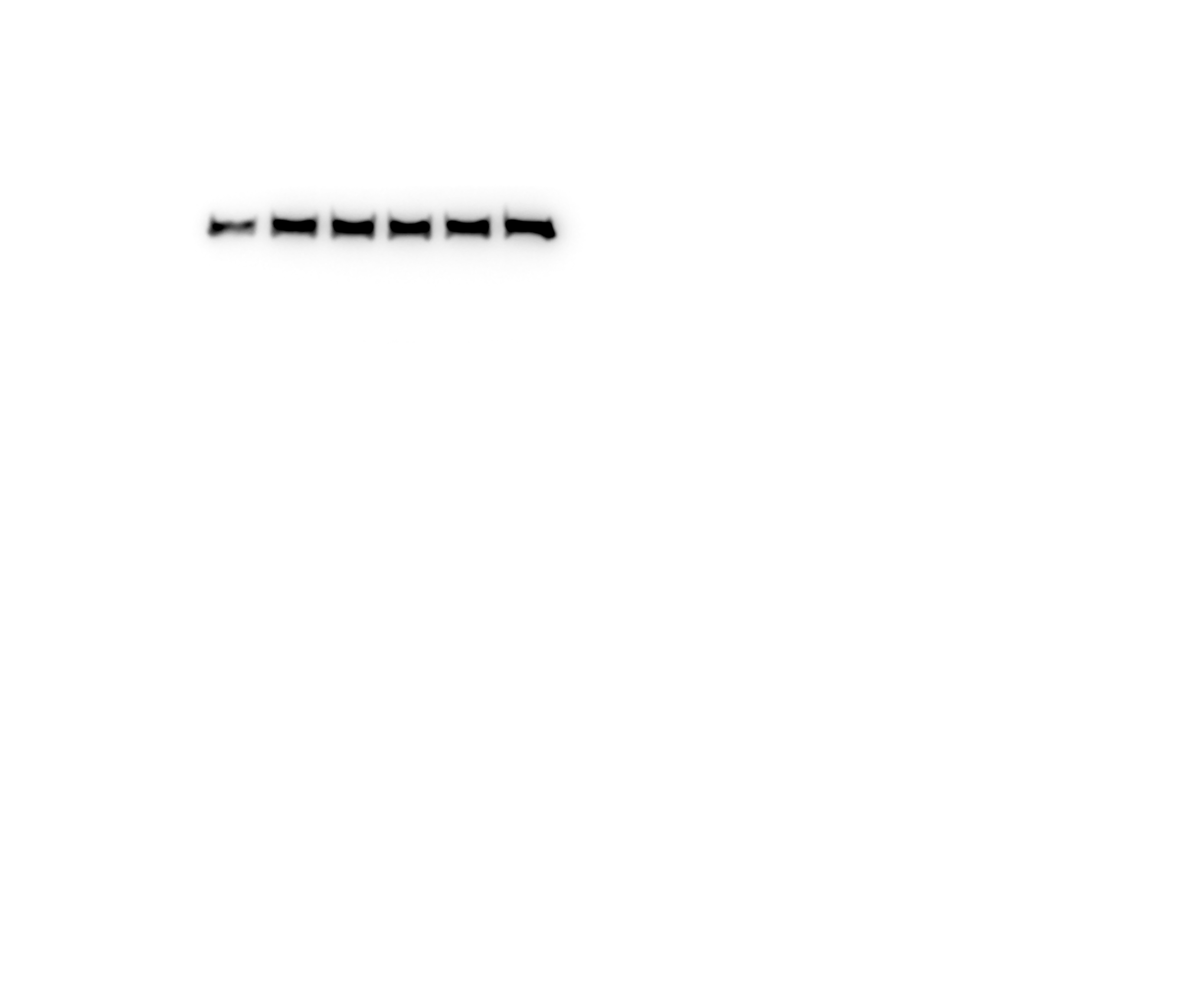

Supplement: Figure 2—source data 1. [file elife-103996-fig2-data1.zip › elife-103996-fig2-data1-v1/Figure 2L/Figure 2L Actin.tif]

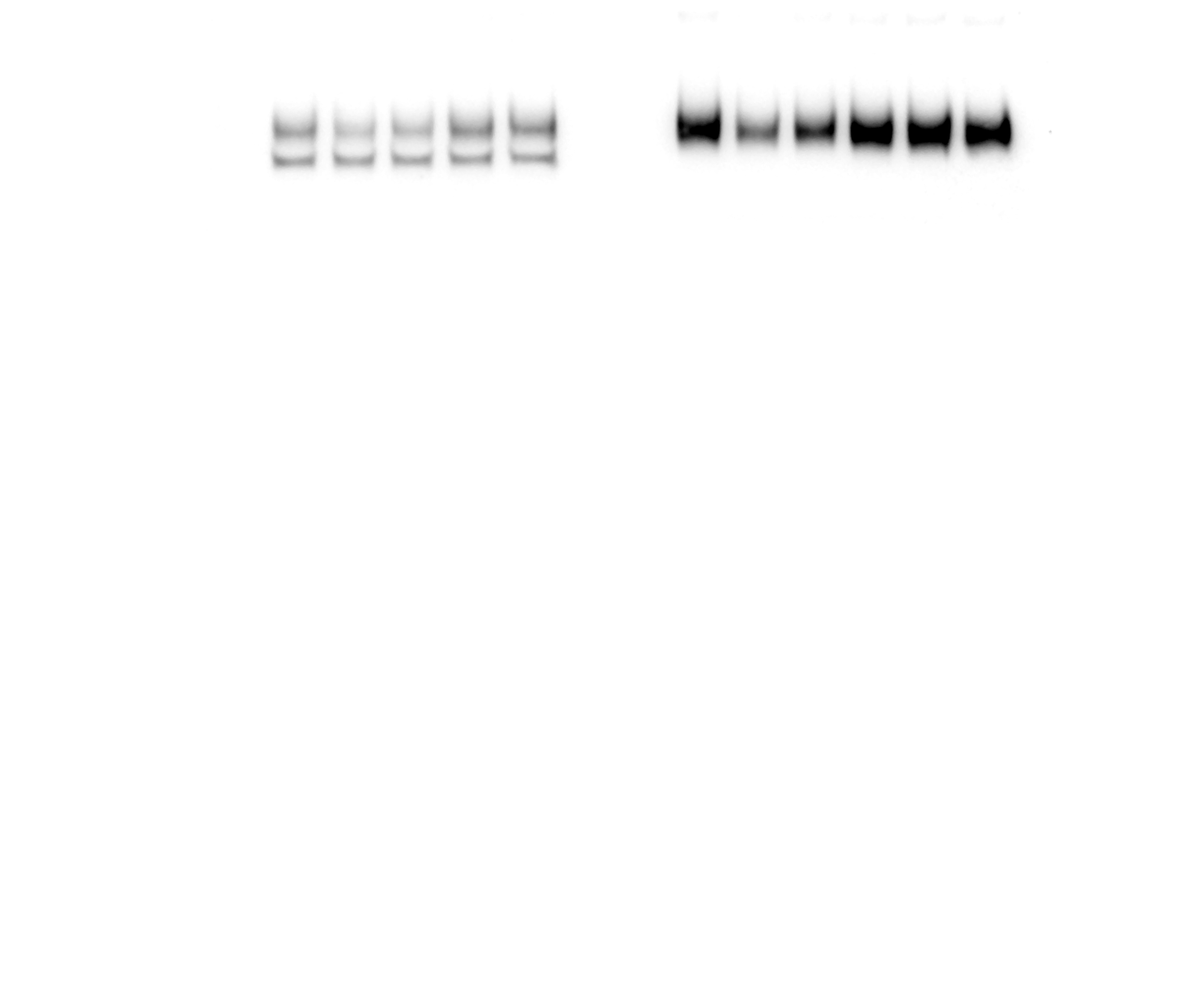

Supplement: Figure 2—source data 1. [file elife-103996-fig2-data1.zip › elife-103996-fig2-data1-v1/Figure 2L/Figure 2L V5.tif]

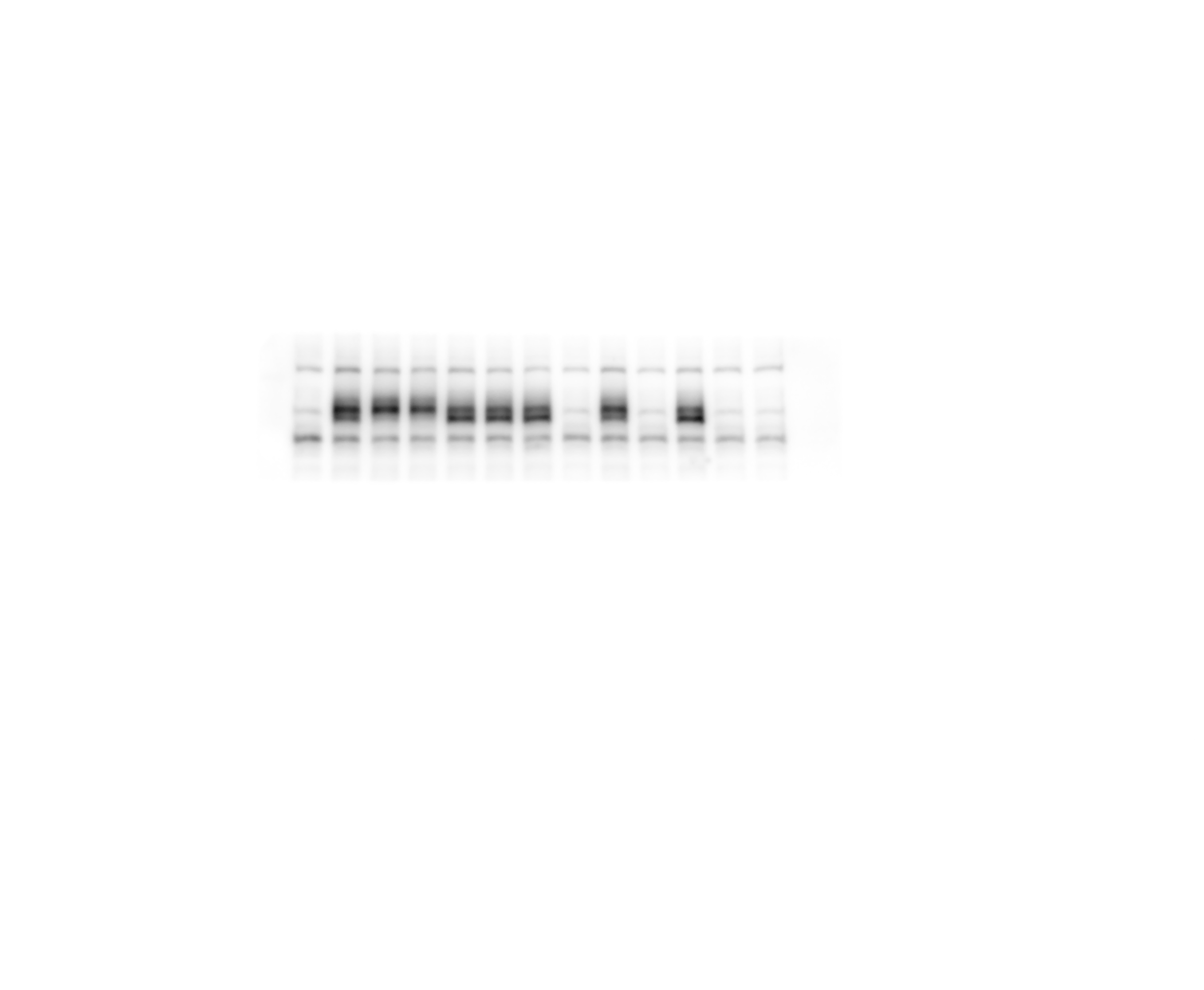

Supplement: Figure 3—source data 1. [file elife-103996-fig3-data1.zip › elife-103996-fig3-data1-v1/Figure 3A/Figure 3A DVL1.tif]

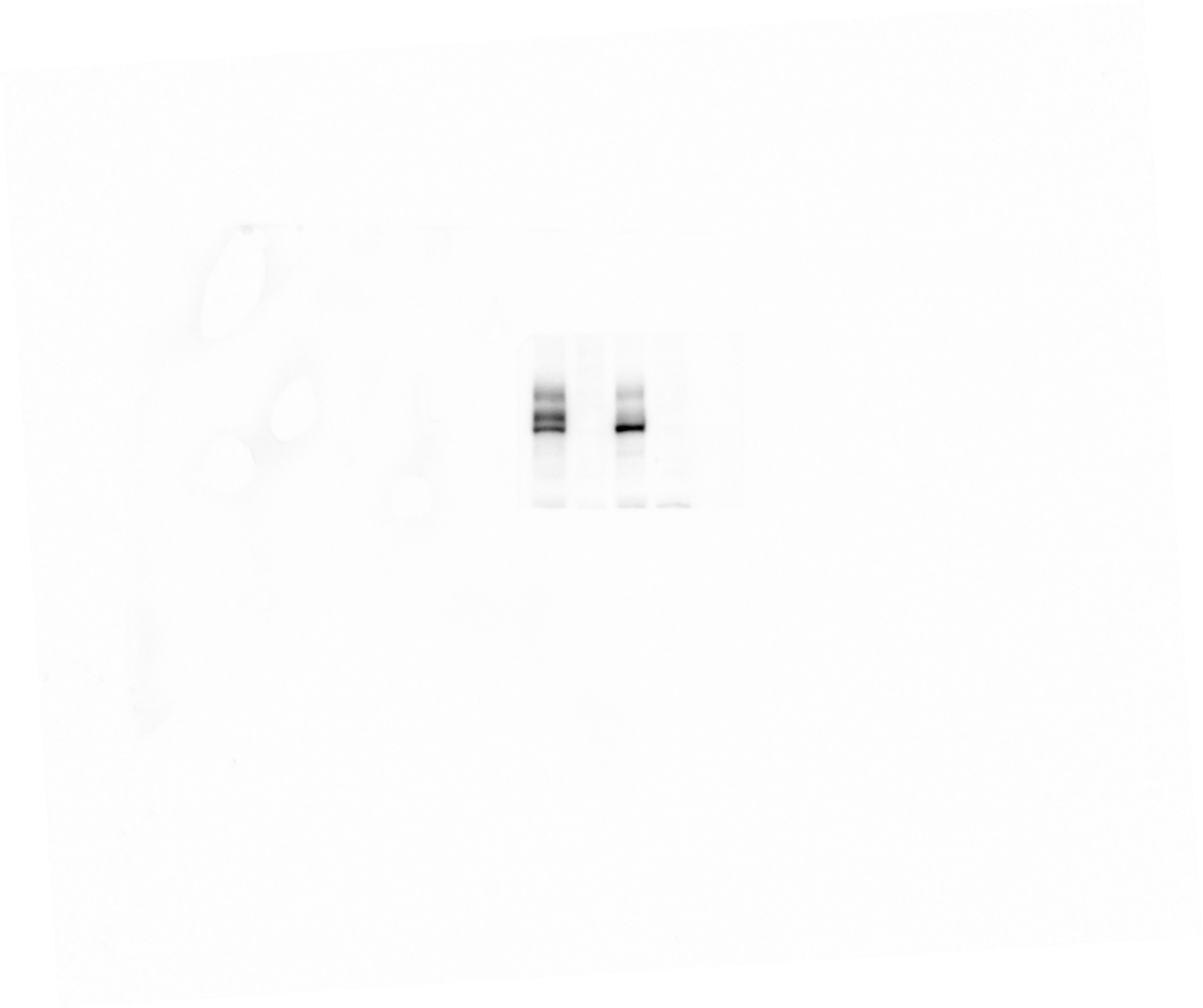

Supplement: Figure 3—source data 1. [file elife-103996-fig3-data1.zip › elife-103996-fig3-data1-v1/Figure 3A/Figure 3A DVL2.tif]

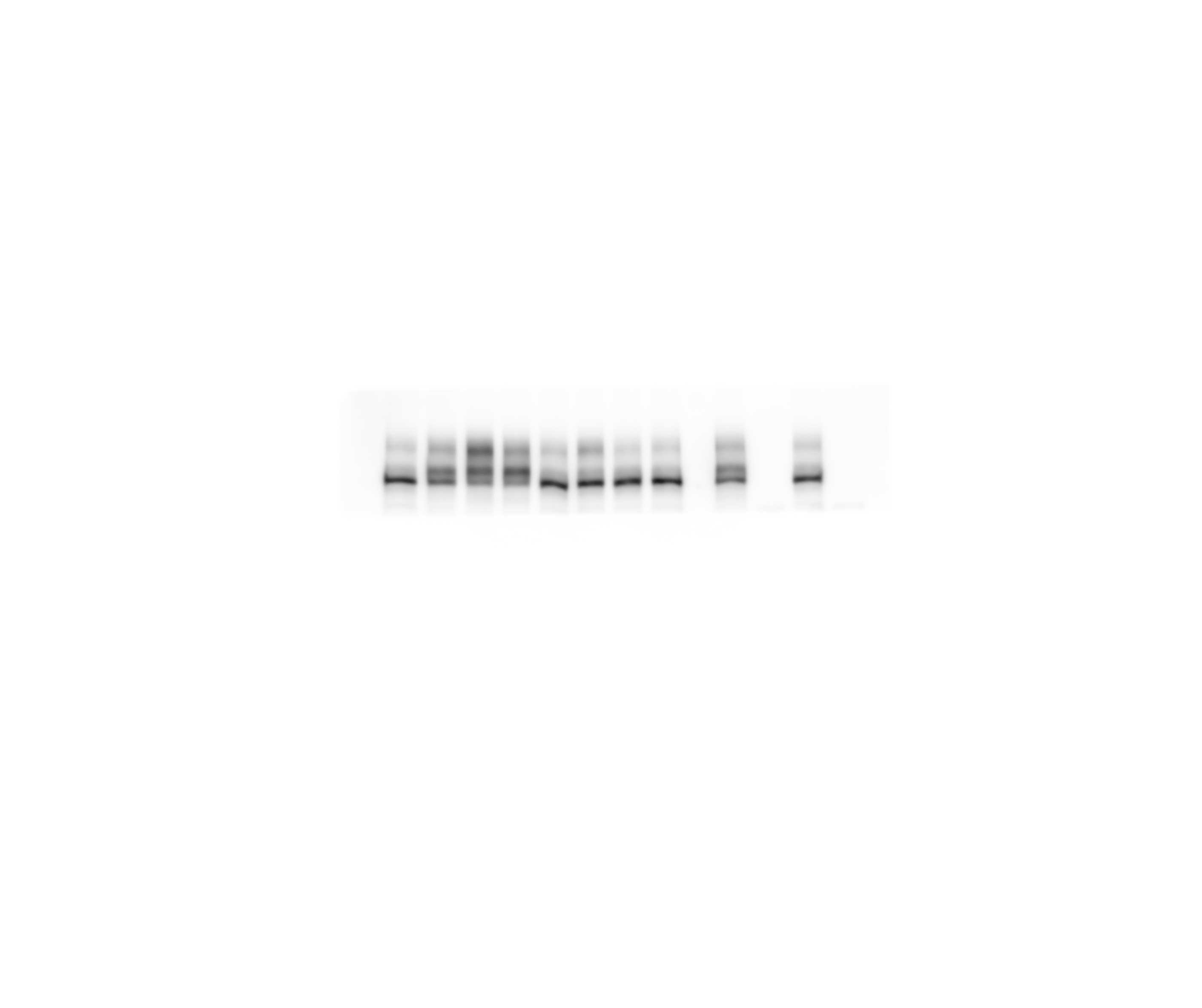

Supplement: Figure 3—source data 1. [file elife-103996-fig3-data1.zip › elife-103996-fig3-data1-v1/Figure 3A/Figure 3A DVL3.tif]

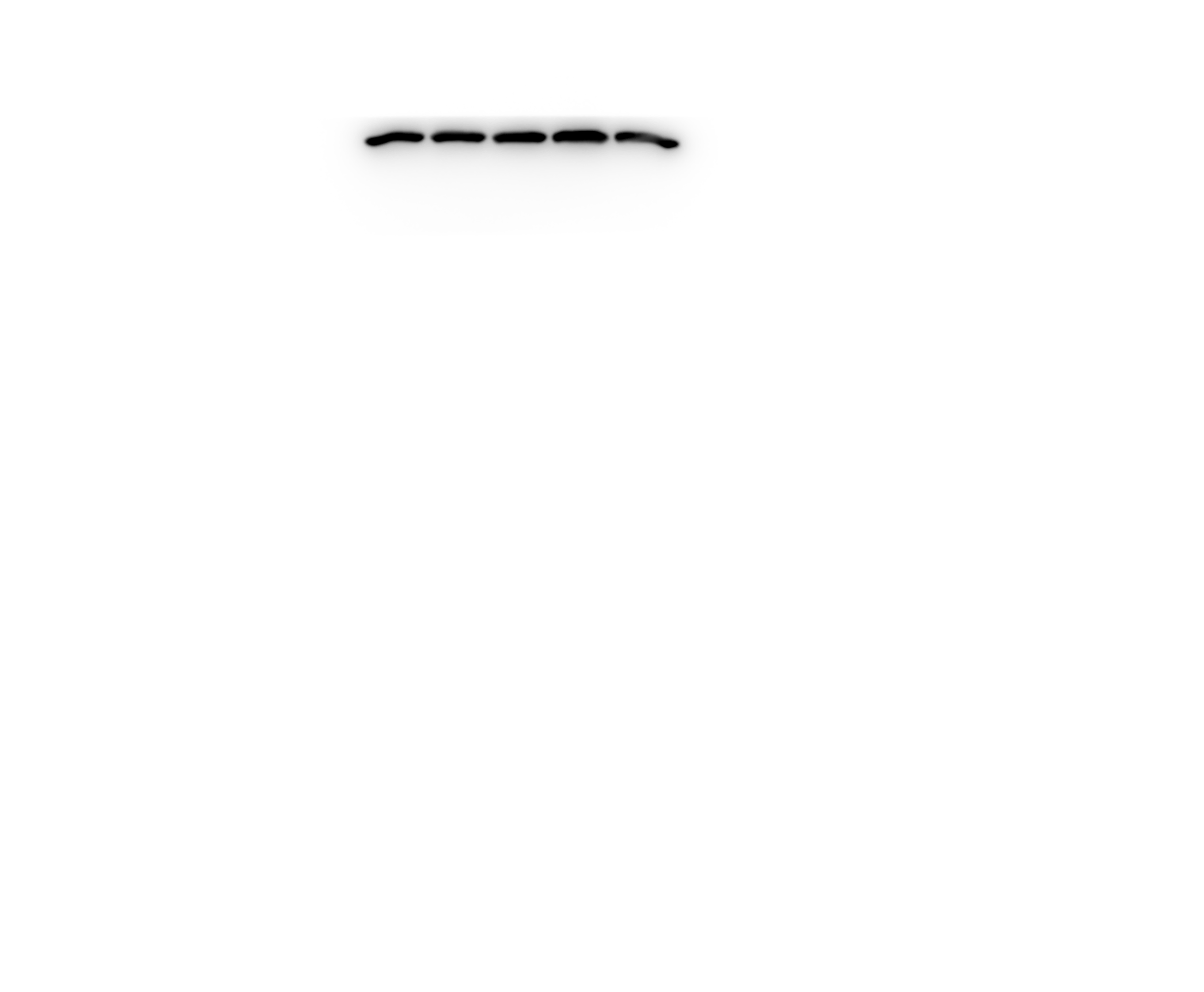

Supplement: Figure 3—source data 1. [file elife-103996-fig3-data1.zip › elife-103996-fig3-data1-v1/Figure 3F/Figure 3F Actin.tif]

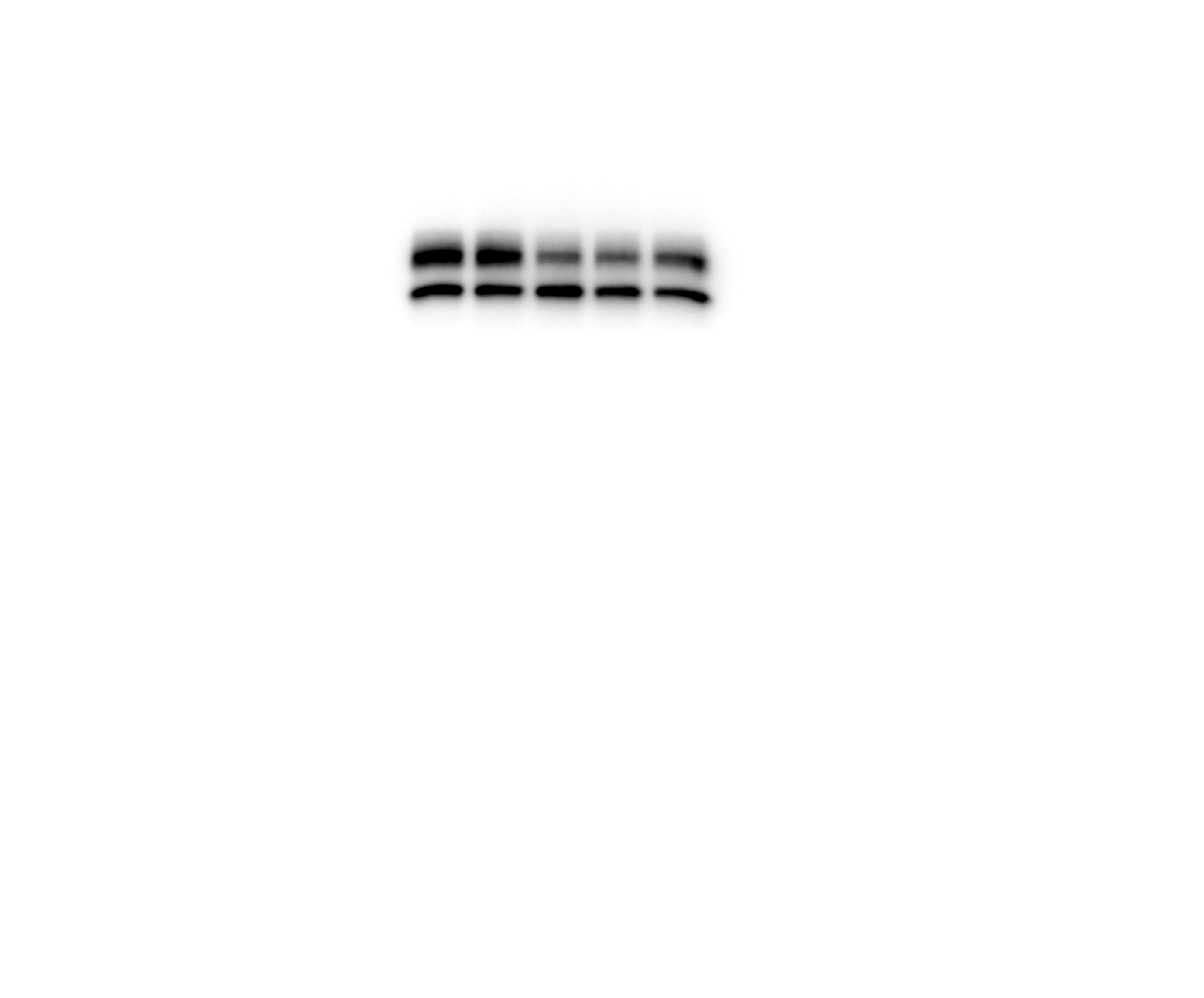

Supplement: Figure 3—source data 1. [file elife-103996-fig3-data1.zip › elife-103996-fig3-data1-v1/Figure 3F/Figure 3F DVLTKO V5-FZD5 V5.tif]

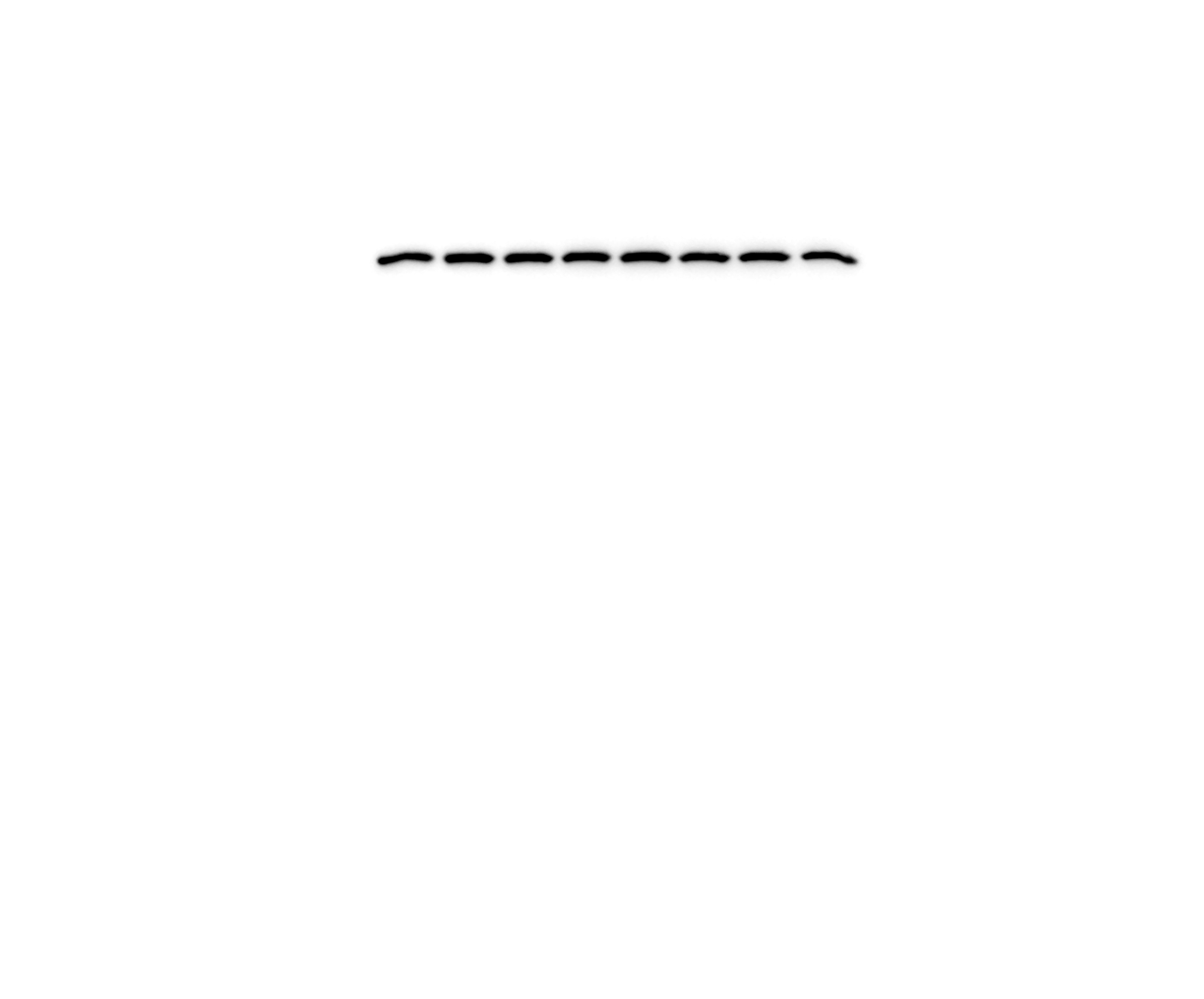

Supplement: Figure 3—source data 1. [file elife-103996-fig3-data1.zip › elife-103996-fig3-data1-v1/Figure 3K/Figure 3K Actin.tif]

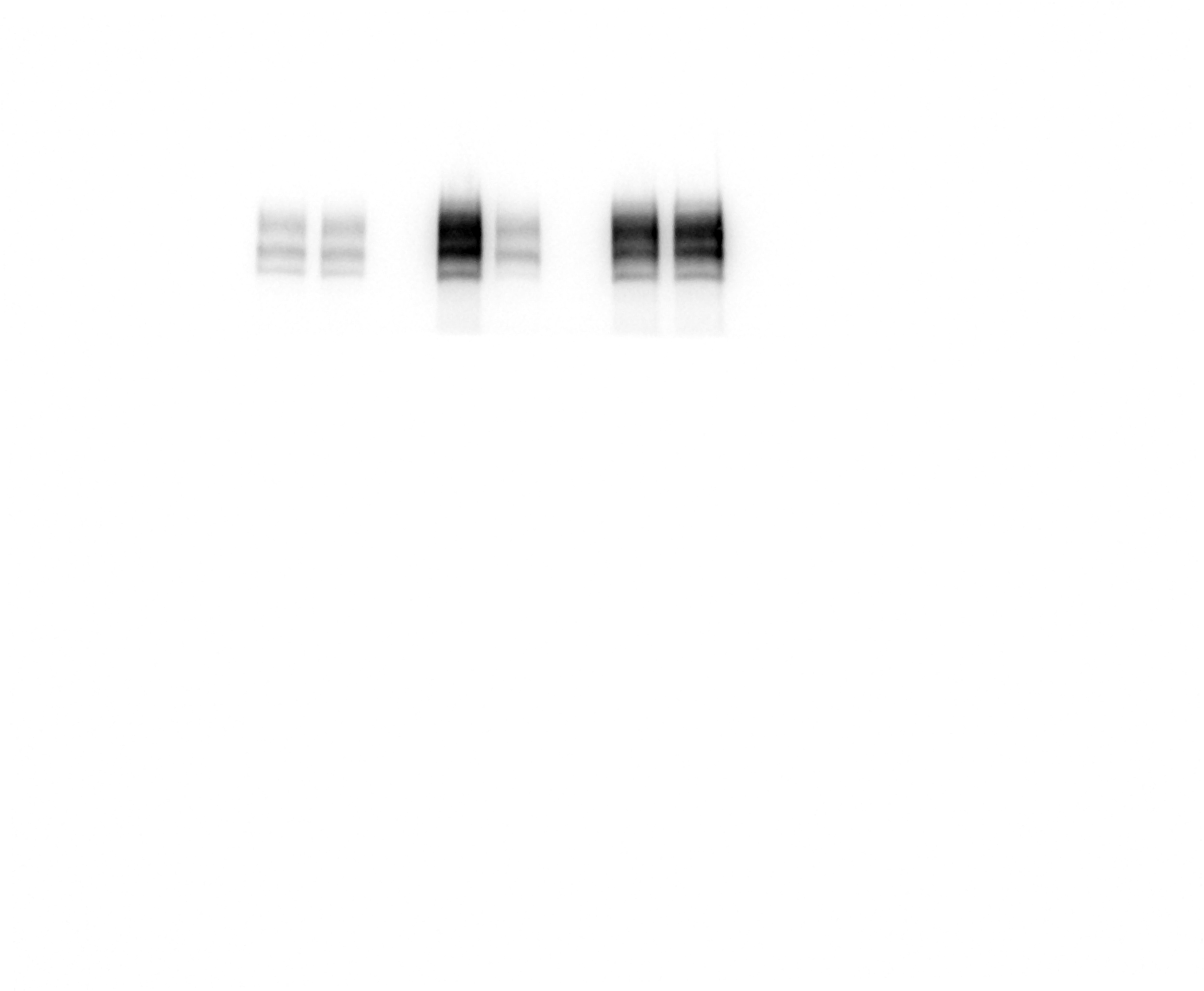

Supplement: Figure 3—source data 1. [file elife-103996-fig3-data1.zip › elife-103996-fig3-data1-v1/Figure 3K/Figure 3K DVL2.tif]

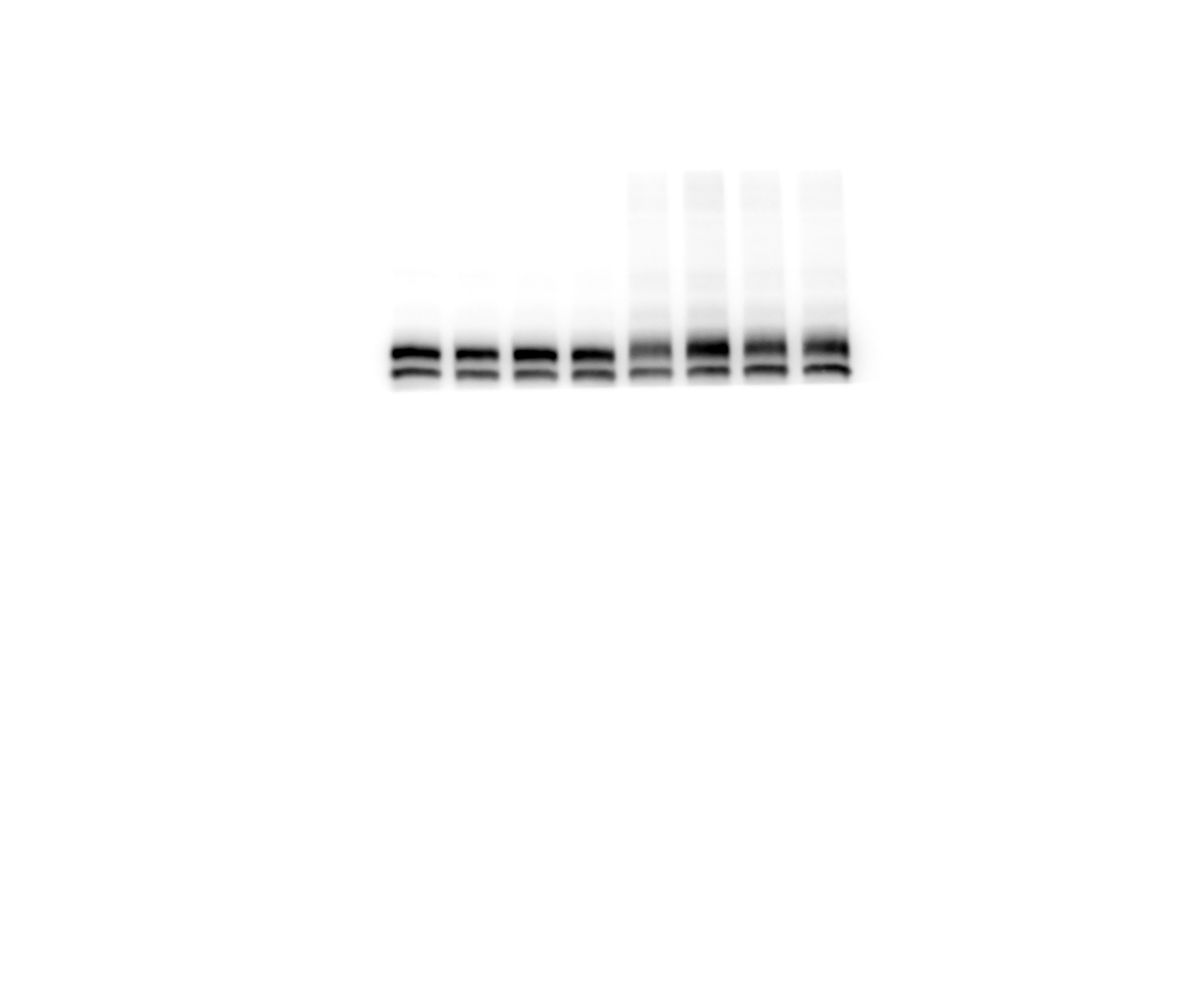

Supplement: Figure 3—source data 1. [file elife-103996-fig3-data1.zip › elife-103996-fig3-data1-v1/Figure 3K/Figure 3K V5.tif]

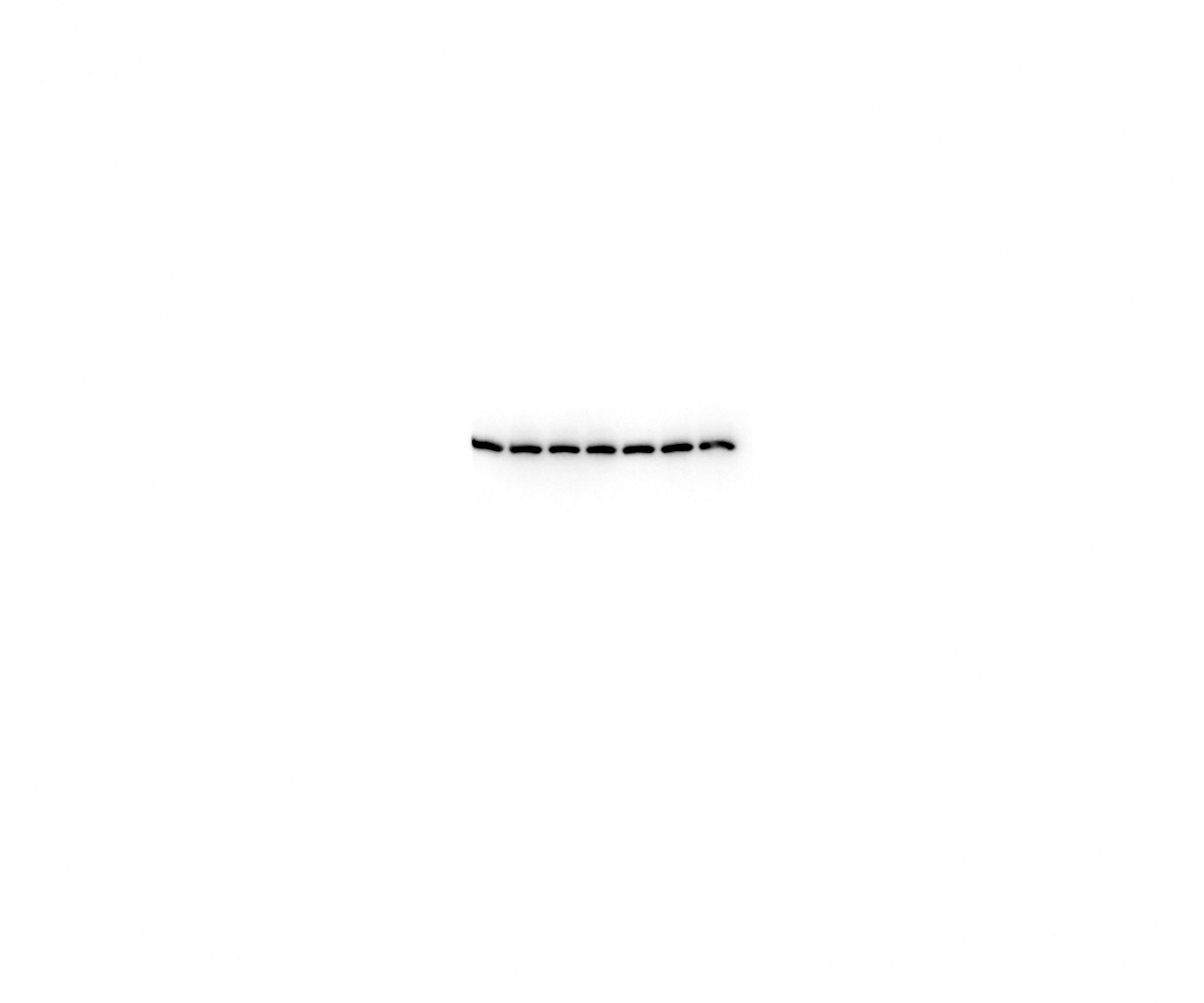

Supplement: Figure 4—source data 1. [file elife-103996-fig4-data1.zip › elife-103996-fig4-data1-v1/Figure 4B Actin.tif]

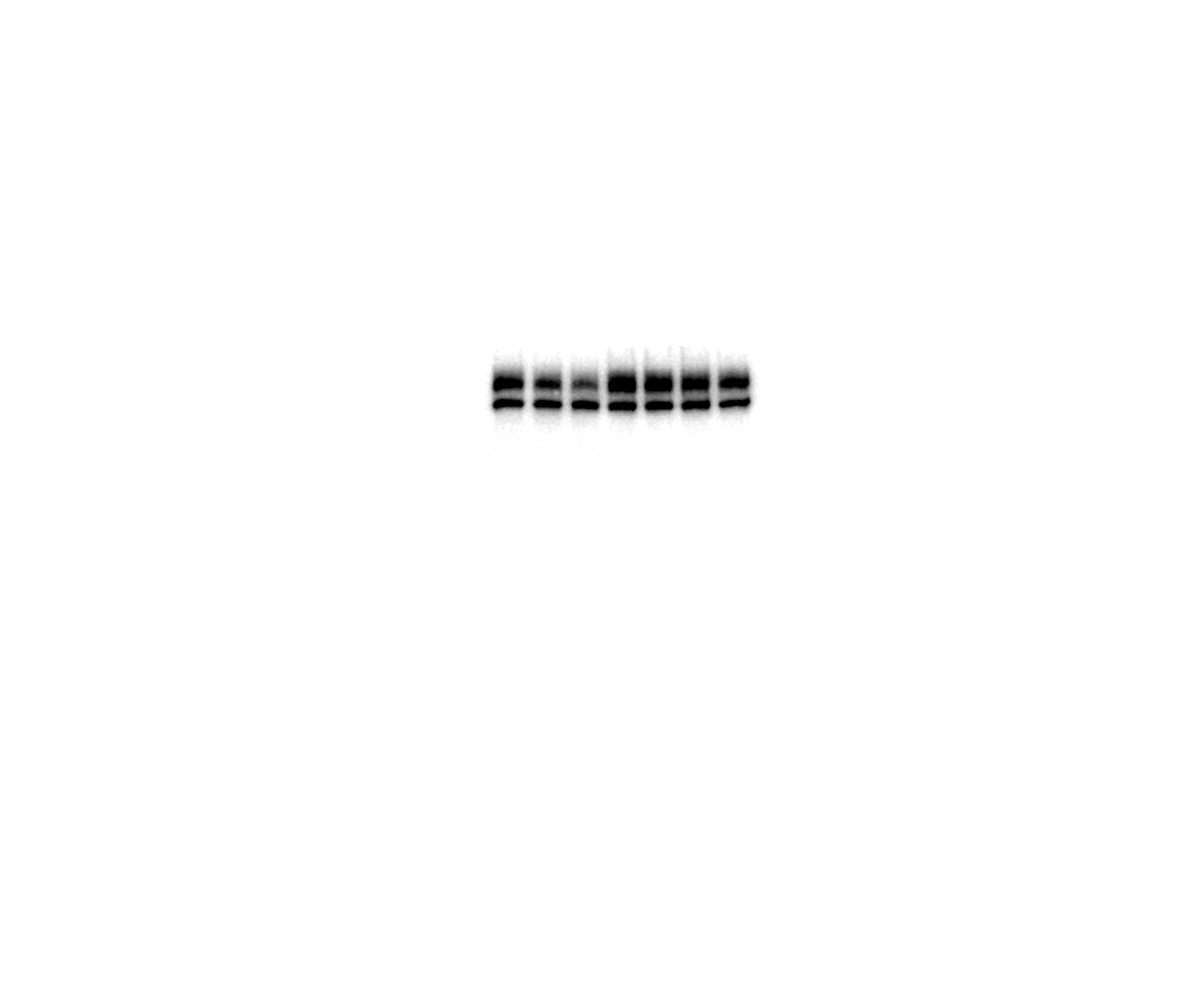

Supplement: Figure 4—source data 1. [file elife-103996-fig4-data1.zip › elife-103996-fig4-data1-v1/Figure 4B V5.tif]

## Figure 4-source data

Figure 4B:  
V5

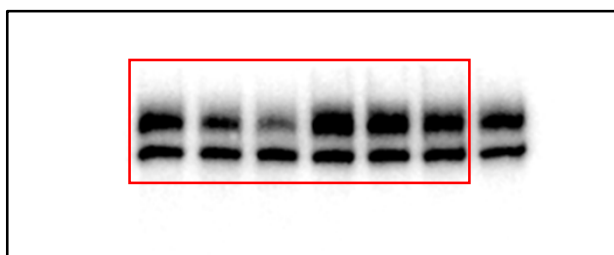

Figure 4B:  
Actin

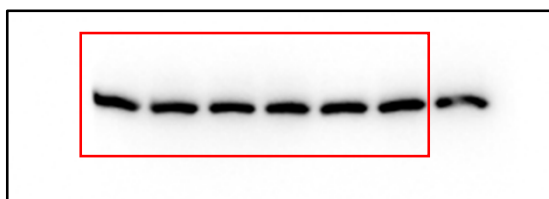

Supplement: Figure 4—source data 2. [file elife-103996-fig4-data2.zip › elife-103996-fig4-data2-v1.pdf]

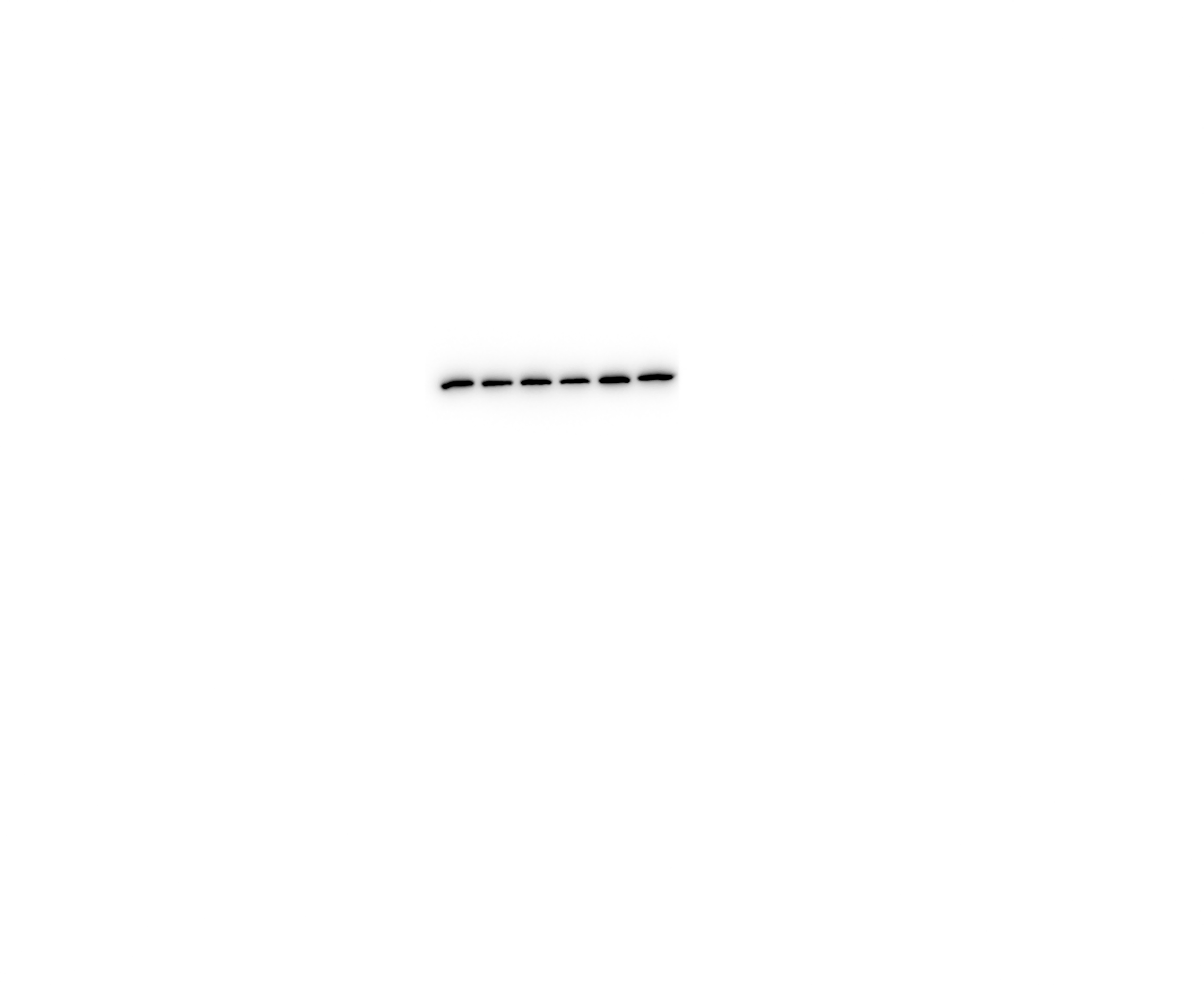

Supplement: Figure 5—source data 1. [file elife-103996-fig5-data1.zip › elife-103996-fig5-data1-v1/Figure 5B/Figure 5B Actin.tif]

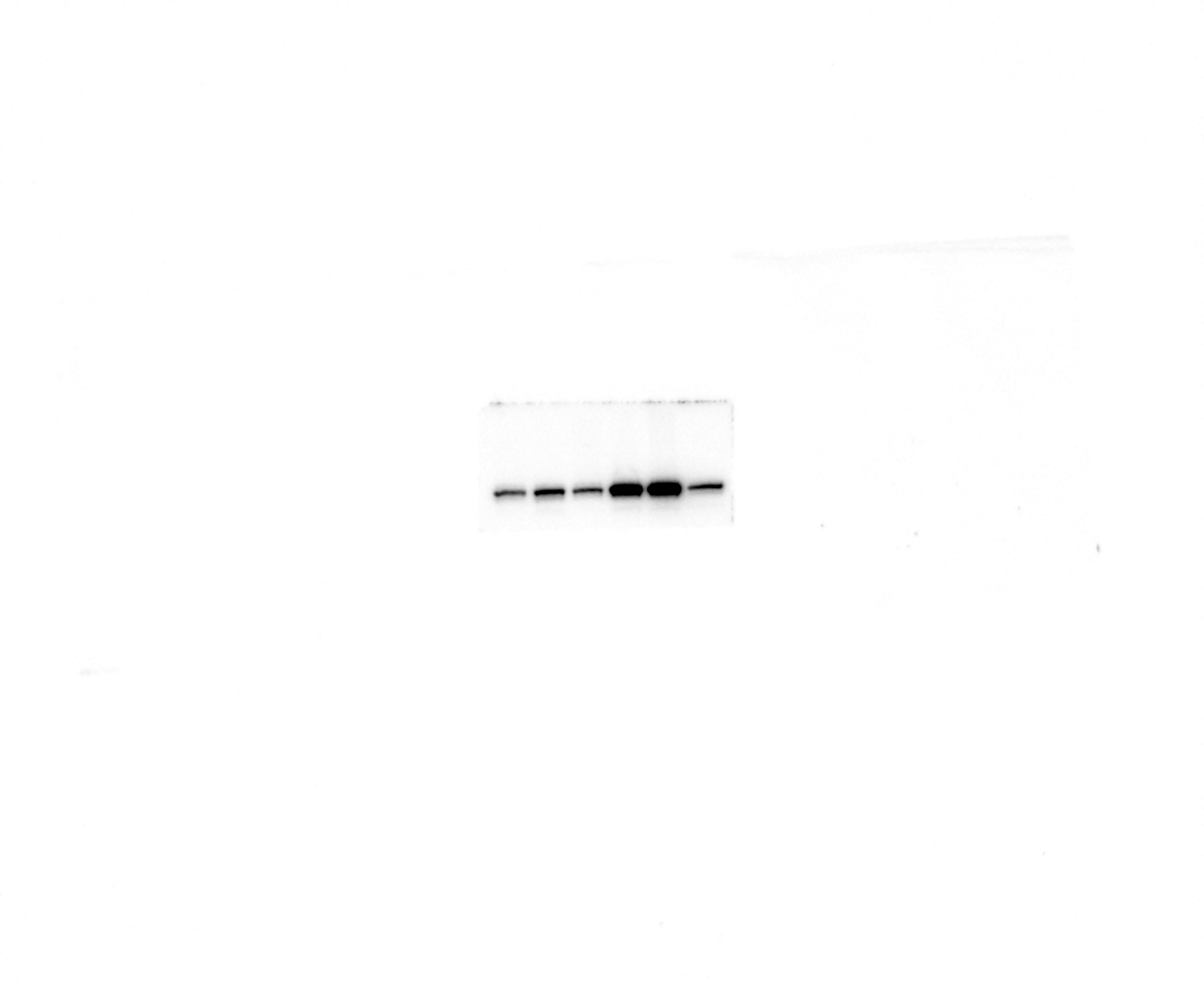

Supplement: Figure 5—source data 1. [file elife-103996-fig5-data1.zip › elife-103996-fig5-data1-v1/Figure 5B/Figure 5B b-cat.tif]

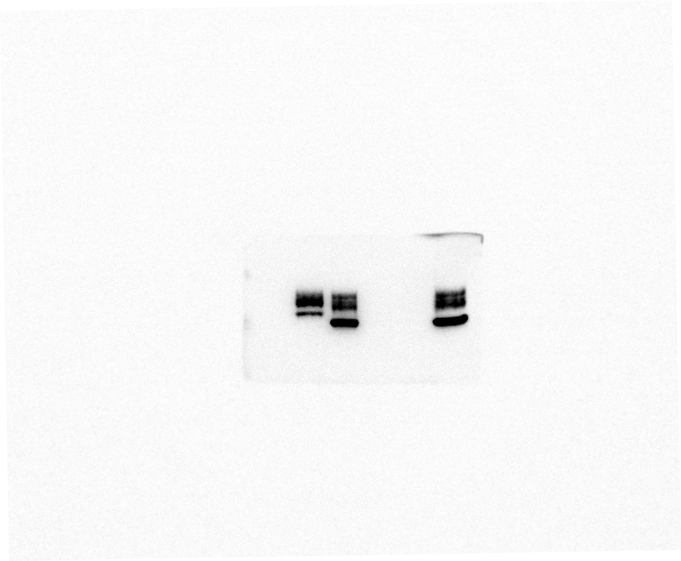

Supplement: Figure 5—source data 1. [file elife-103996-fig5-data1.zip › elife-103996-fig5-data1-v1/Figure 5B/Figure 5B V5.tif]

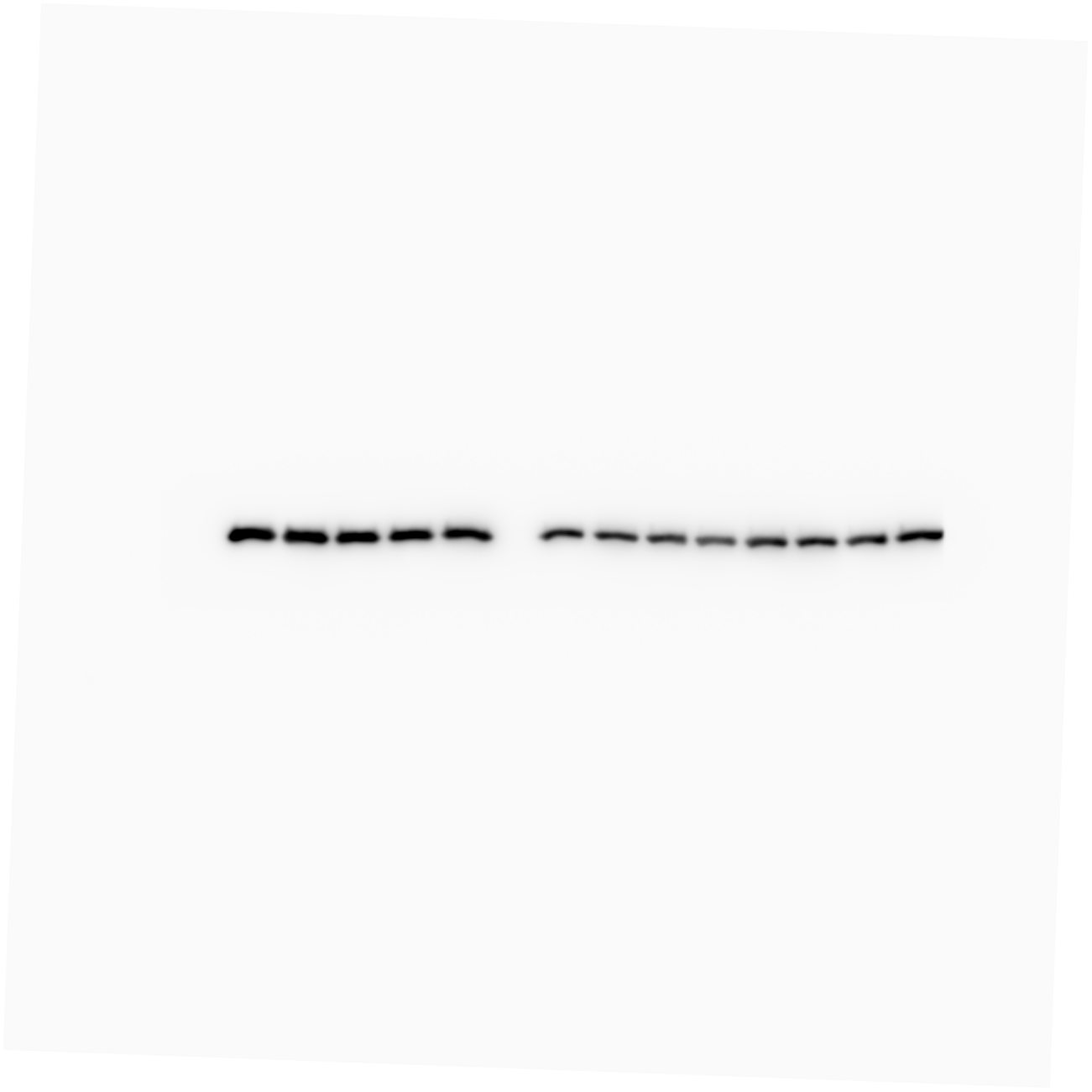

Supplement: Figure 5—source data 1. [file elife-103996-fig5-data1.zip › elife-103996-fig5-data1-v1/Figure 5C/Figure 5C Actin.tif]

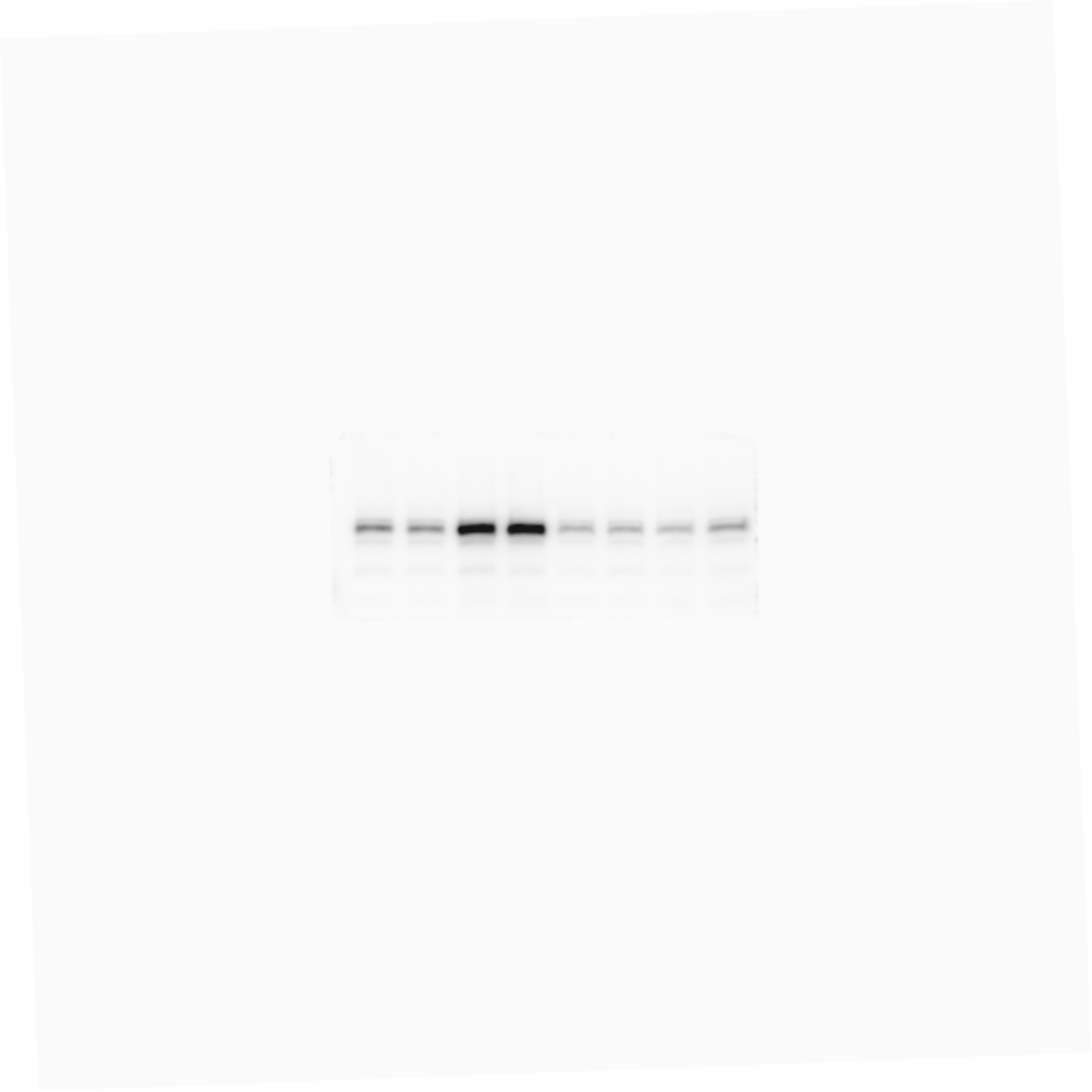

Supplement: Figure 5—source data 1. [file elife-103996-fig5-data1.zip › elife-103996-fig5-data1-v1/Figure 5C/Figure 5C b-cat.tif]

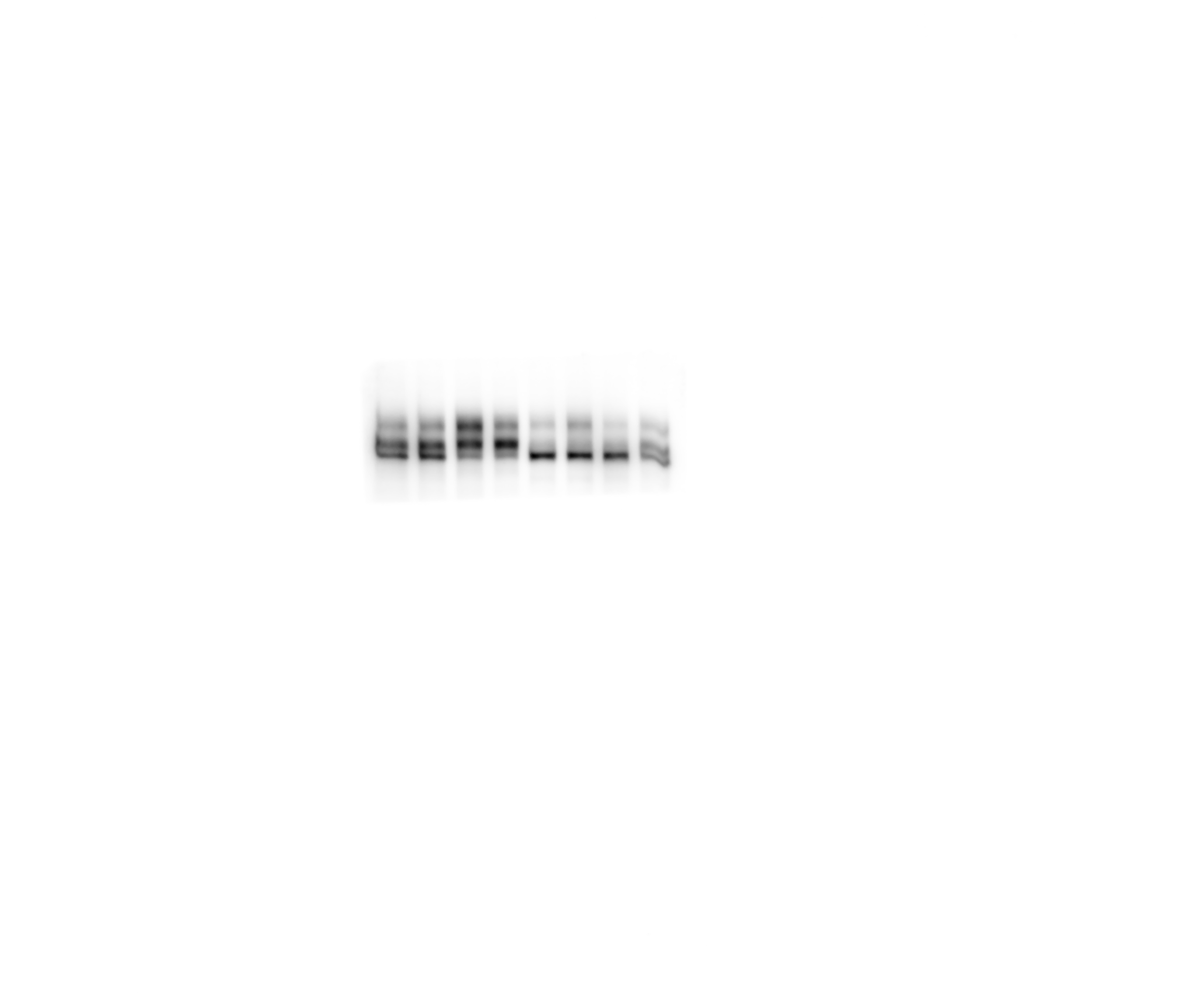

Supplement: Figure 5—source data 1. [file elife-103996-fig5-data1.zip › elife-103996-fig5-data1-v1/Figure 5C/Figure 5C DVL2.tif]

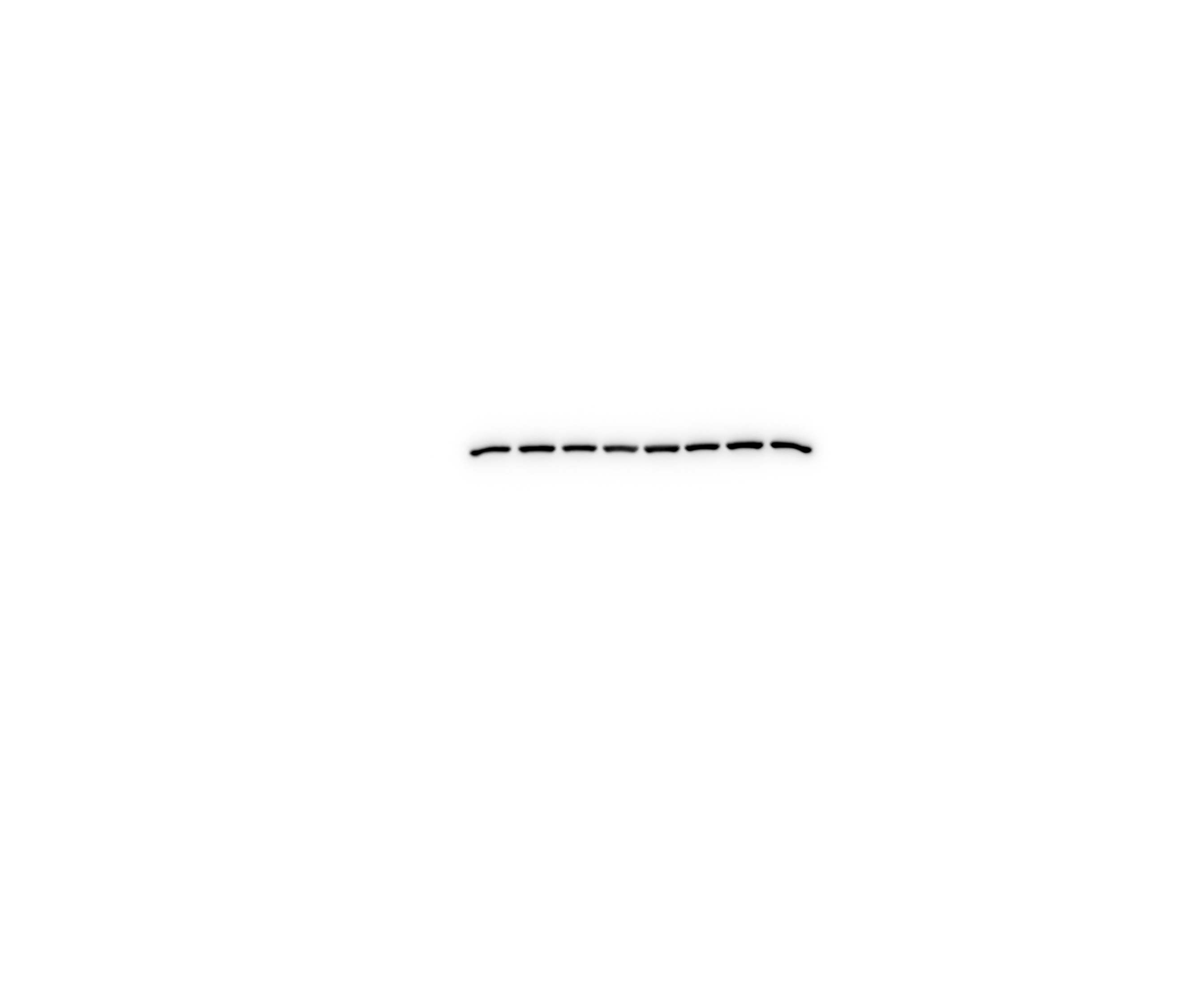

Supplement: Figure 5—source data 1. [file elife-103996-fig5-data1.zip › elife-103996-fig5-data1-v1/Figure 5D/Figure 5D Actin.tif]

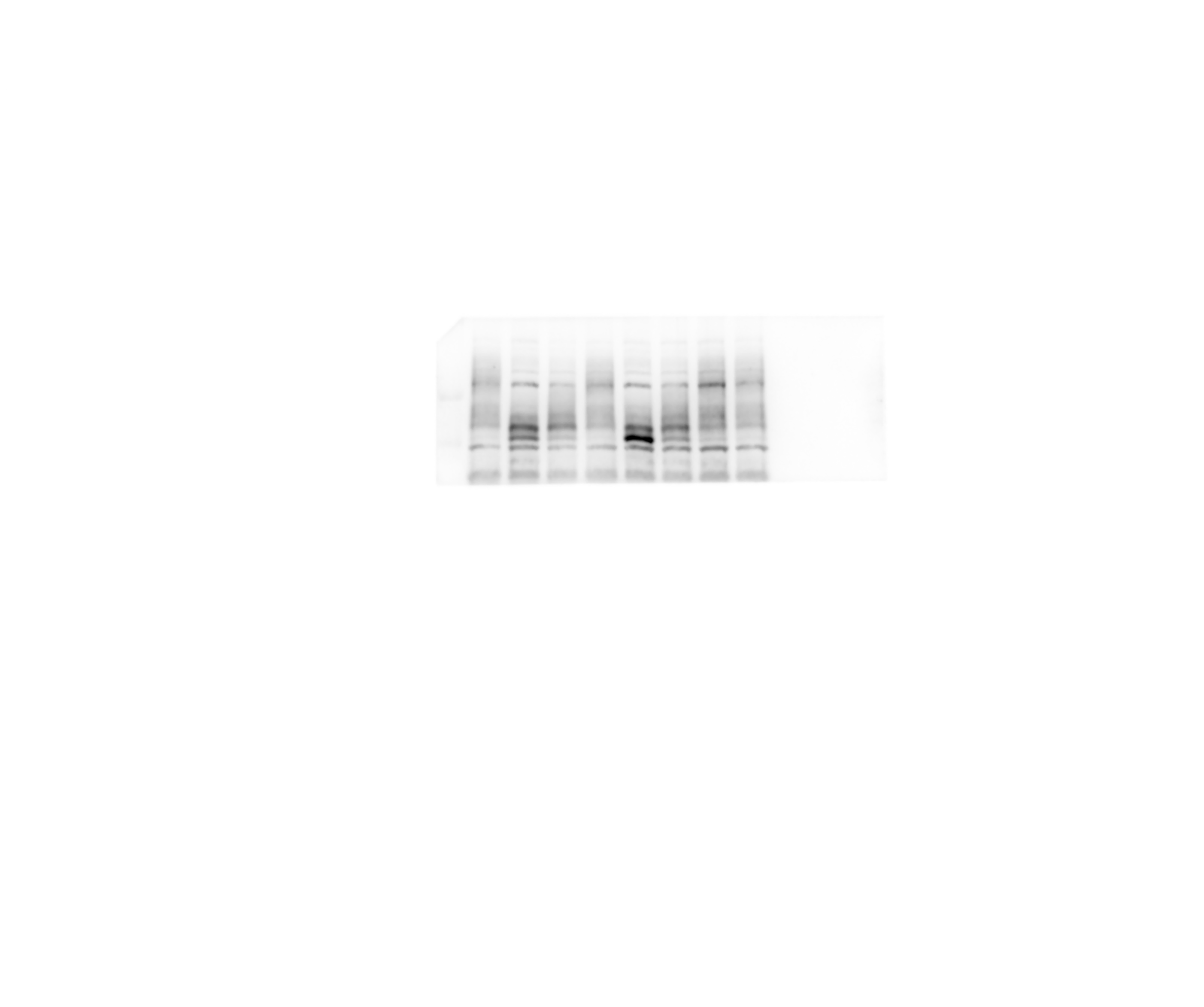

Supplement: Figure 5—source data 1. [file elife-103996-fig5-data1.zip › elife-103996-fig5-data1-v1/Figure 5D/Figure 5D DVL1.tif]

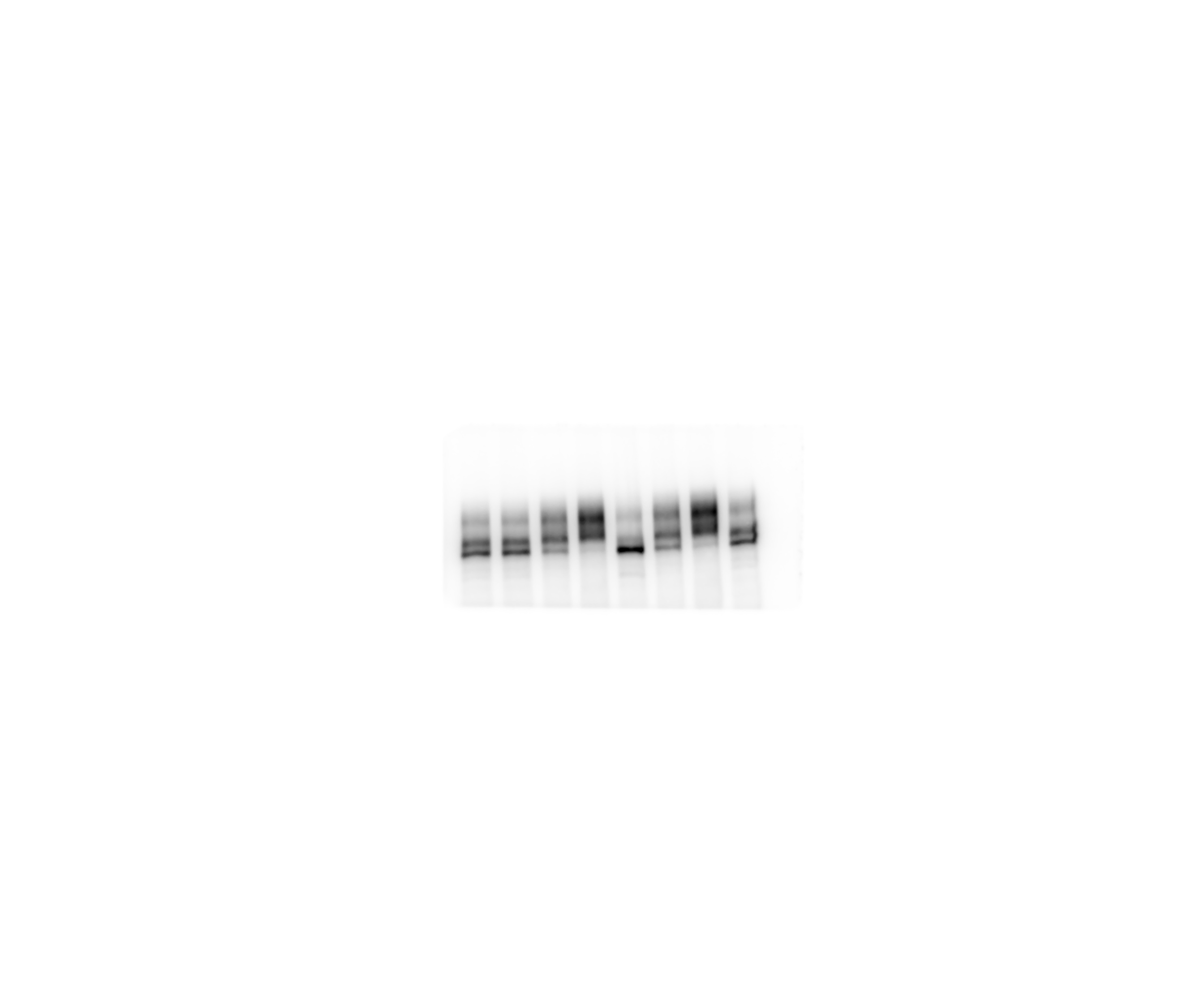

Supplement: Figure 5—source data 1. [file elife-103996-fig5-data1.zip › elife-103996-fig5-data1-v1/Figure 5D/Figure 5D DVL2.tif]

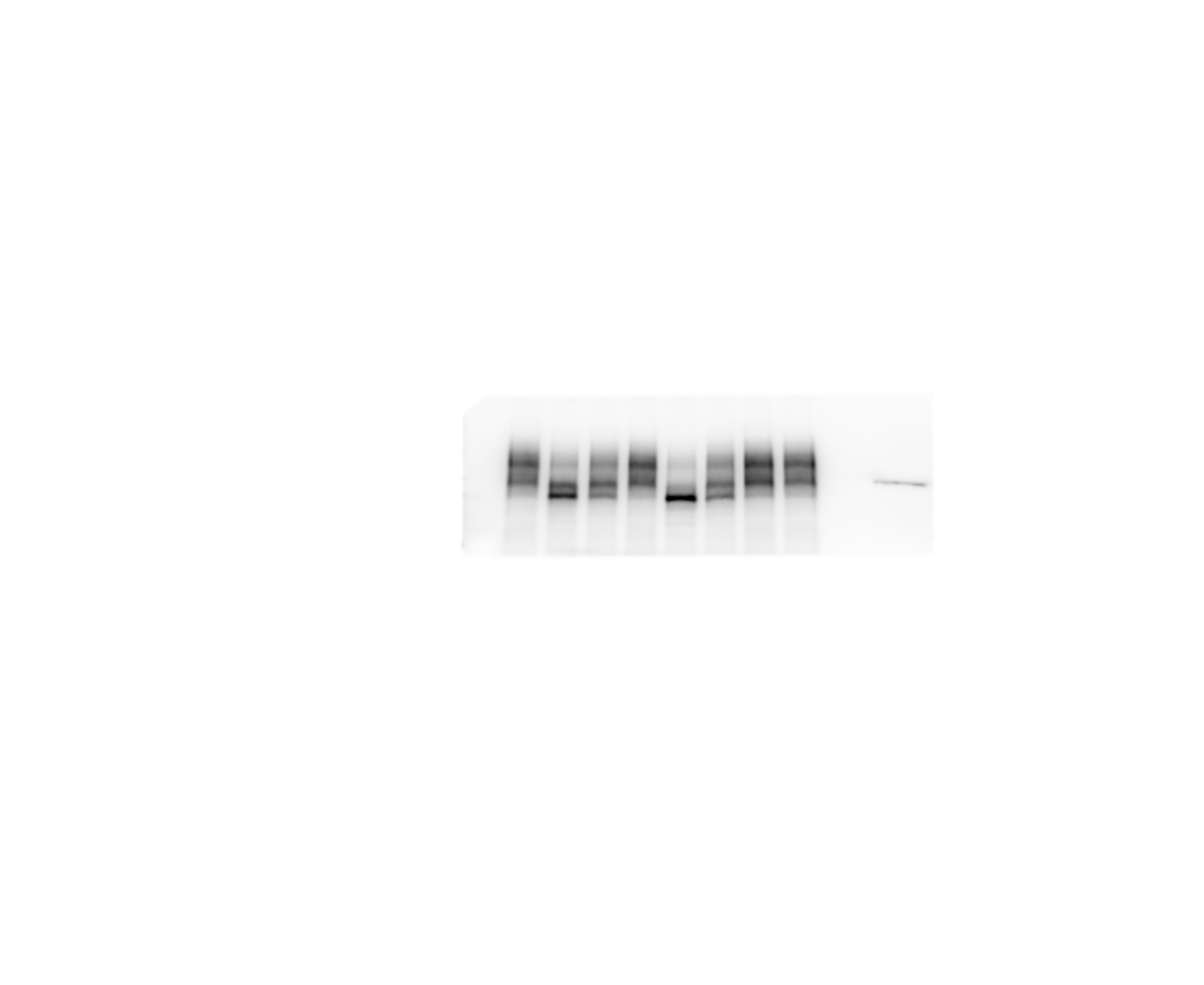

Supplement: Figure 5—source data 1. [file elife-103996-fig5-data1.zip › elife-103996-fig5-data1-v1/Figure 5D/Figure 5D DVL3.tif]

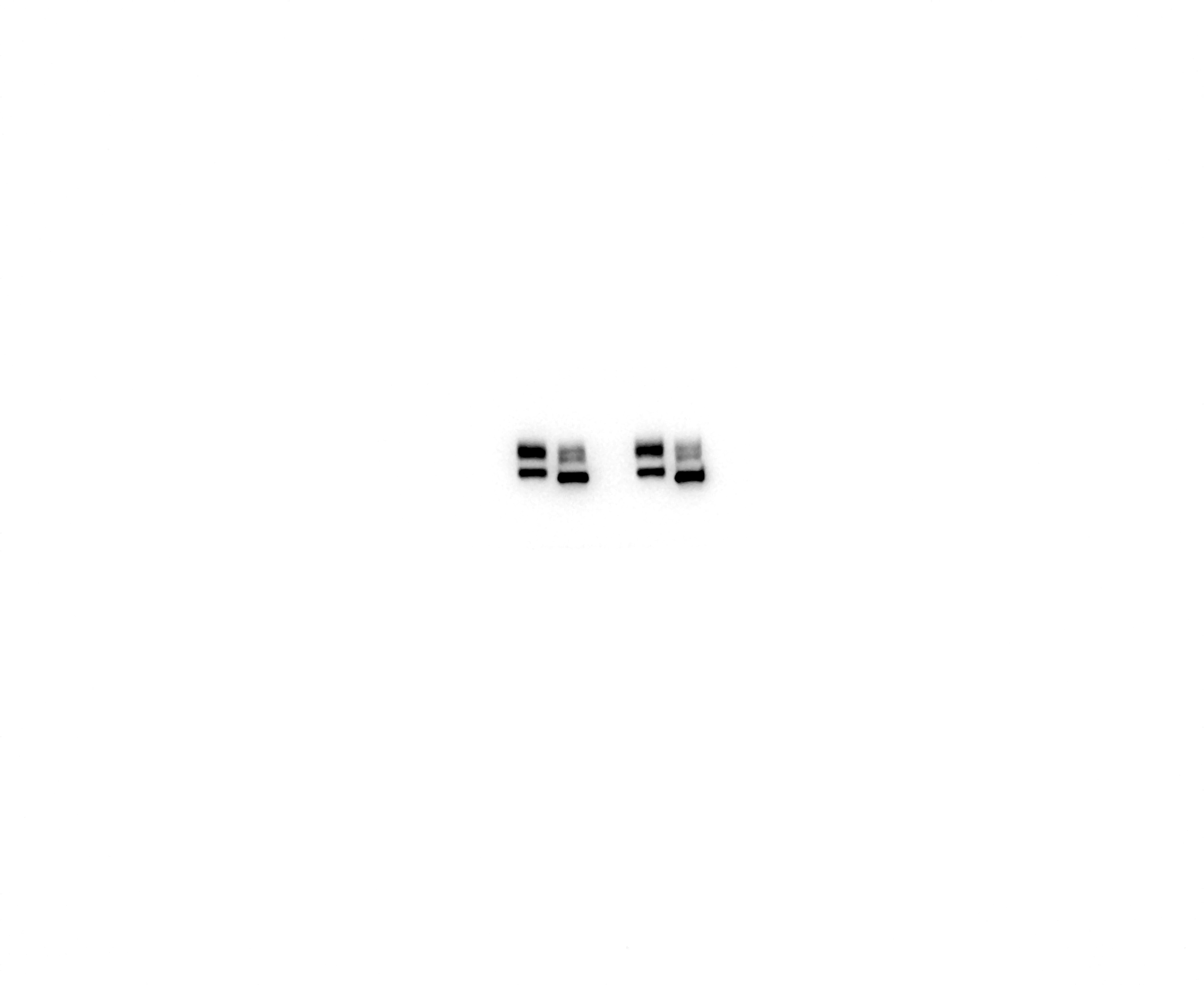

Supplement: Figure 5—source data 1. [file elife-103996-fig5-data1.zip › elife-103996-fig5-data1-v1/Figure 5D/Figure 5D V5.tif]

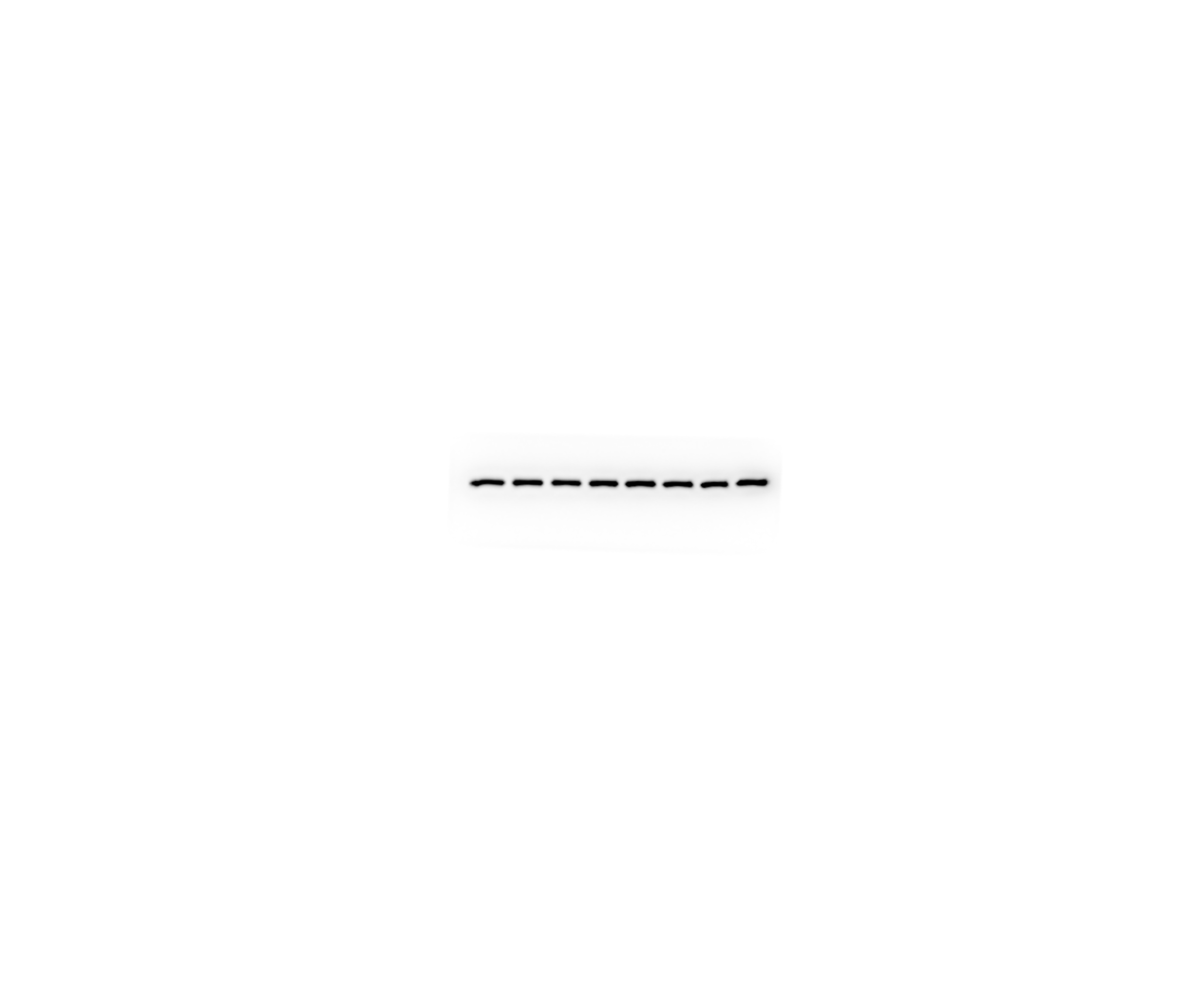

Supplement: Figure 5—source data 1. [file elife-103996-fig5-data1.zip › elife-103996-fig5-data1-v1/Figure 5E/Figure 5E Actin.tif]

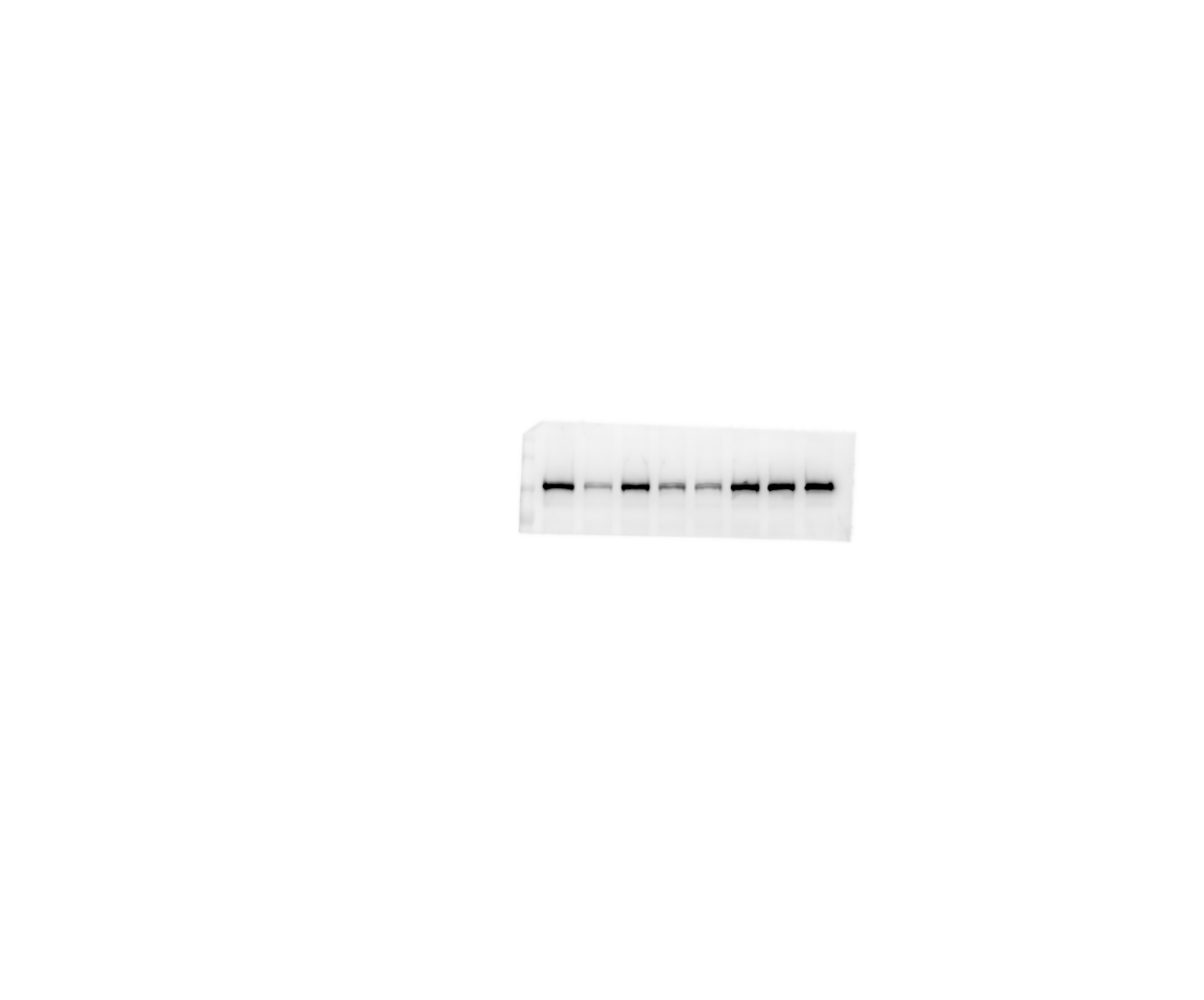

Supplement: Figure 5—source data 1. [file elife-103996-fig5-data1.zip › elife-103996-fig5-data1-v1/Figure 5E/Figure 5E b-cat.tif]

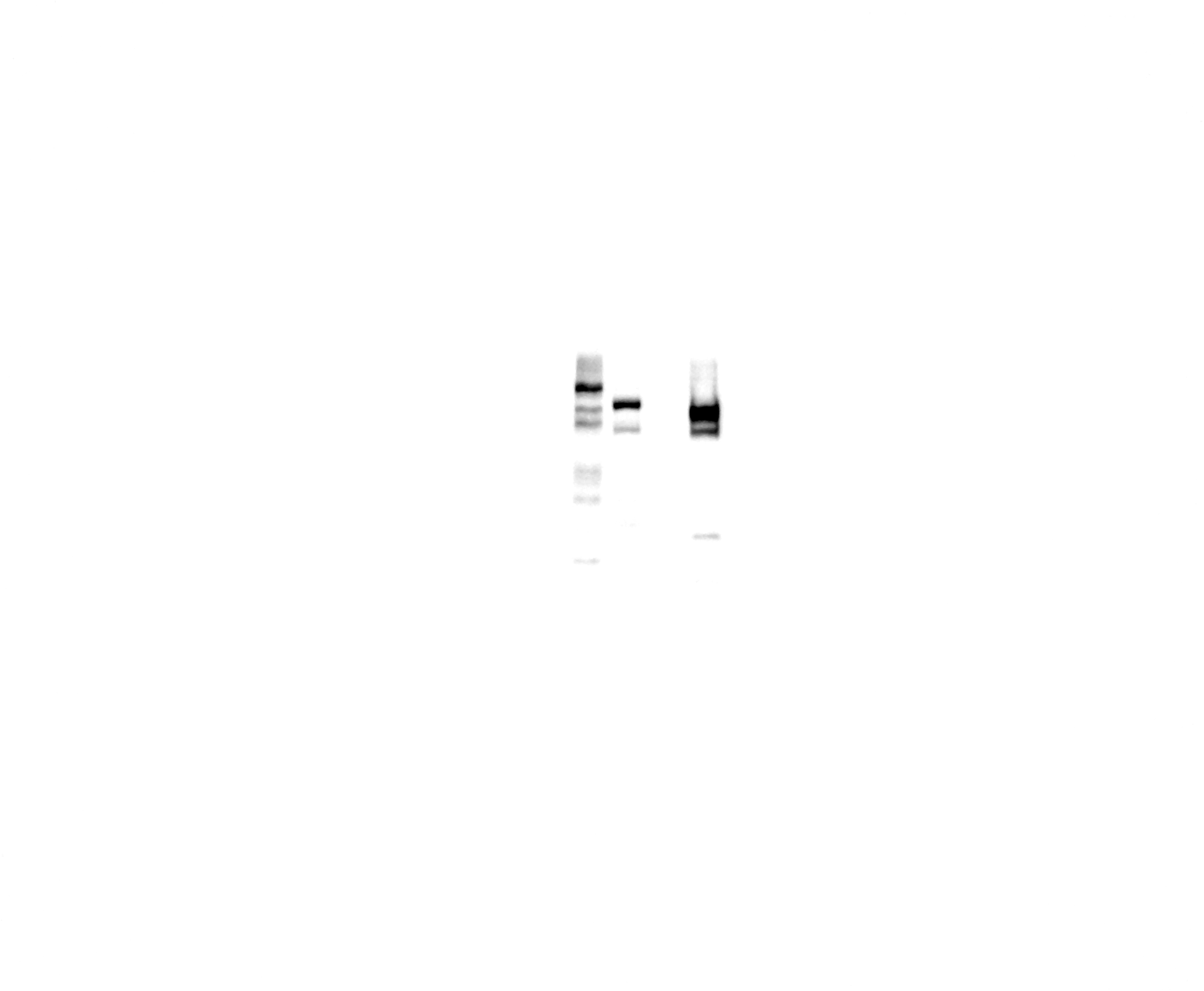

Supplement: Figure 5—source data 1. [file elife-103996-fig5-data1.zip › elife-103996-fig5-data1-v1/Figure 5E/Figure 5E HA-1.tif]

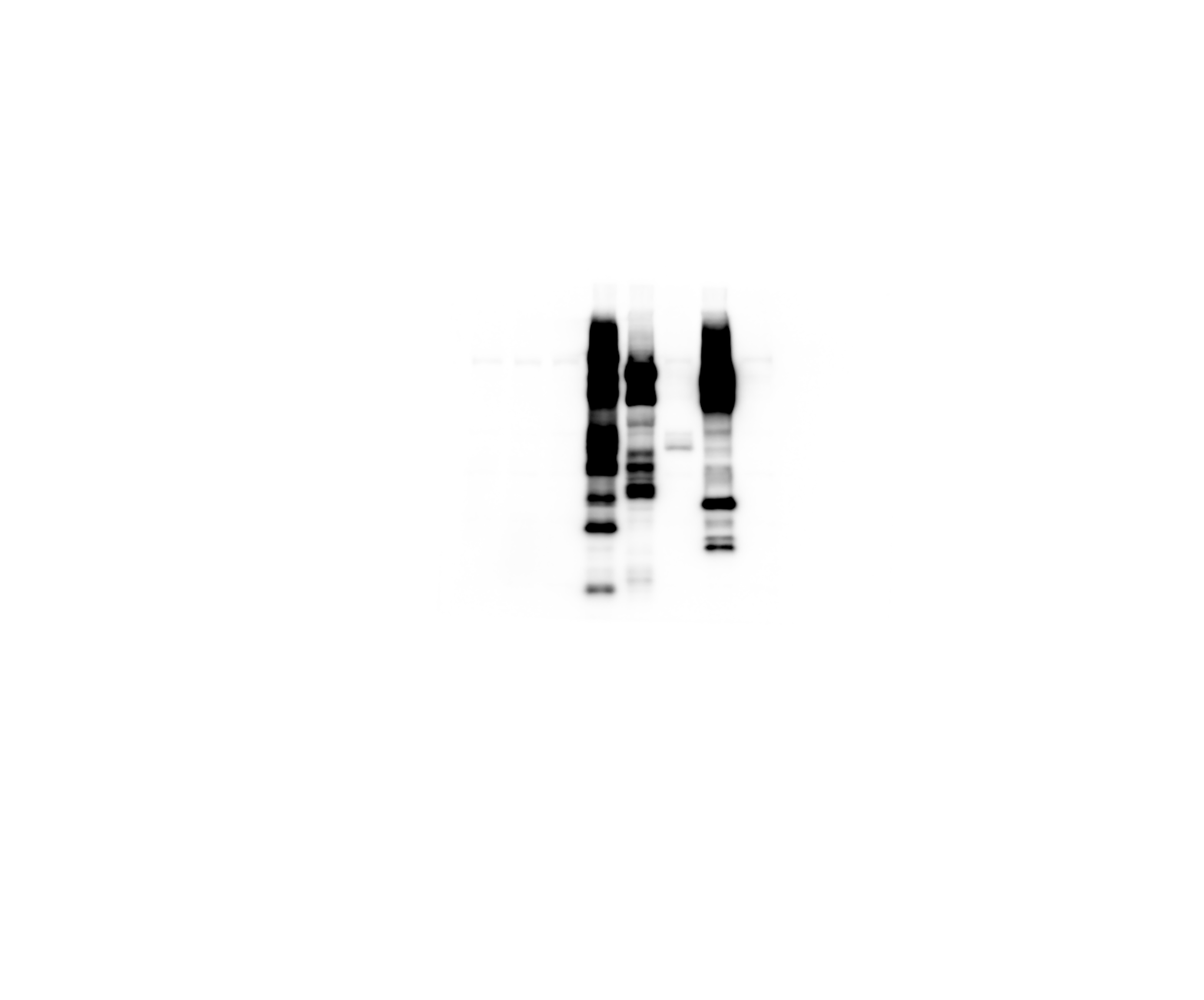

Supplement: Figure 5—source data 1. [file elife-103996-fig5-data1.zip › elife-103996-fig5-data1-v1/Figure 5E/Figure 5E HA-2.tif]

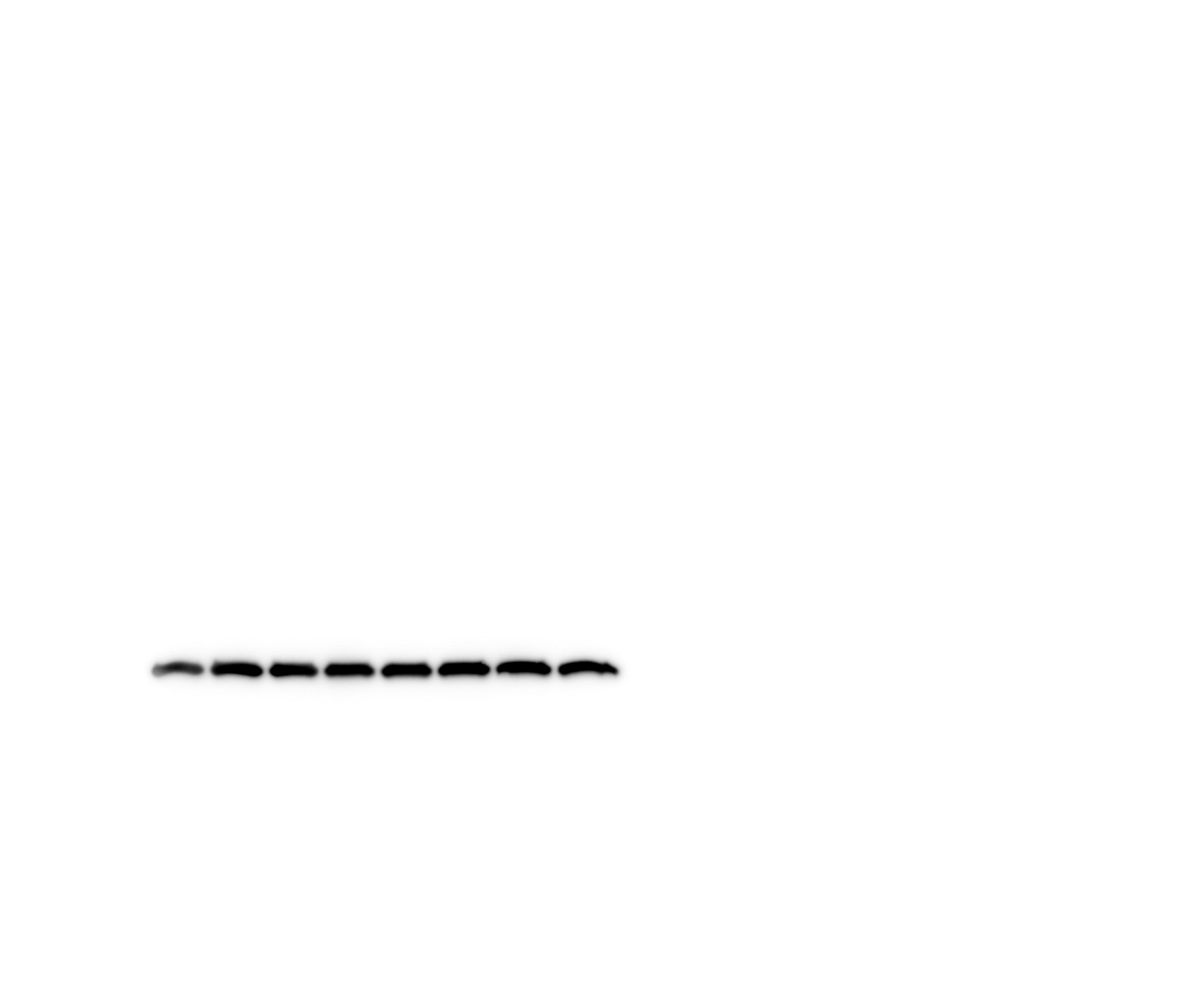

Supplement: Figure 5—source data 1. [file elife-103996-fig5-data1.zip › elife-103996-fig5-data1-v1/Figure 5F/Figure 5F Actin.tif]

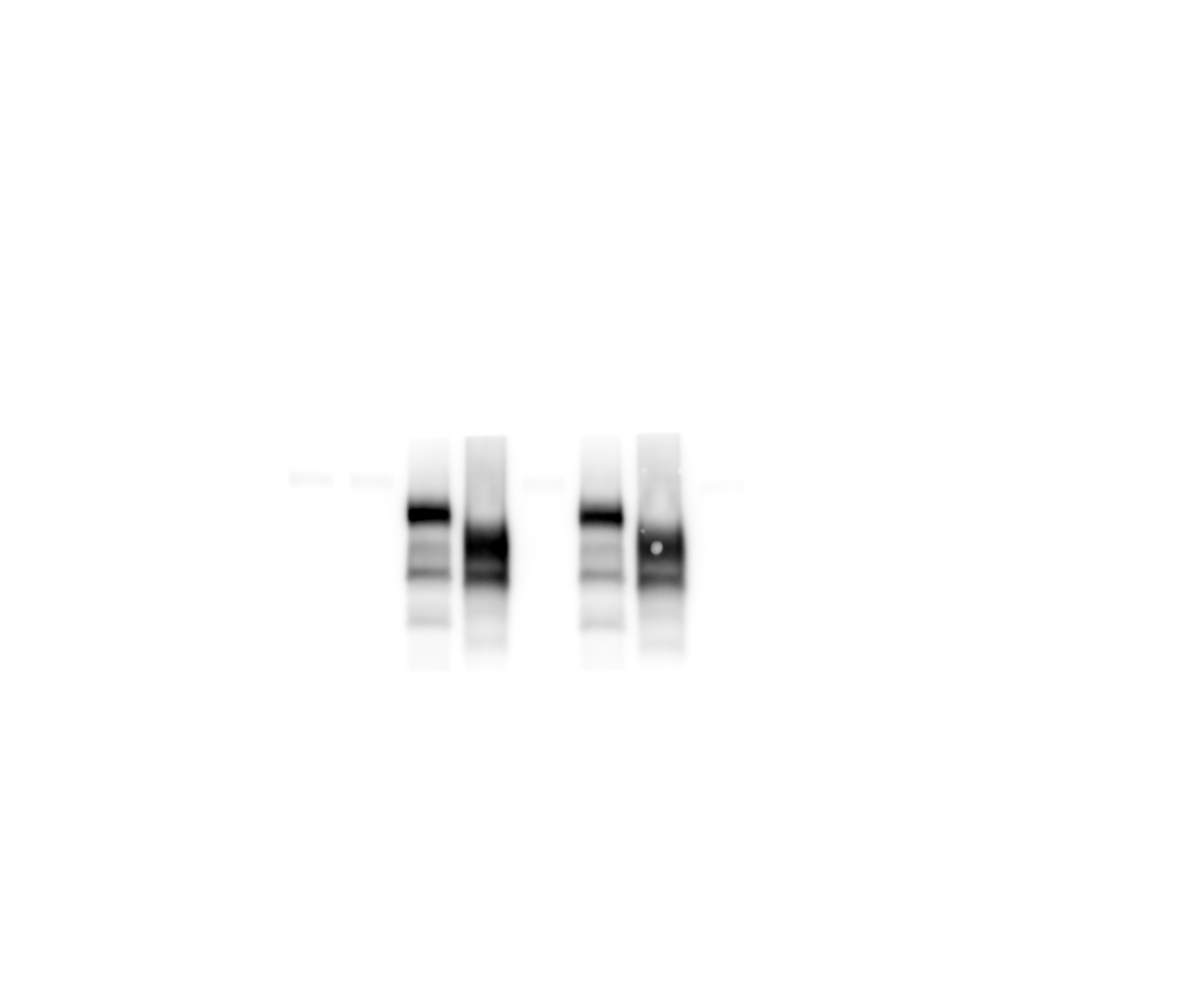

Supplement: Figure 5—source data 1. [file elife-103996-fig5-data1.zip › elife-103996-fig5-data1-v1/Figure 5F/Figure 5F HA.tif]

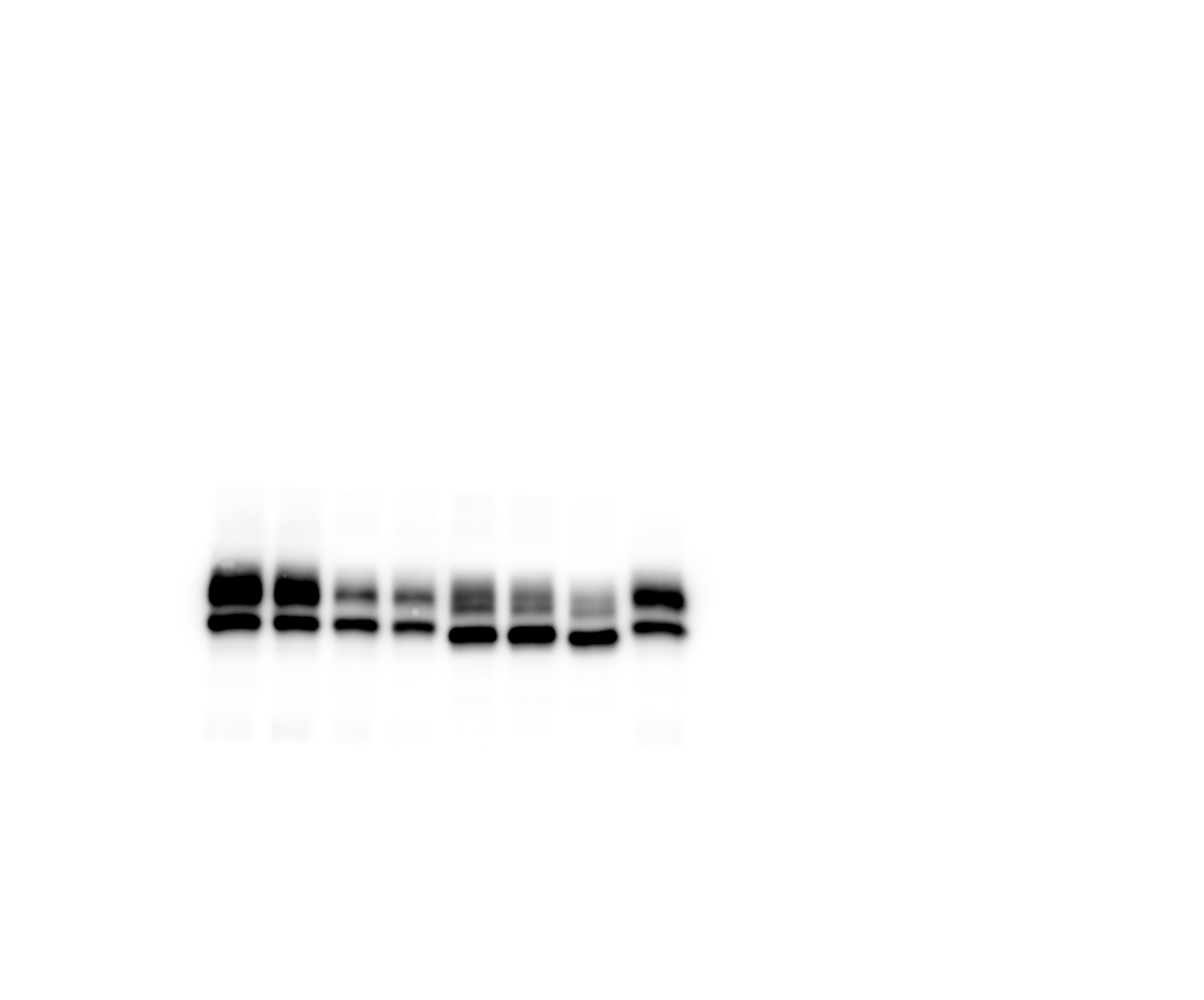

Supplement: Figure 5—source data 1. [file elife-103996-fig5-data1.zip › elife-103996-fig5-data1-v1/Figure 5F/Figure 5F V5.tif]

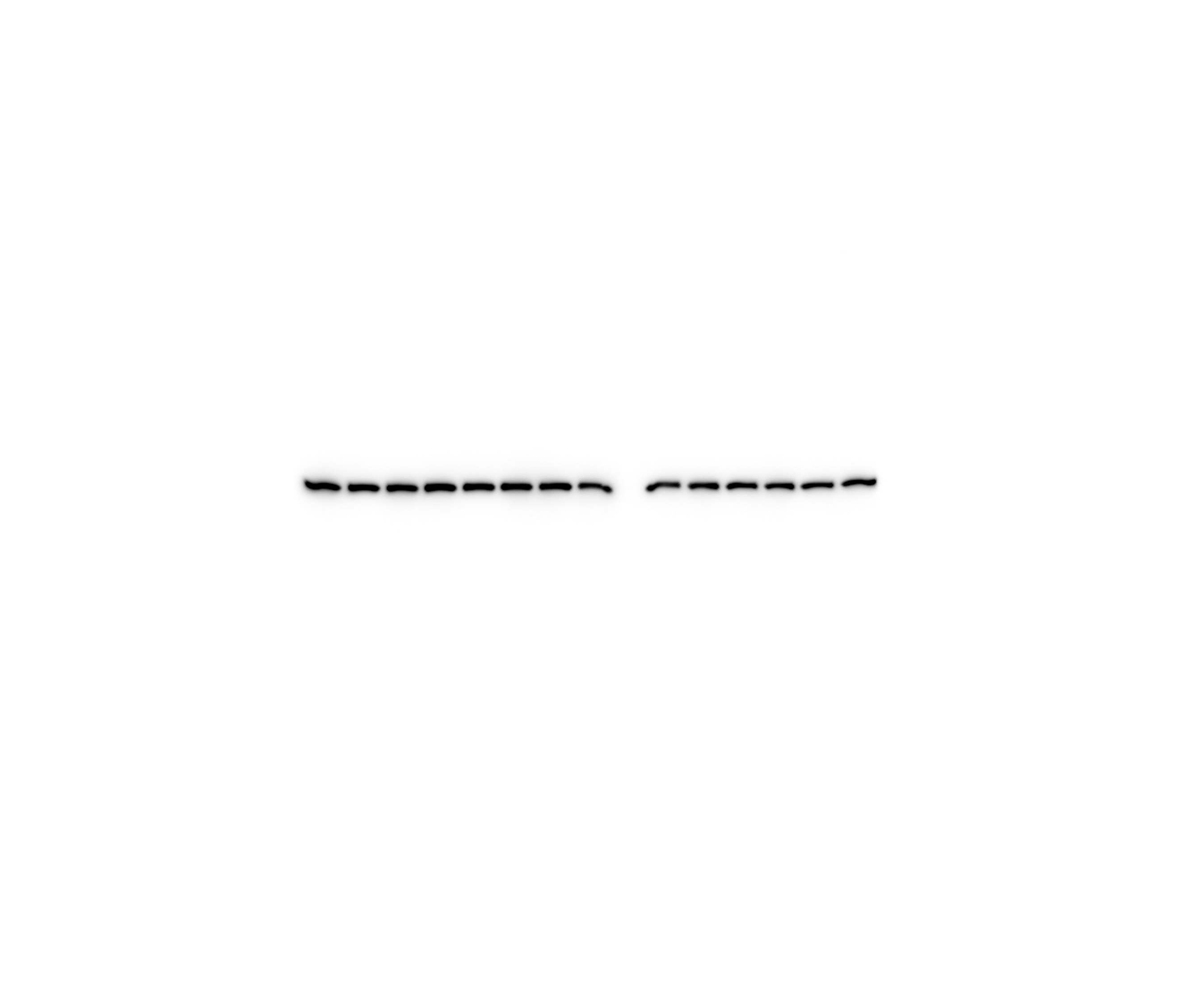

Supplement: Figure 6—source data 1. [file elife-103996-fig6-data1.zip › elife-103996-fig6-data1-v1/Figure 6B/Figure 6B Actin.tif]

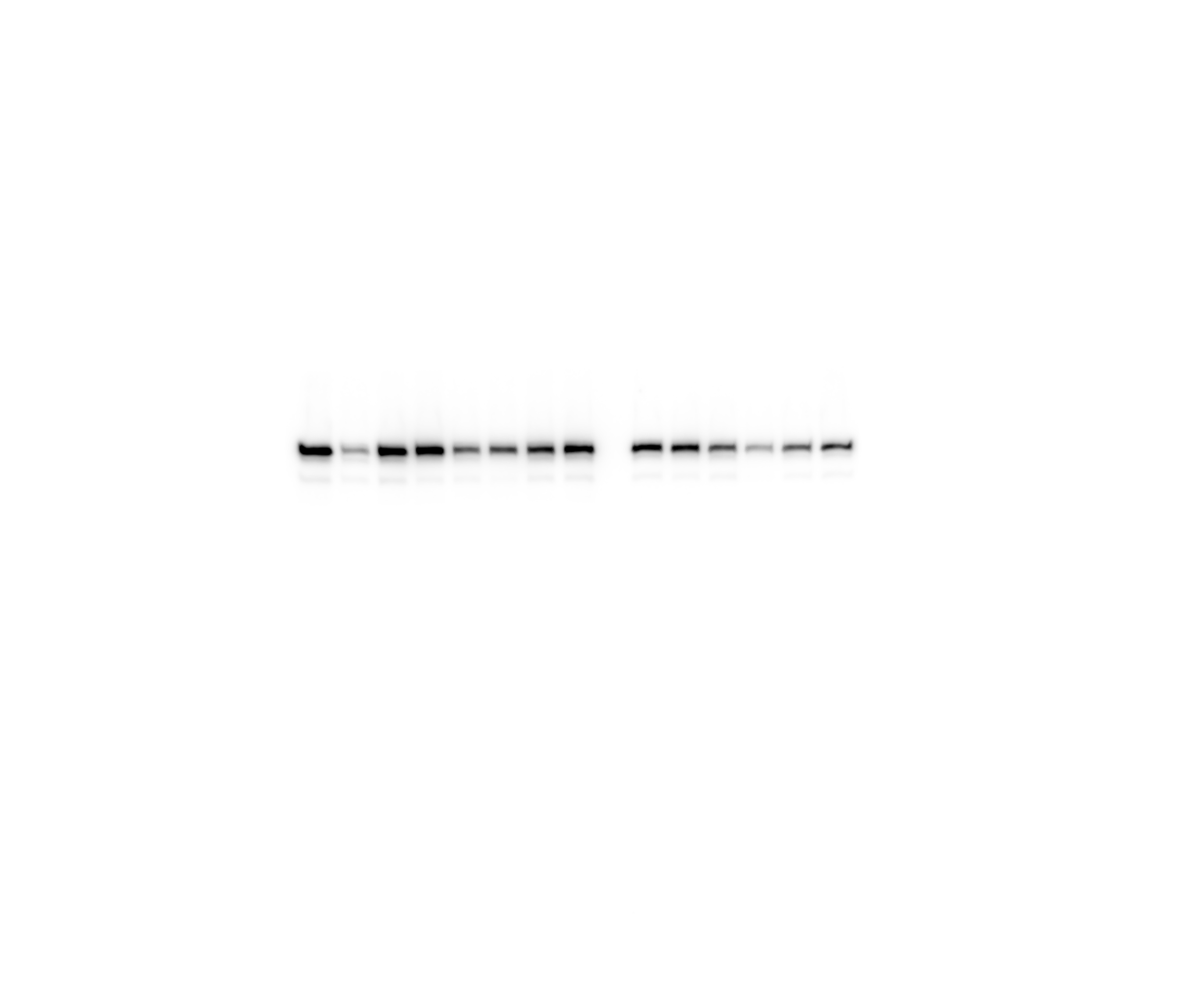

Supplement: Figure 6—source data 1. [file elife-103996-fig6-data1.zip › elife-103996-fig6-data1-v1/Figure 6B/Figure 6B b-cat.tif]

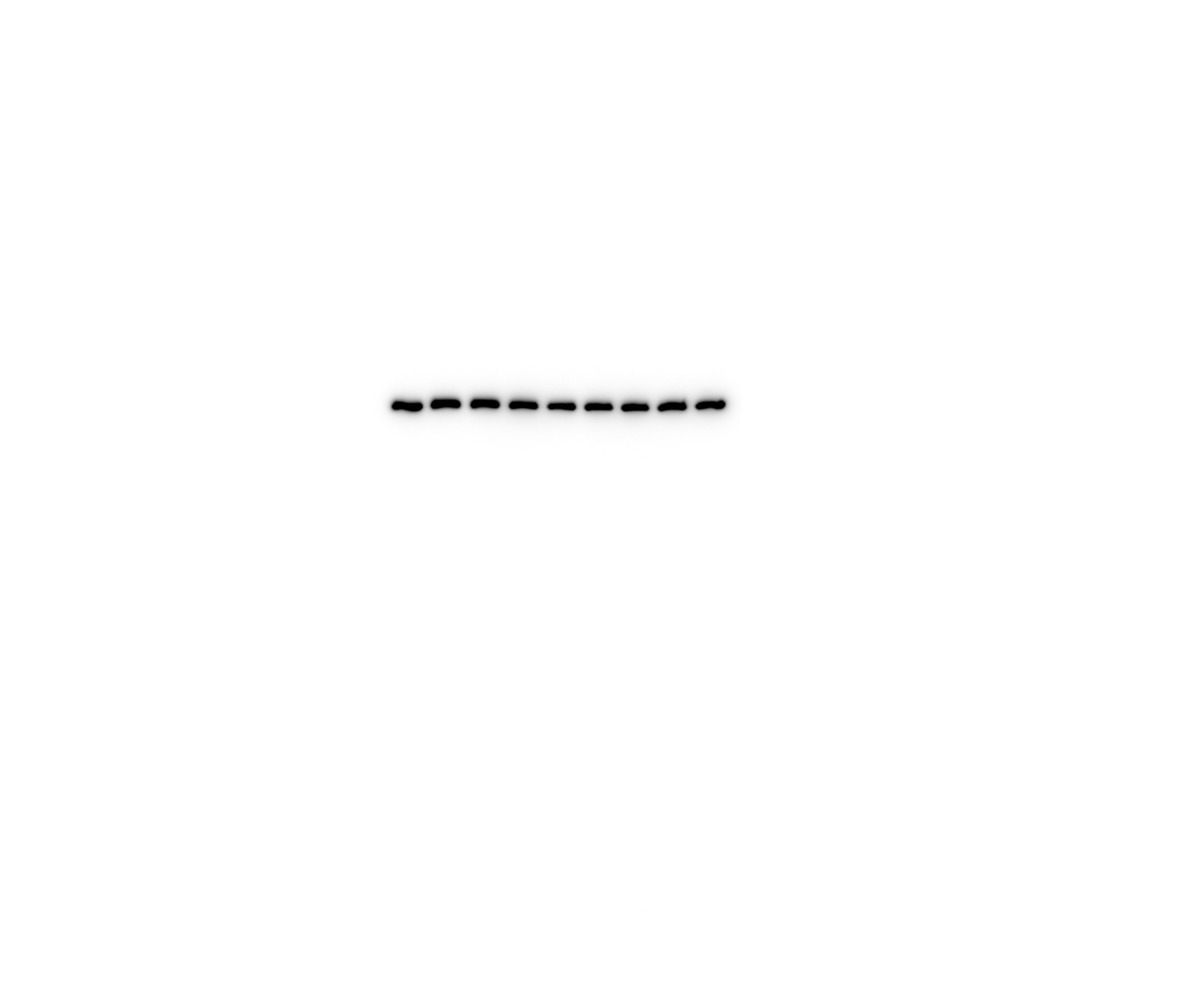

Supplement: Figure 6—source data 1. [file elife-103996-fig6-data1.zip › elife-103996-fig6-data1-v1/Figure 6C/Figure 6C Actin.tif]

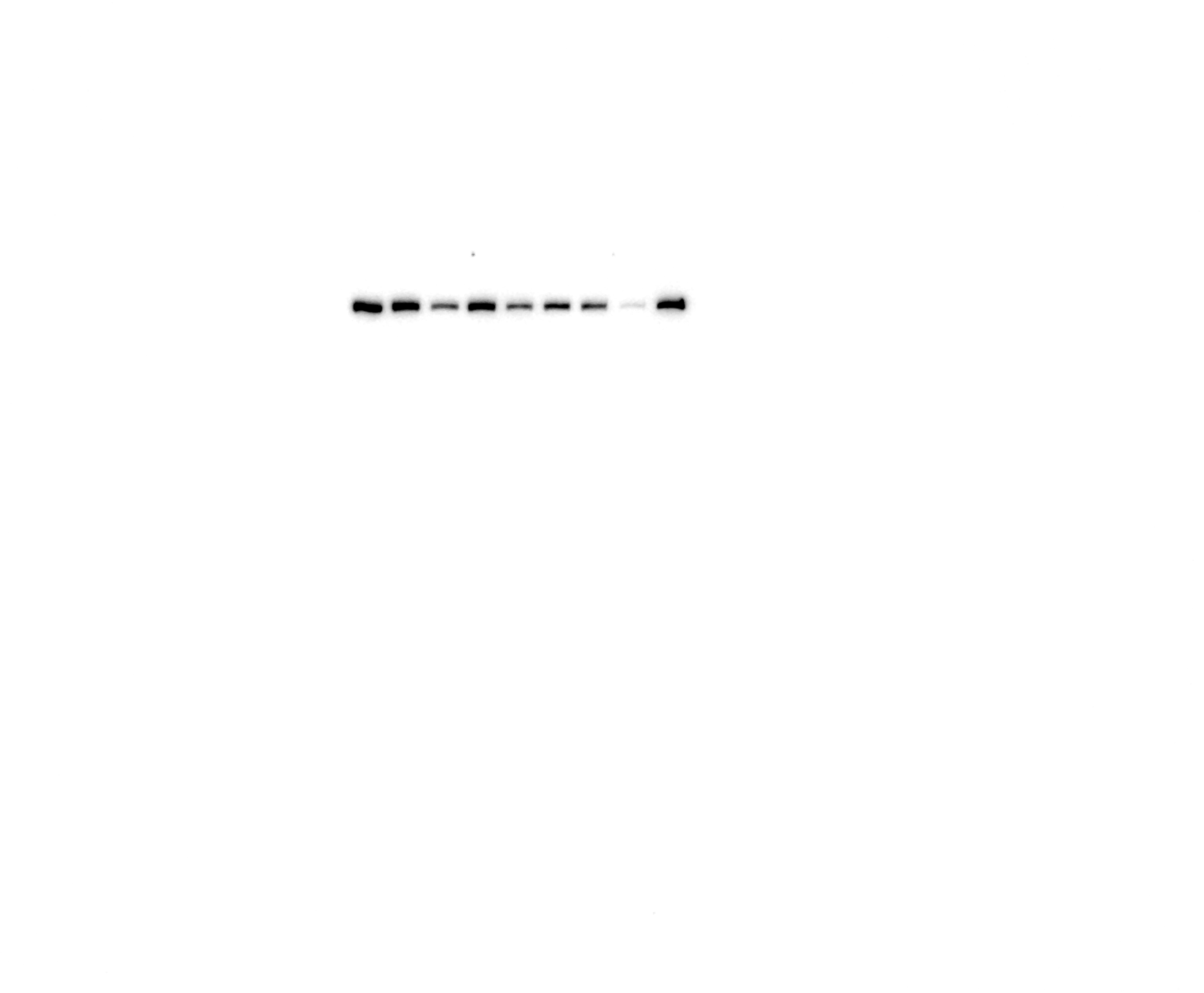

Supplement: Figure 6—source data 1. [file elife-103996-fig6-data1.zip › elife-103996-fig6-data1-v1/Figure 6C/Figure 6C b-cat.tif]

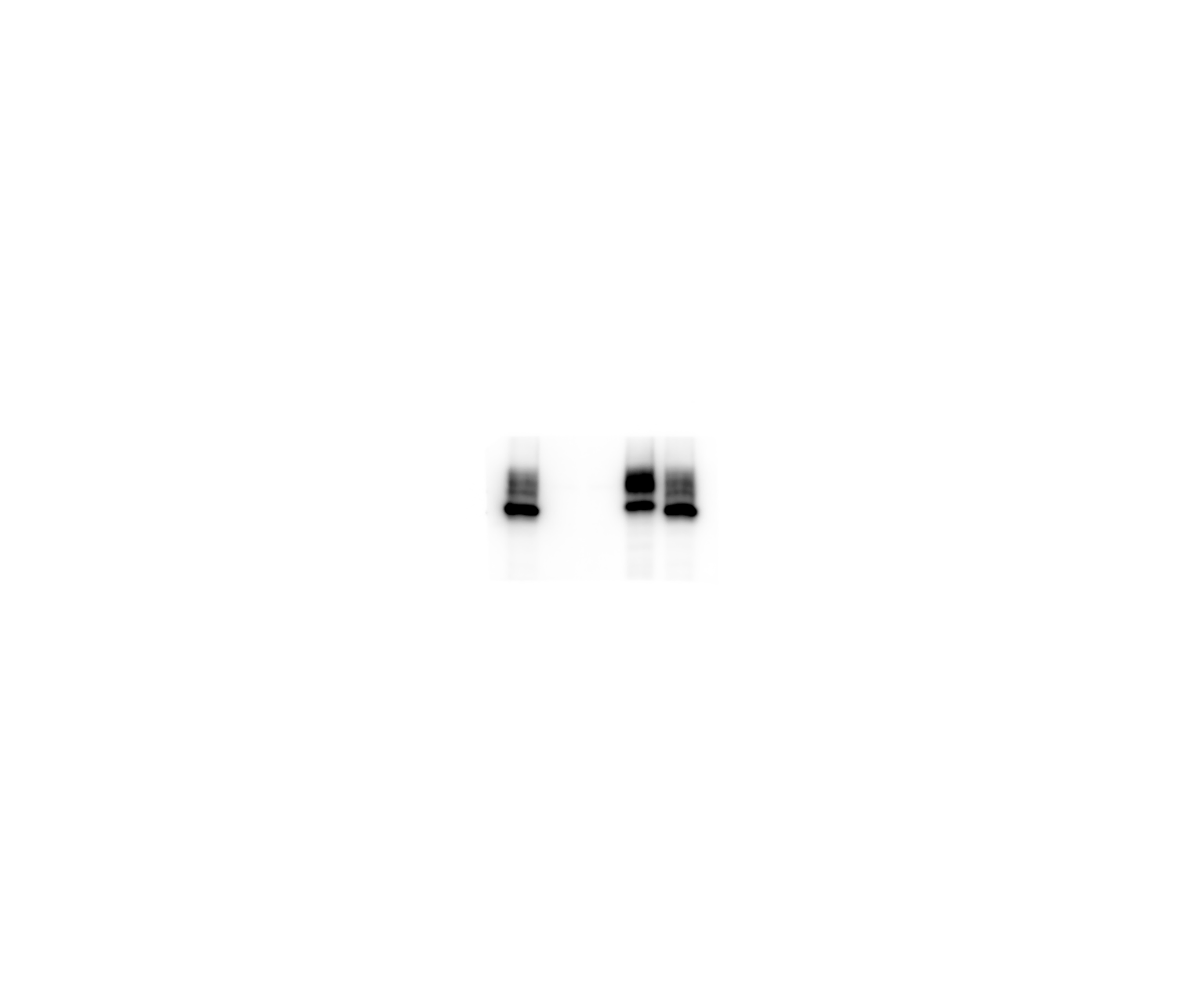

Supplement: Figure 6—source data 1. [file elife-103996-fig6-data1.zip › elife-103996-fig6-data1-v1/Figure 6C/Figure 6C V5.tif]

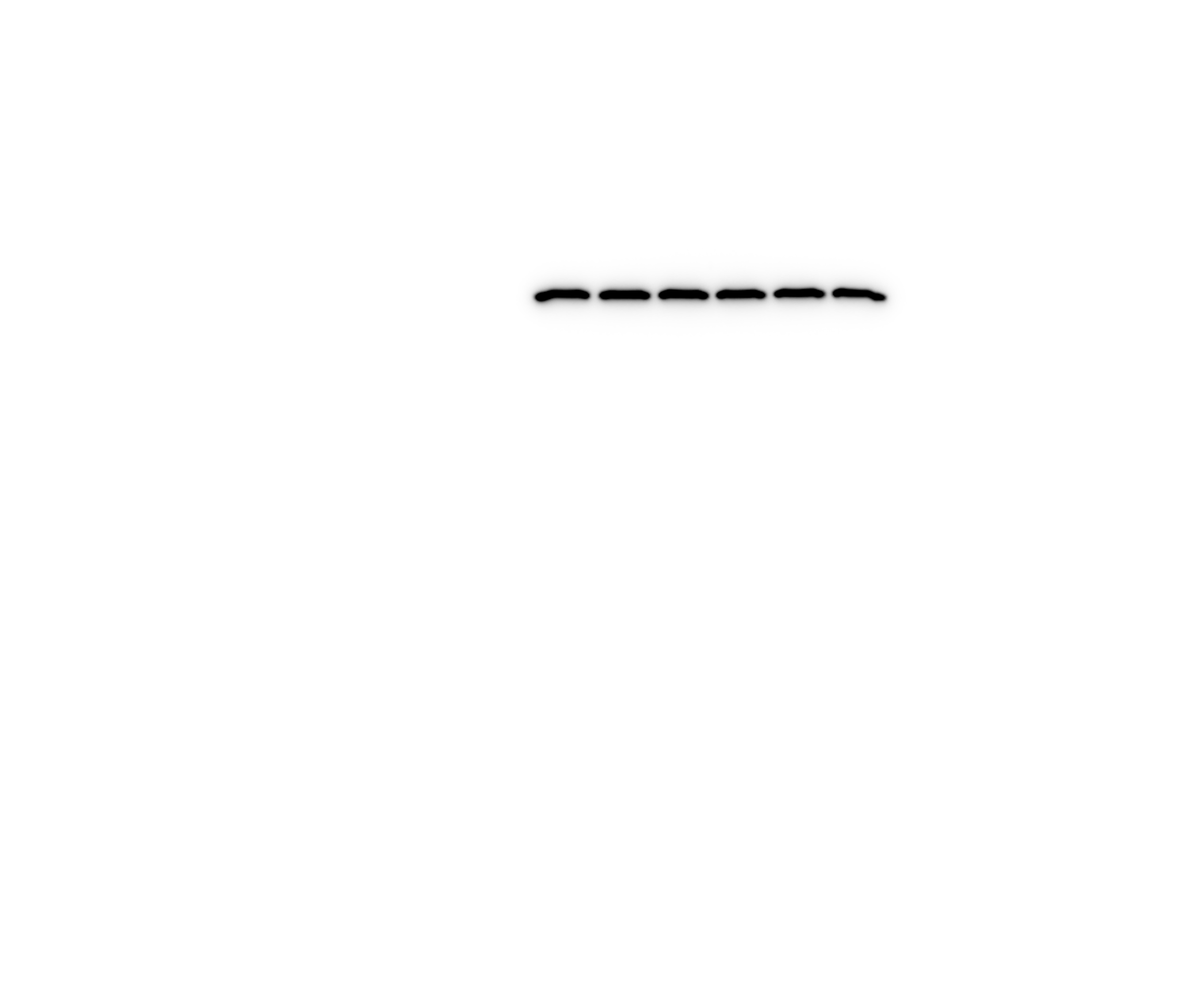

Supplement: Figure 6—source data 1. [file elife-103996-fig6-data1.zip › elife-103996-fig6-data1-v1/Figure 6D/Figure 6D Actin.tif]

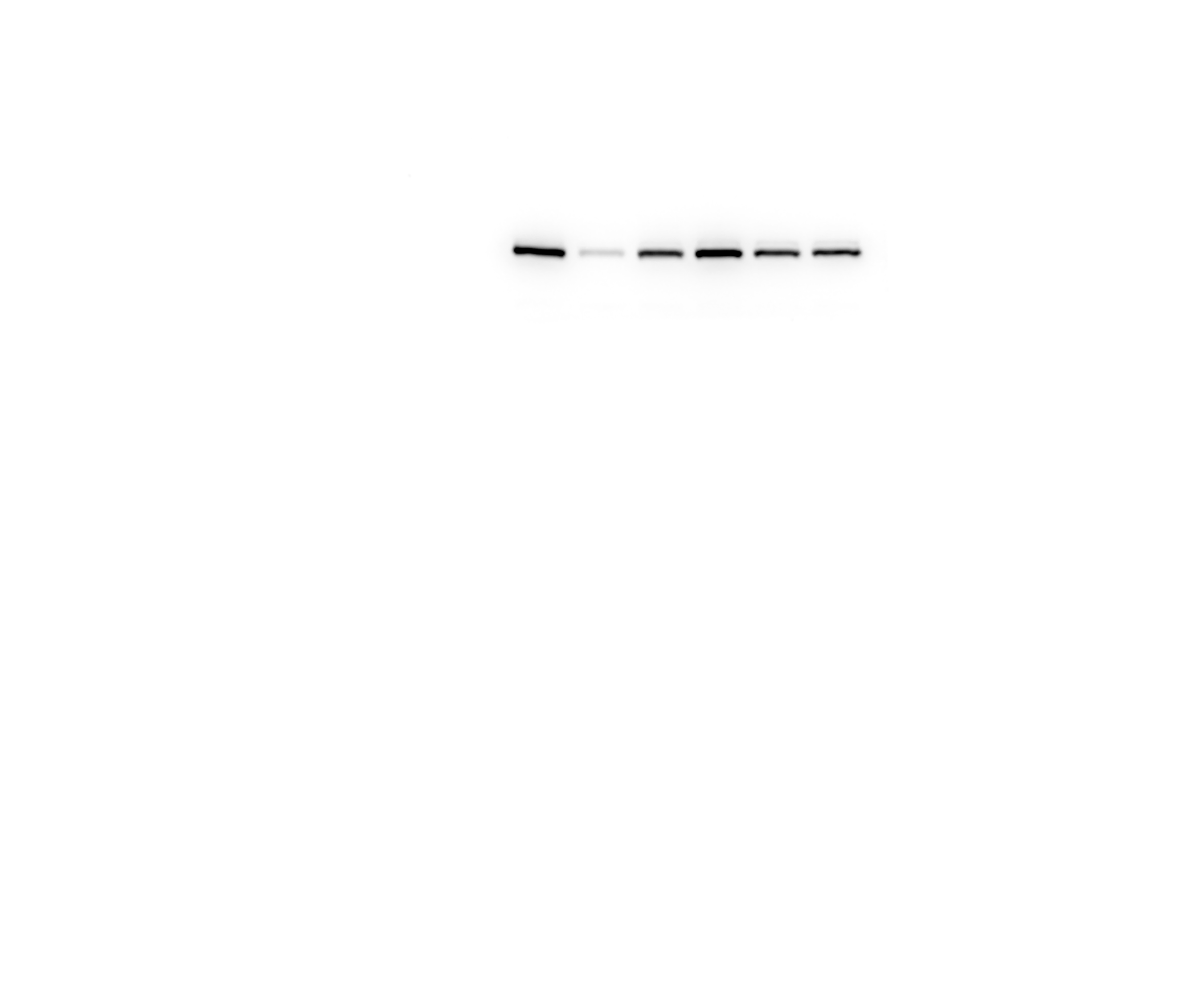

Supplement: Figure 6—source data 1. [file elife-103996-fig6-data1.zip › elife-103996-fig6-data1-v1/Figure 6D/Figure 6D b-cat.tif]

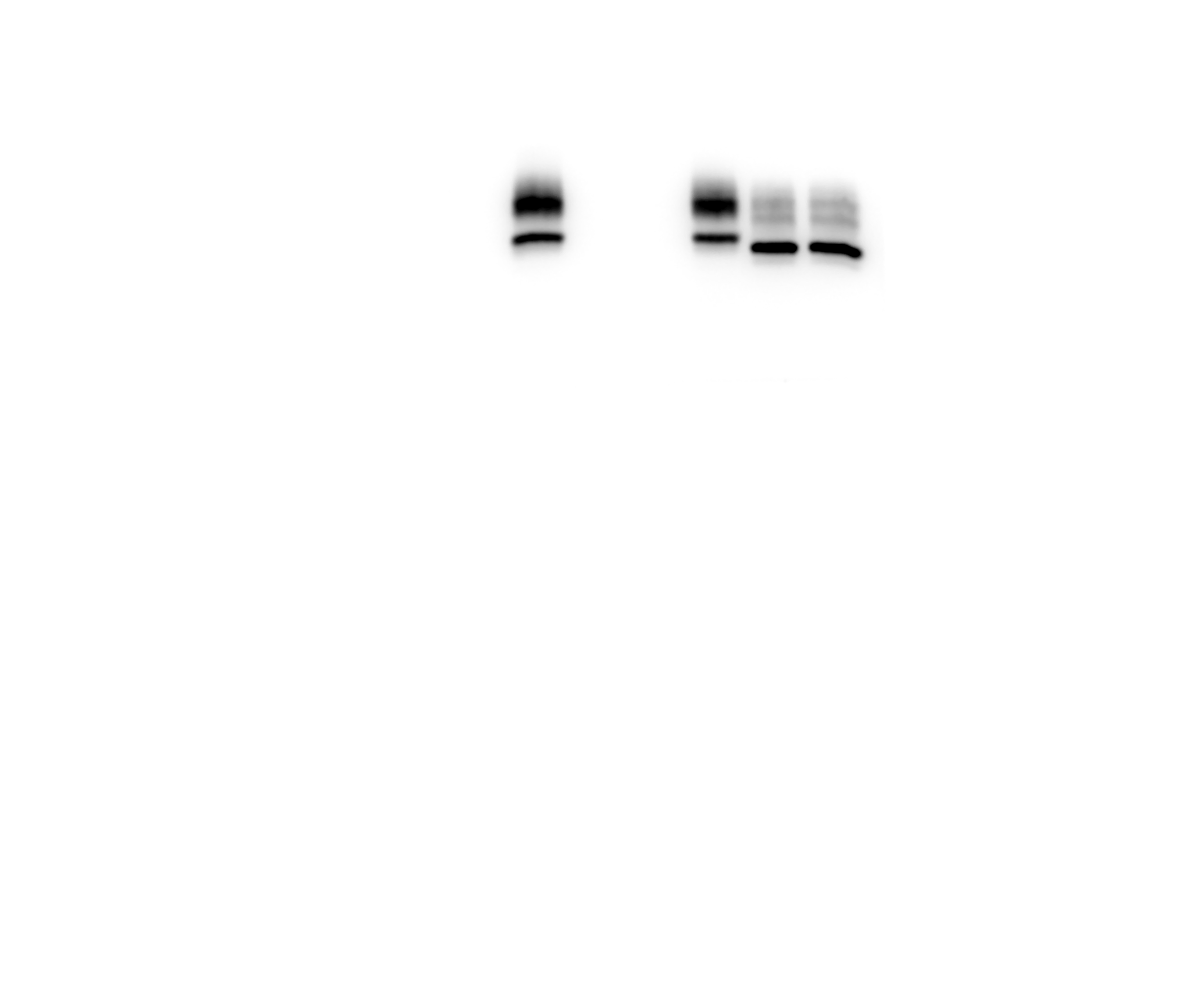

Supplement: Figure 6—source data 1. [file elife-103996-fig6-data1.zip › elife-103996-fig6-data1-v1/Figure 6D/Figure 6D V5.tif]

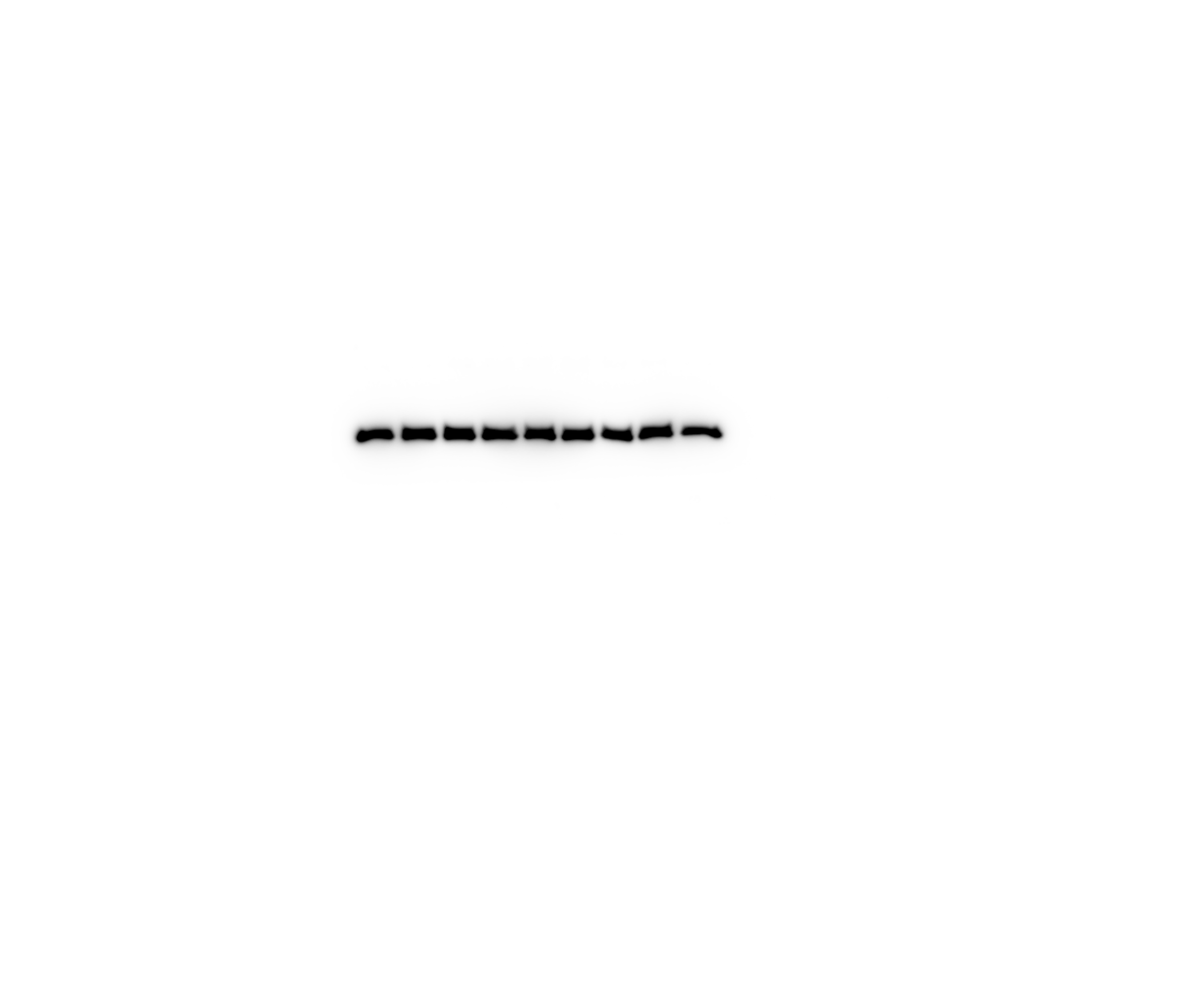

Supplement: Figure 6—source data 1. [file elife-103996-fig6-data1.zip › elife-103996-fig6-data1-v1/Figure 6E F/Figure 6E Actin.tif]

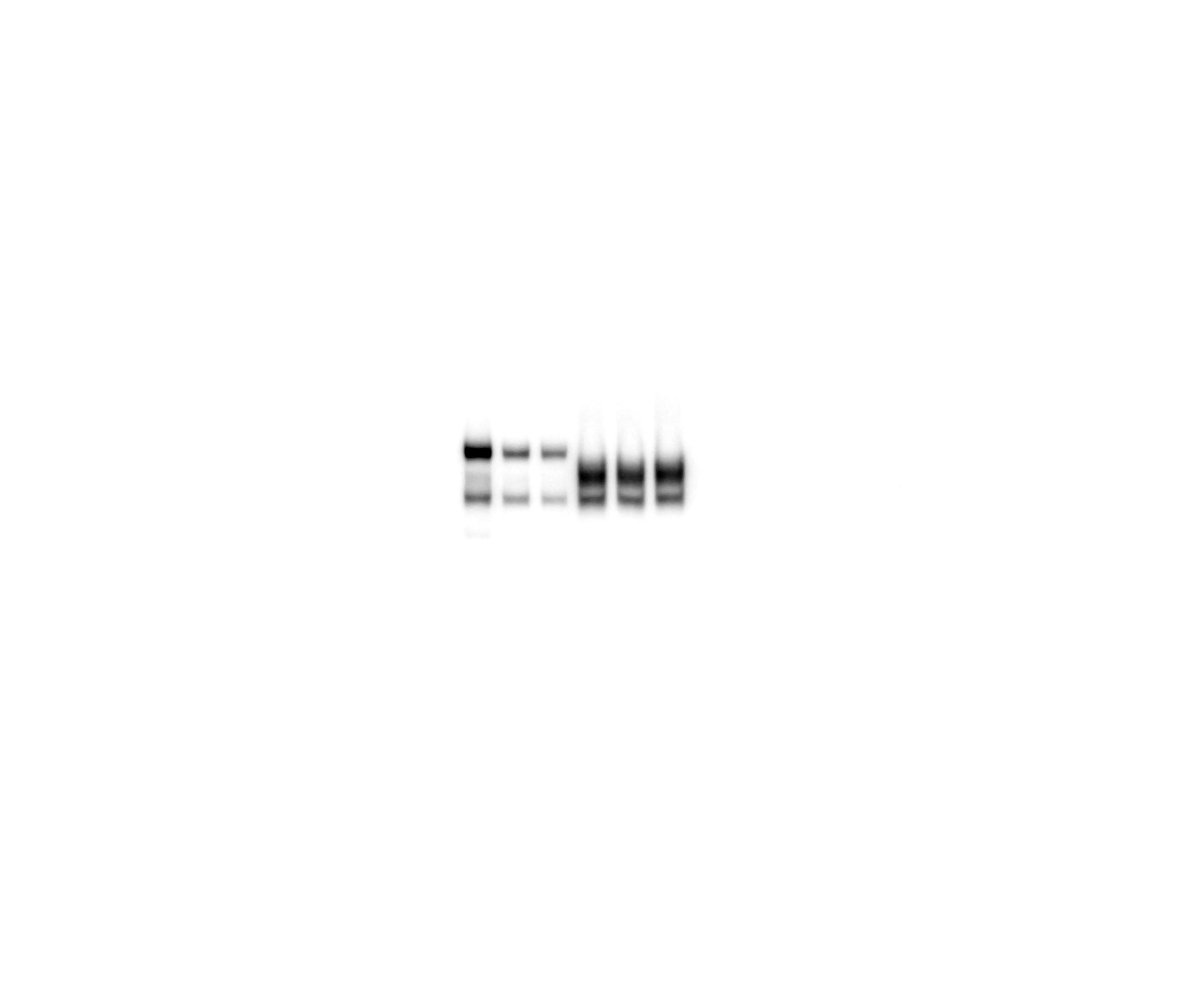

Supplement: Figure 6—source data 1. [file elife-103996-fig6-data1.zip › elife-103996-fig6-data1-v1/Figure 6E F/Figure 6E HA.tif]

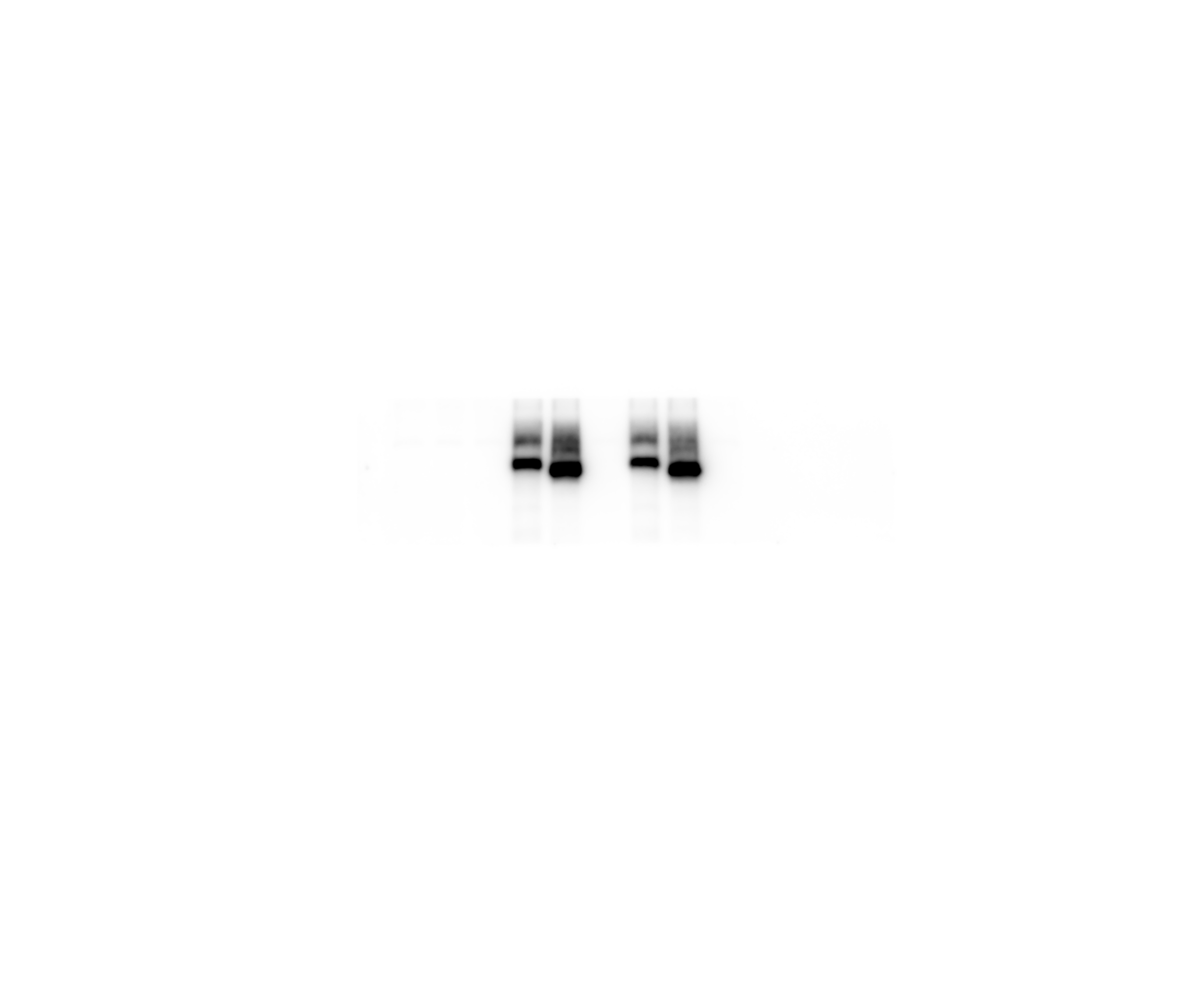

Supplement: Figure 6—source data 1. [file elife-103996-fig6-data1.zip › elife-103996-fig6-data1-v1/Figure 6E F/Figure 6E V5.tif]

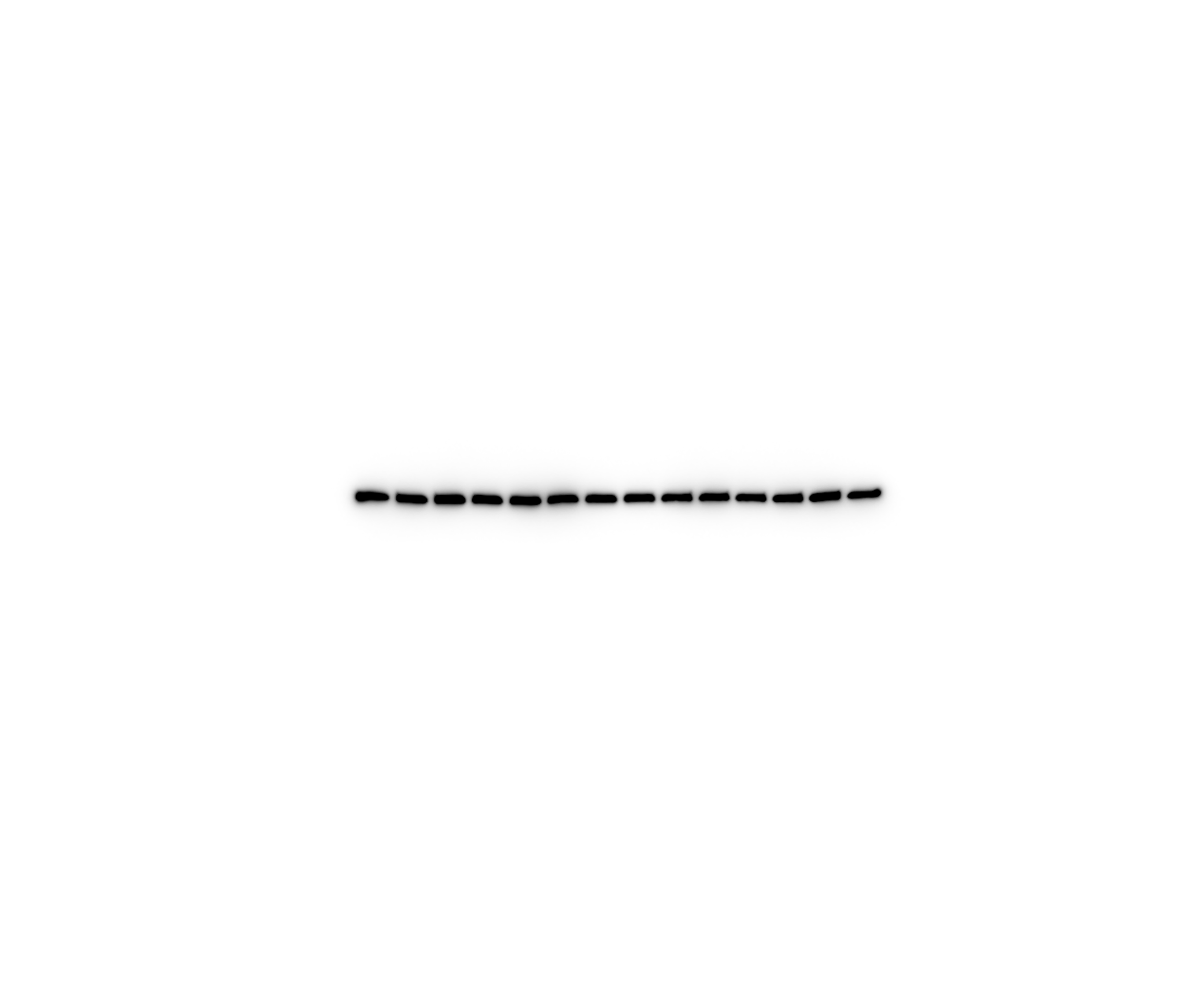

Supplement: Figure 6—source data 1. [file elife-103996-fig6-data1.zip › elife-103996-fig6-data1-v1/Figure 6E F/Figure 6F Actin.tif]

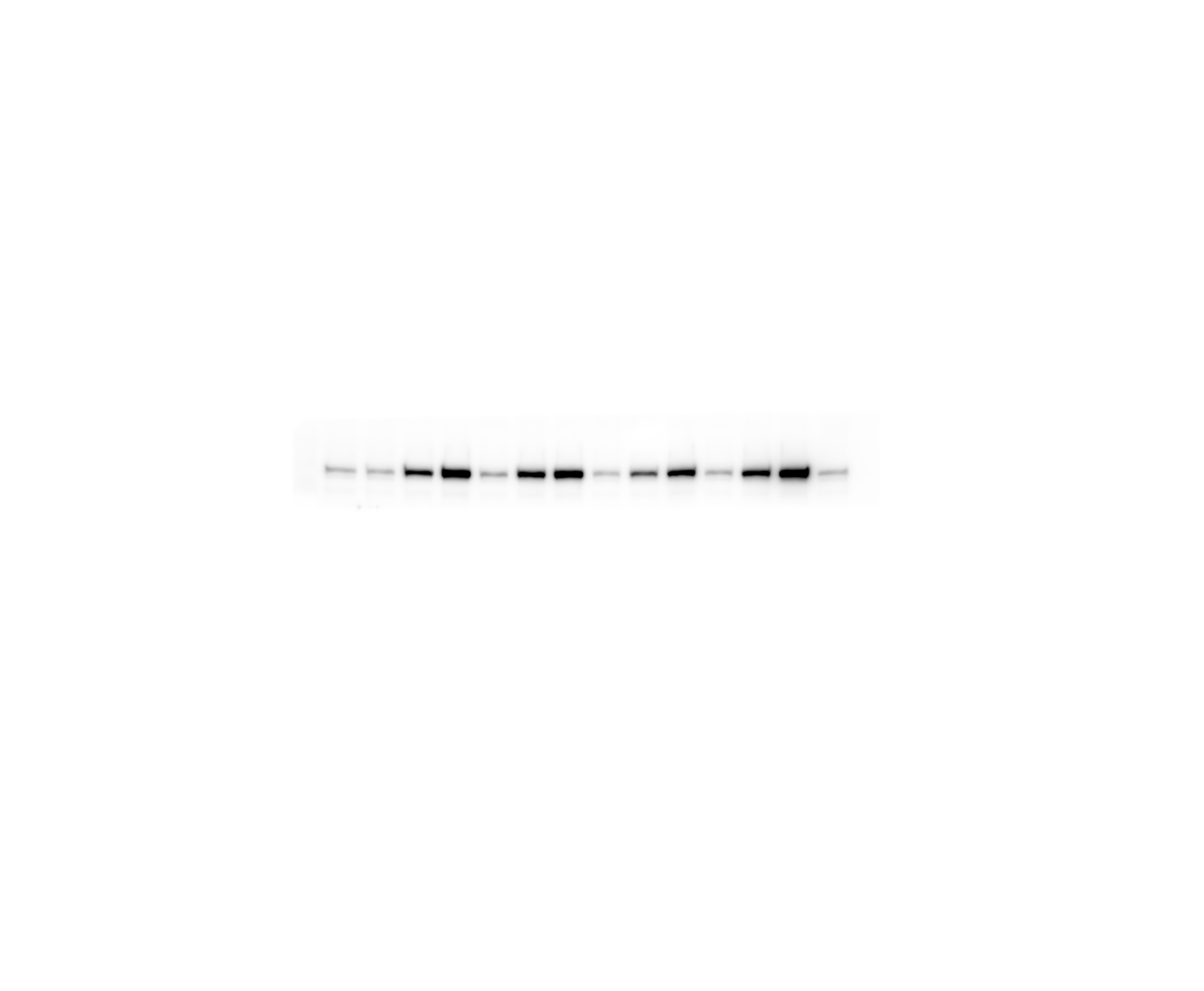

Supplement: Figure 6—source data 1. [file elife-103996-fig6-data1.zip › elife-103996-fig6-data1-v1/Figure 6E F/Figure 6F b-cat.tif]

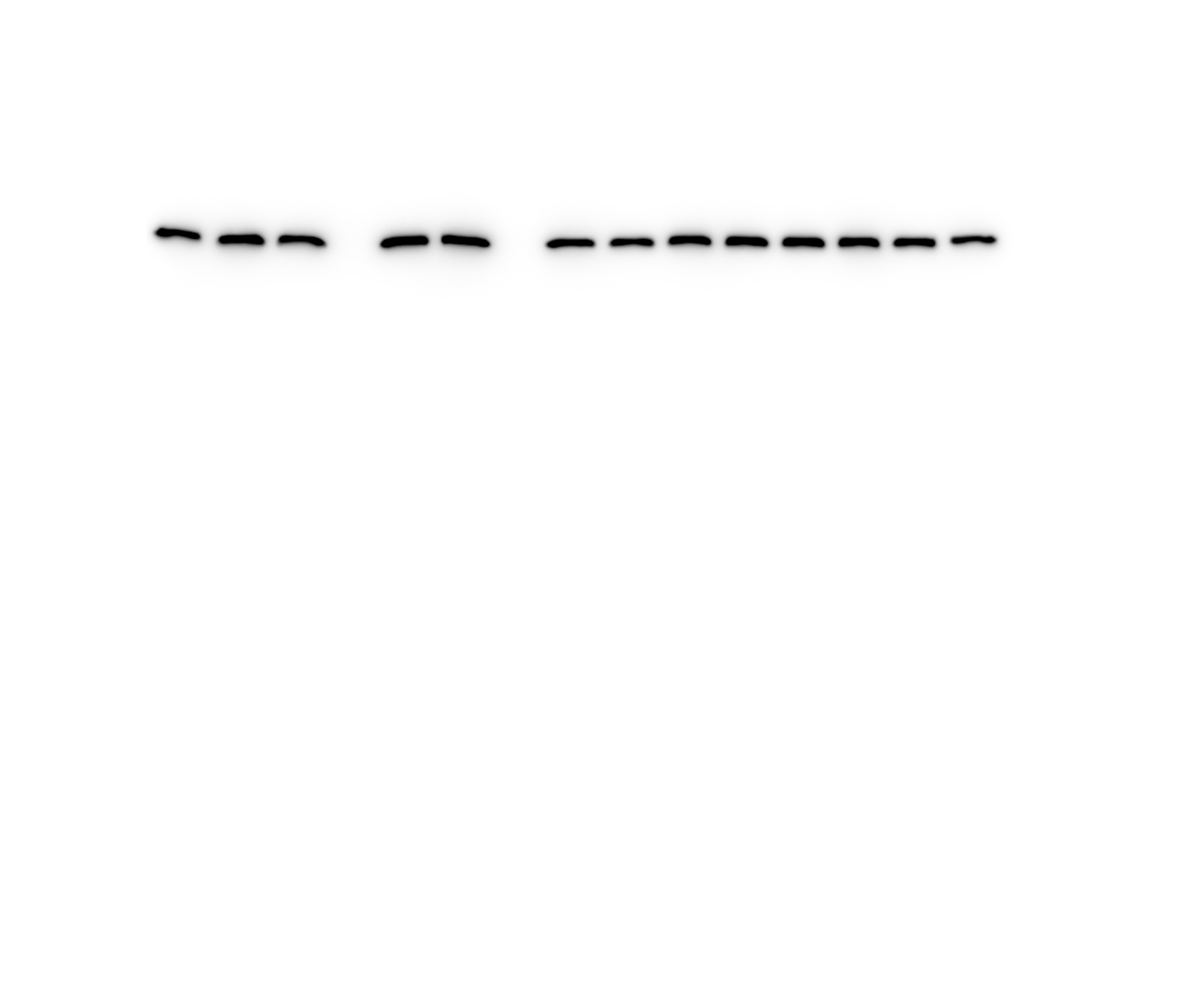

Supplement: Figure 6—source data 1. [file elife-103996-fig6-data1.zip › elife-103996-fig6-data1-v1/Figure 6H I/Figure 6H Actin.tif]

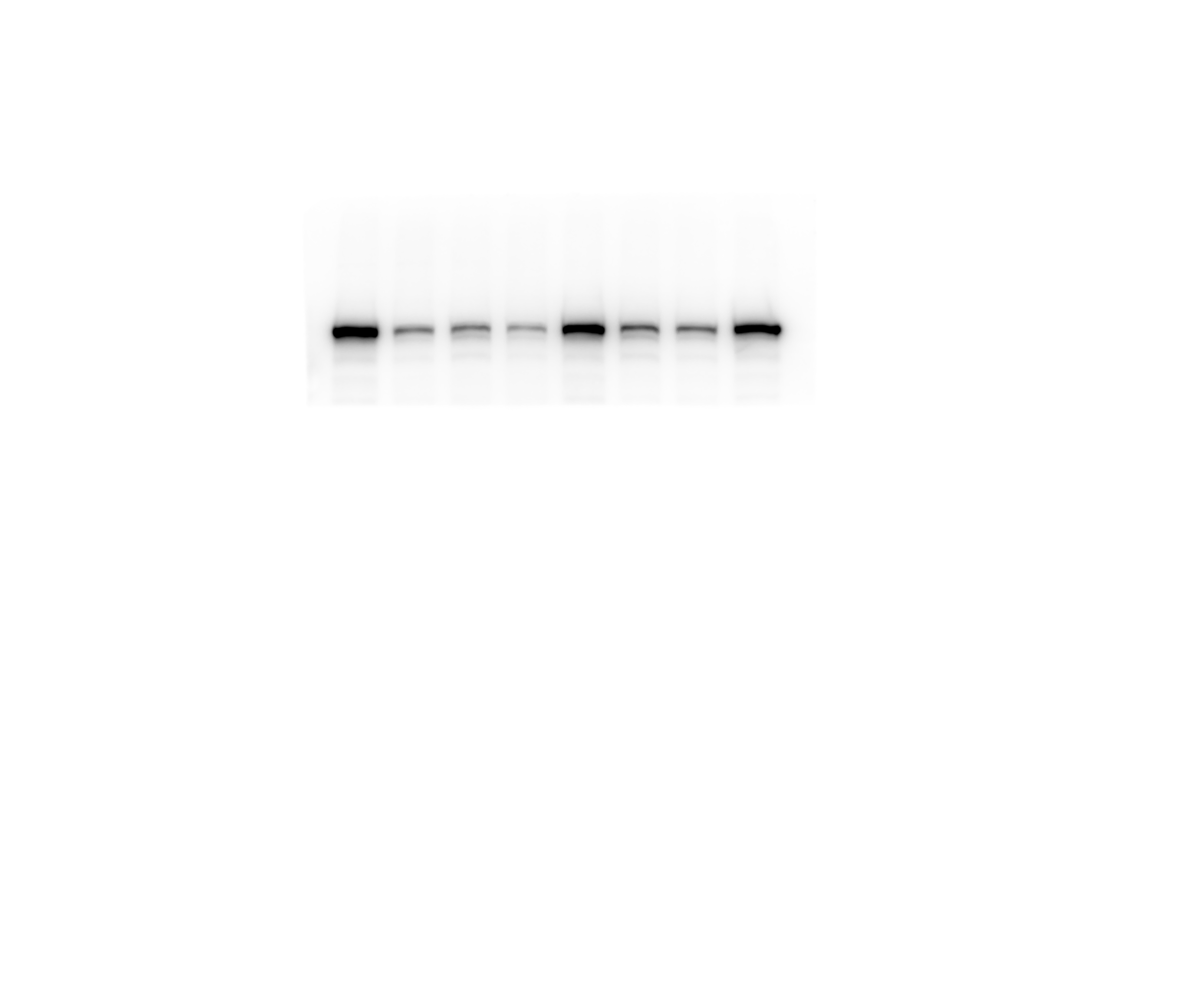

Supplement: Figure 6—source data 1. [file elife-103996-fig6-data1.zip › elife-103996-fig6-data1-v1/Figure 6H I/Figure 6H b-cat.tif]

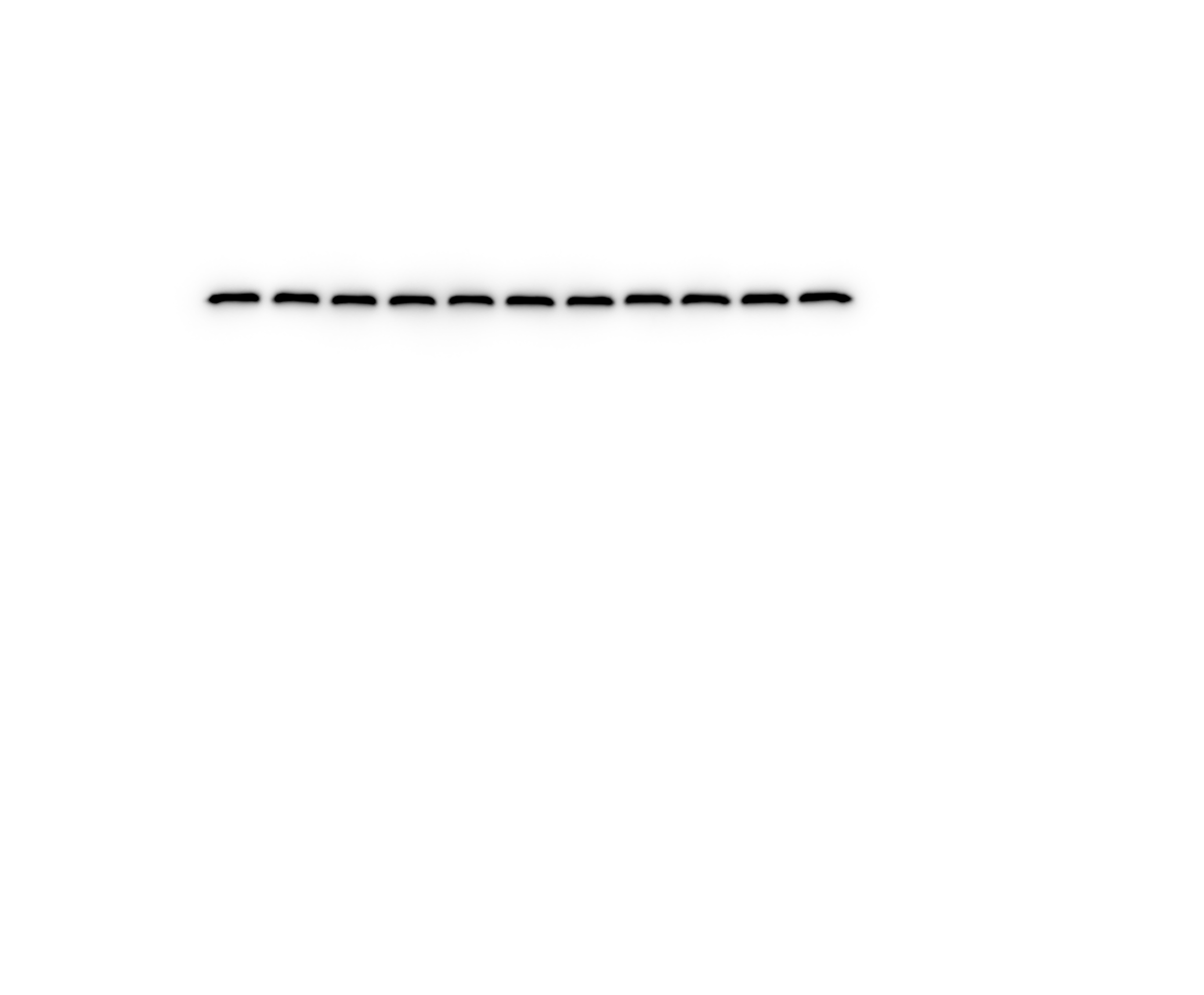

Supplement: Figure 6—source data 1. [file elife-103996-fig6-data1.zip › elife-103996-fig6-data1-v1/Figure 6H I/Figure 6I Actin.tif]

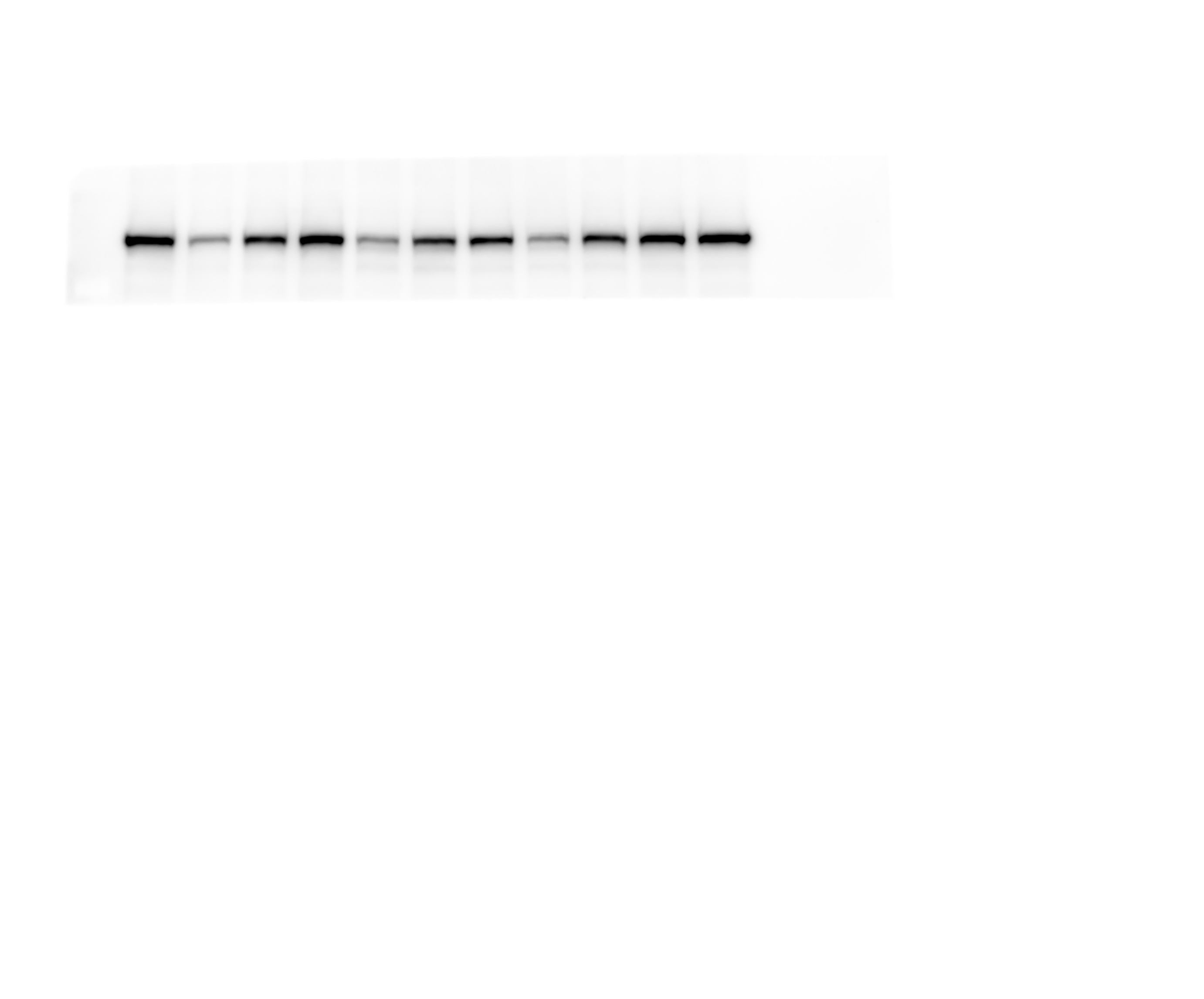

Supplement: Figure 6—source data 1. [file elife-103996-fig6-data1.zip › elife-103996-fig6-data1-v1/Figure 6H I/Figure 6I b-cat.tif]

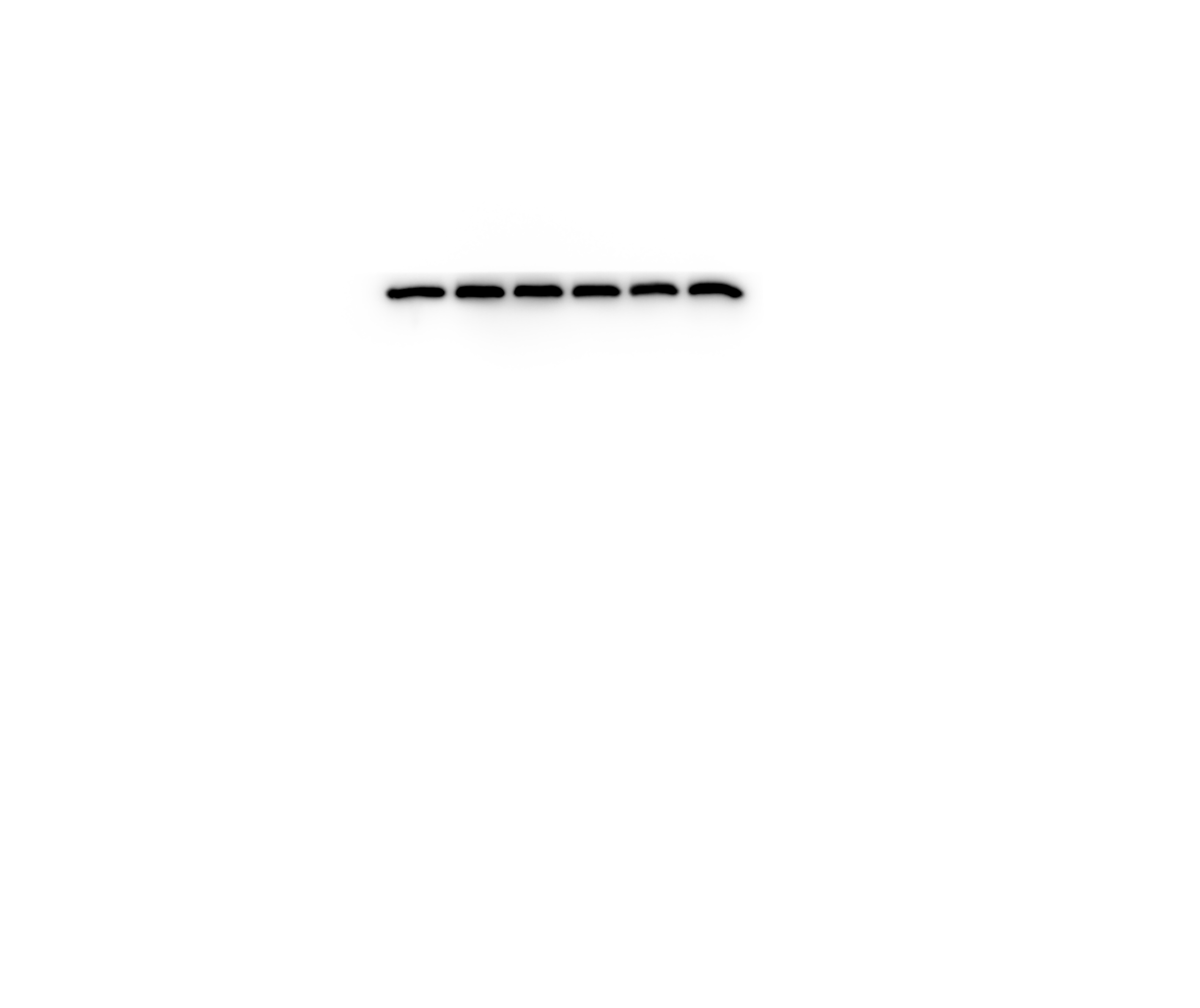

Supplement: Figure 6—source data 1. [file elife-103996-fig6-data1.zip › elife-103996-fig6-data1-v1/Figure 6J K/Figure 6J Actin.tif]

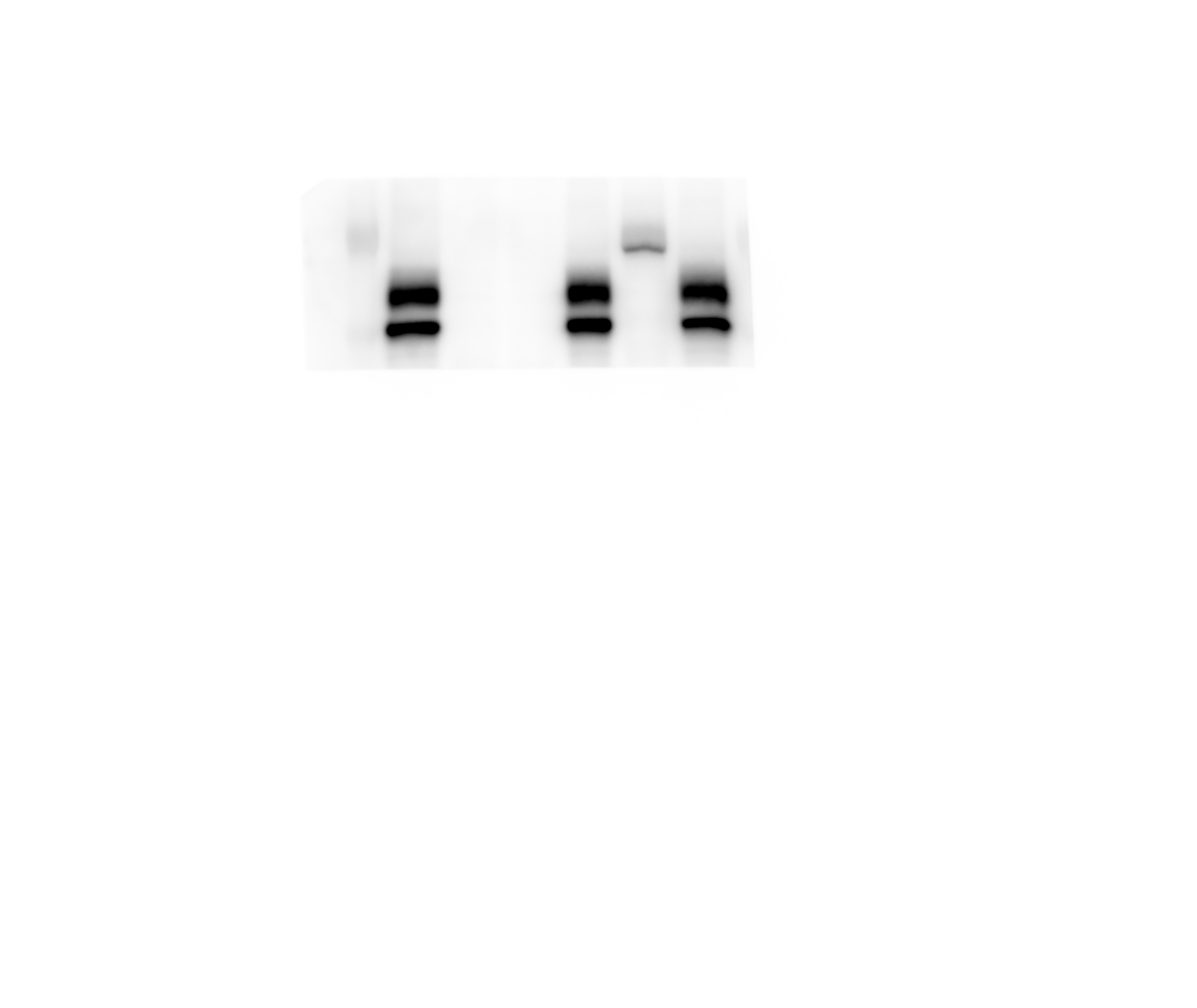

Supplement: Figure 6—source data 1. [file elife-103996-fig6-data1.zip › elife-103996-fig6-data1-v1/Figure 6J K/Figure 6J V5.tif]

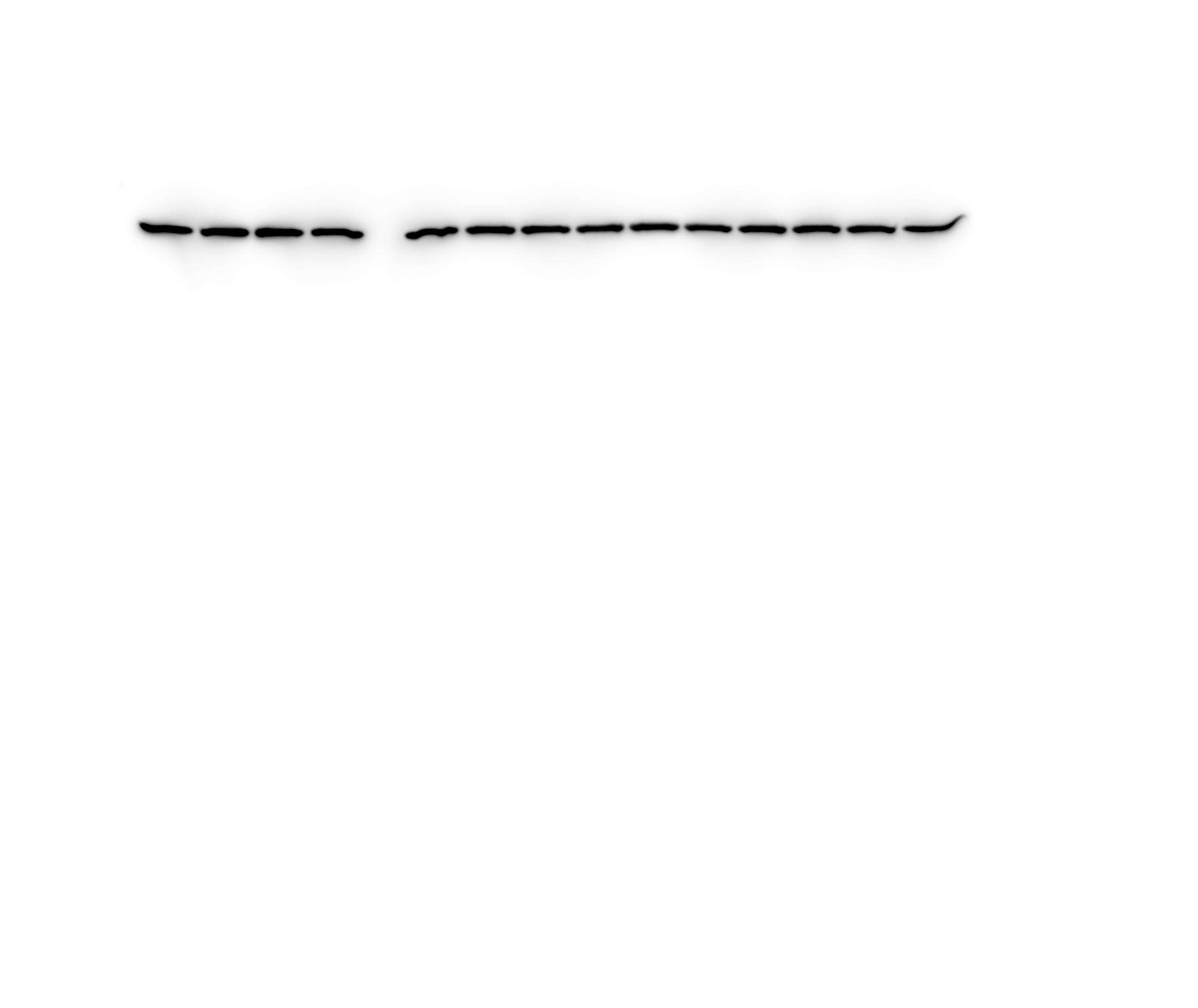

Supplement: Figure 6—source data 1. [file elife-103996-fig6-data1.zip › elife-103996-fig6-data1-v1/Figure 6J K/Figure 6K Actin.tif]

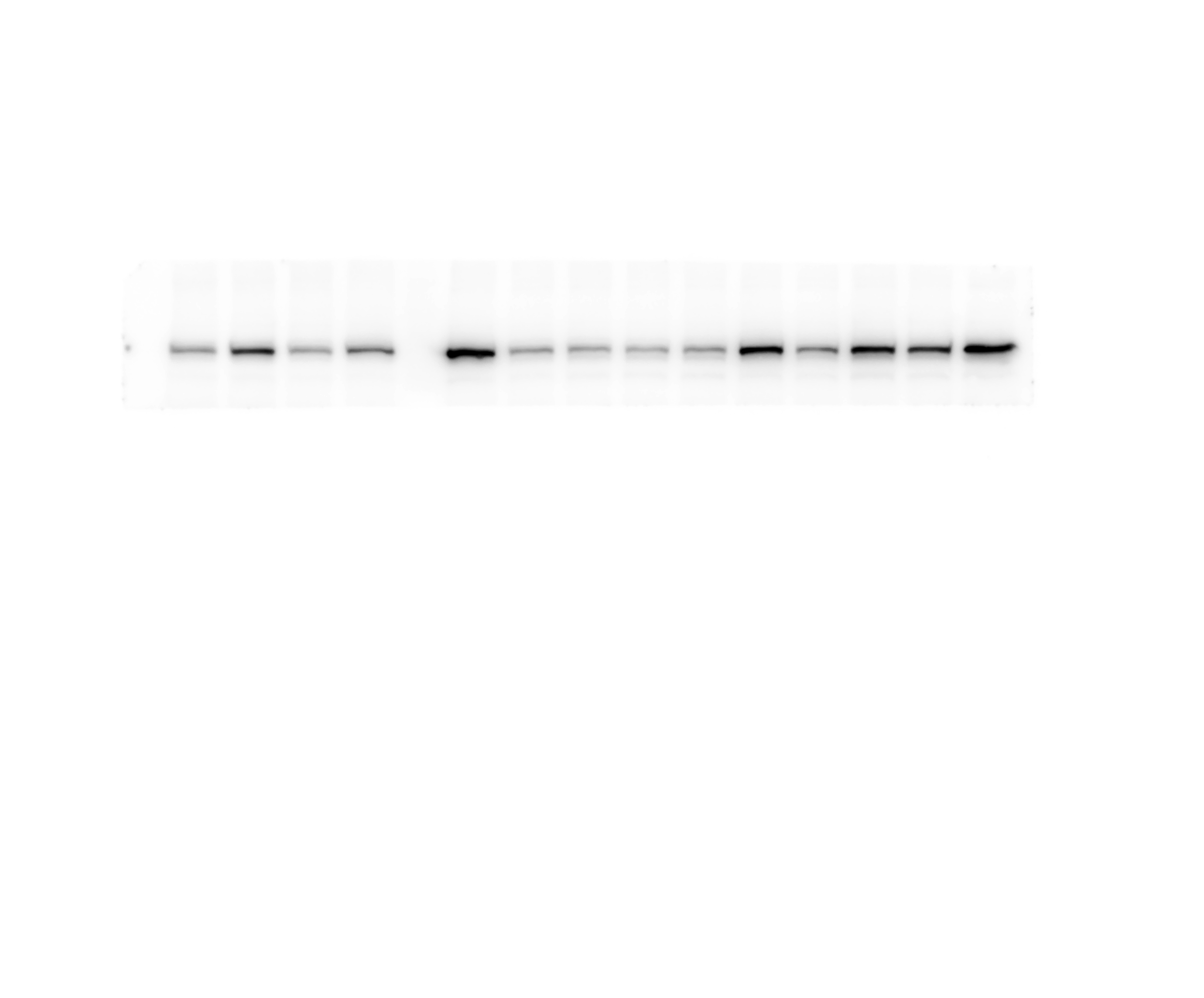

Supplement: Figure 6—source data 1. [file elife-103996-fig6-data1.zip › elife-103996-fig6-data1-v1/Figure 6J K/Figure 6K b-cat.tif]

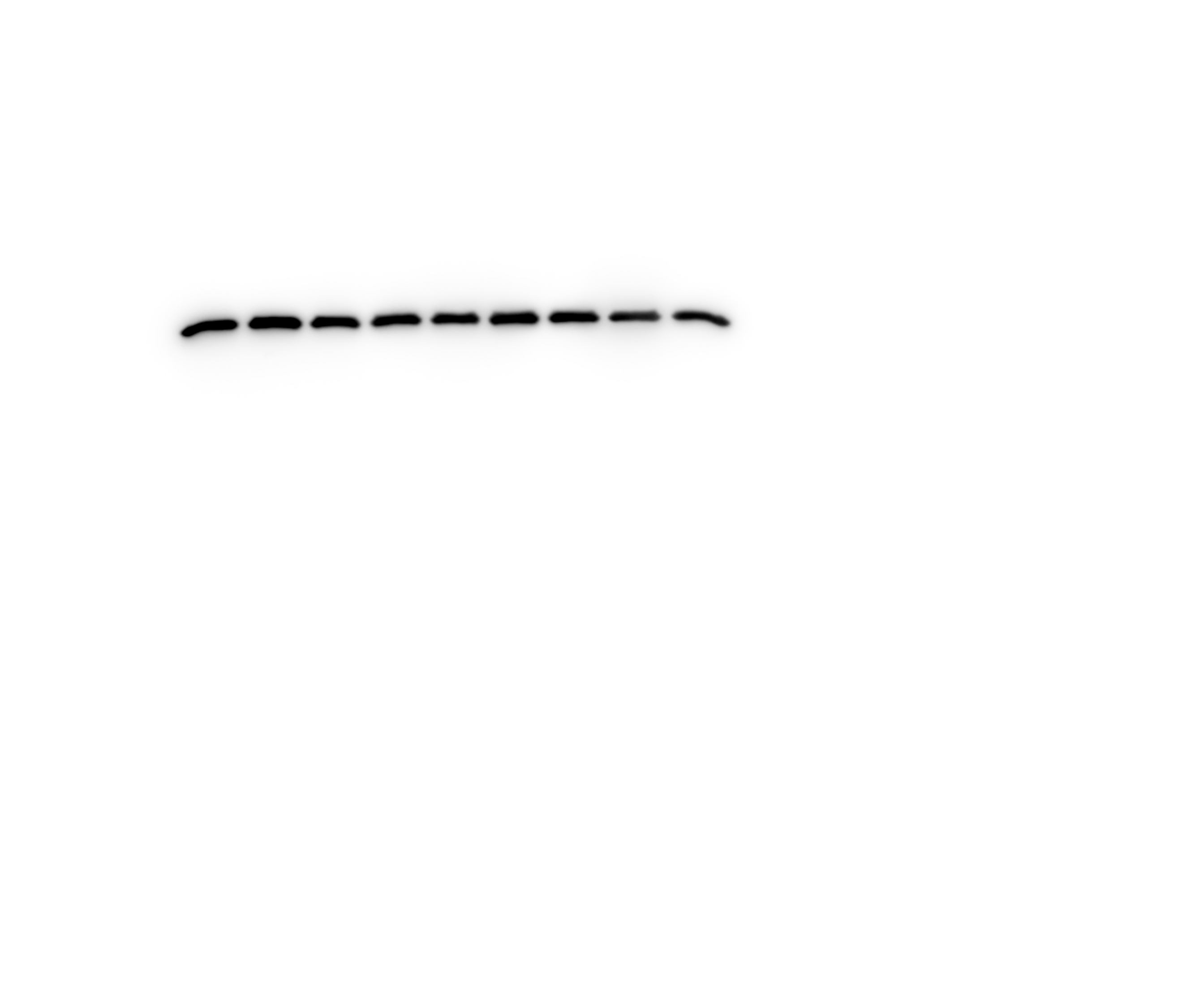

Supplement: Figure 6—figure supplement 1—source data 1. [file elife-103996-fig6-figsupp1-data1.zip › elife-103996-fig6-figsupp1-data1-v1/Figure 6-figure supplement 1A Actin.tif]

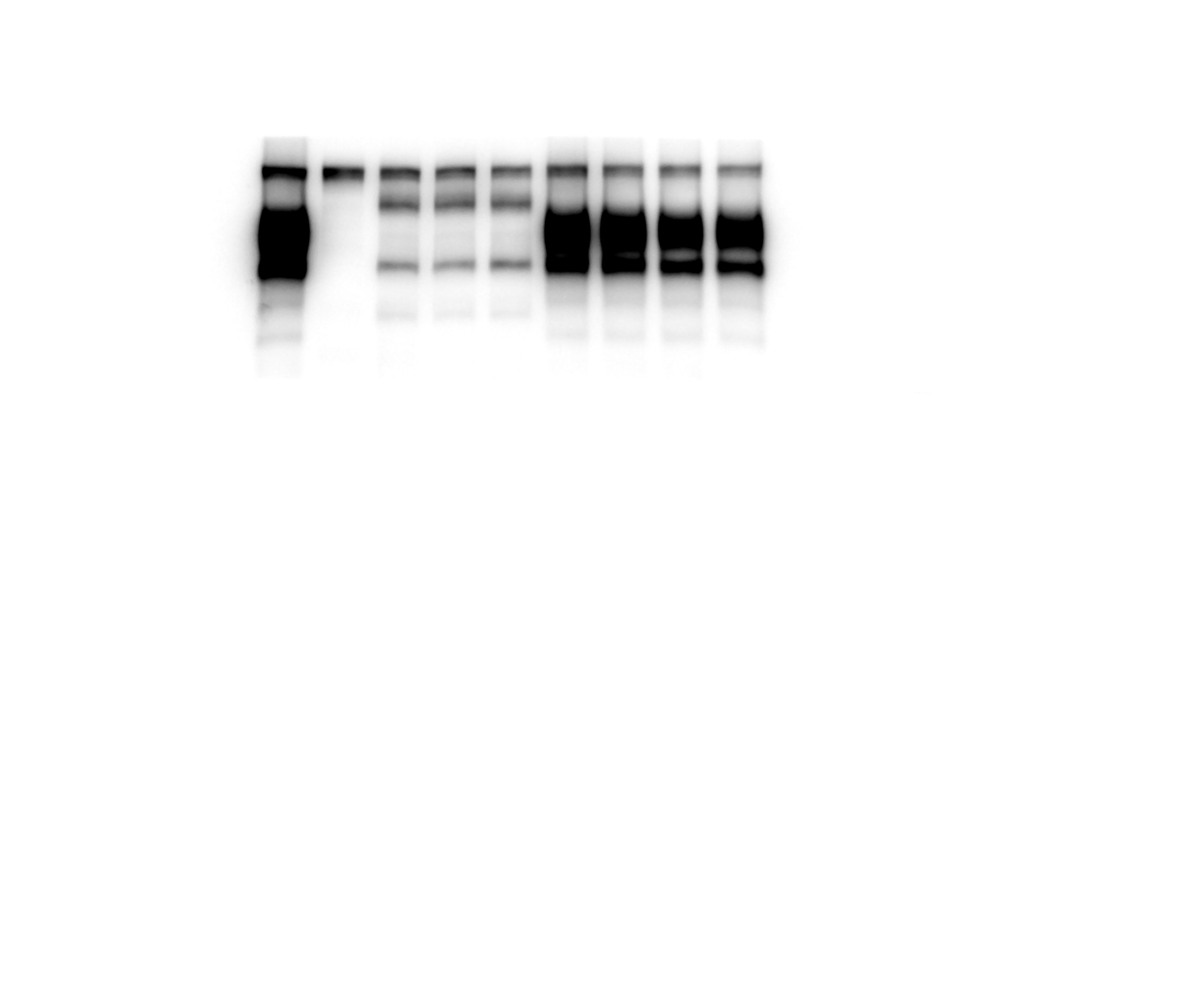

Supplement: Figure 6—figure supplement 1—source data 1. [file elife-103996-fig6-figsupp1-data1.zip › elife-103996-fig6-figsupp1-data1-v1/Figure 6-figure supplement 1A HA.tif]

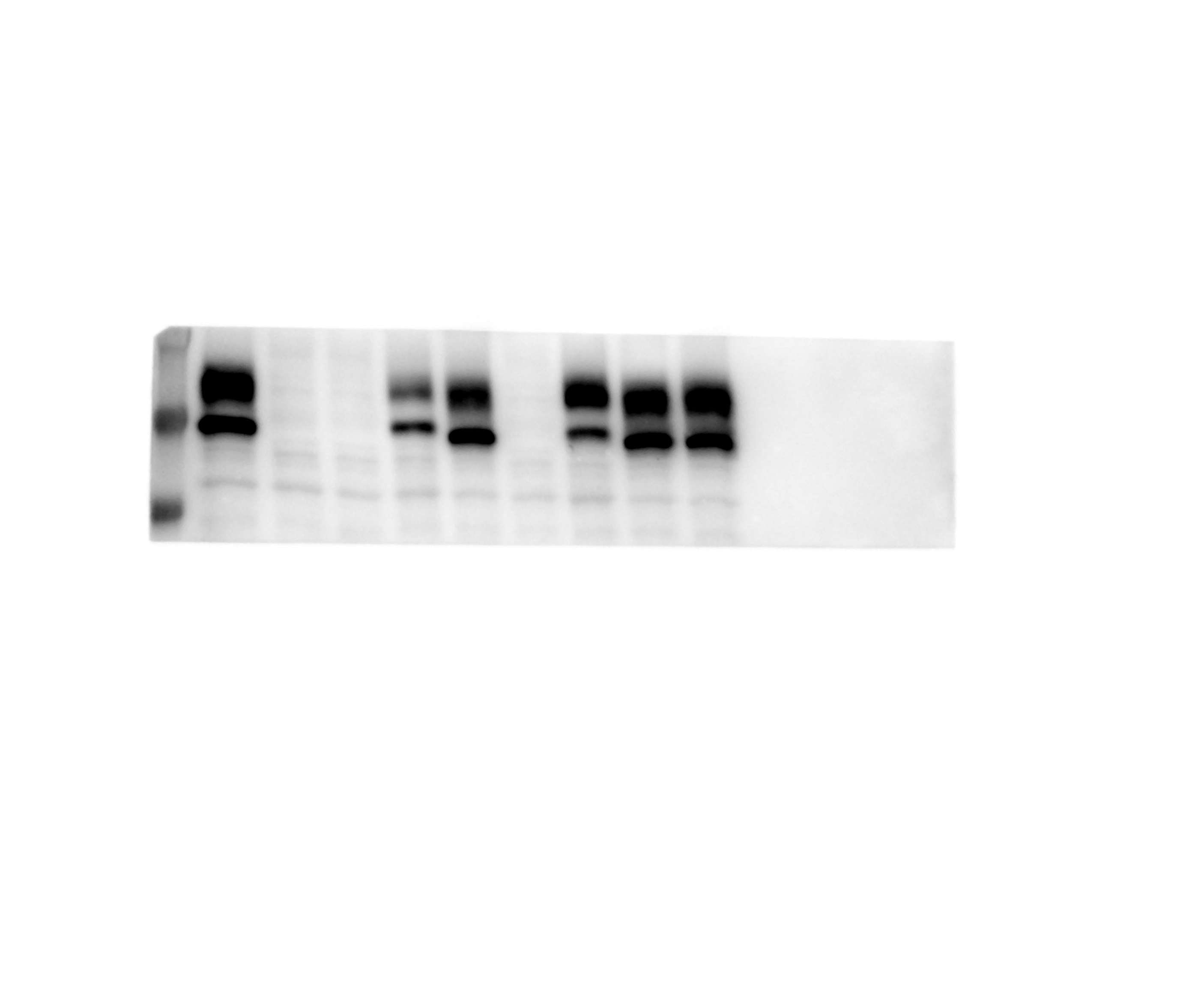

Supplement: Figure 6—figure supplement 1—source data 1. [file elife-103996-fig6-figsupp1-data1.zip › elife-103996-fig6-figsupp1-data1-v1/Figure 6-figure supplement 1A V5.tif]

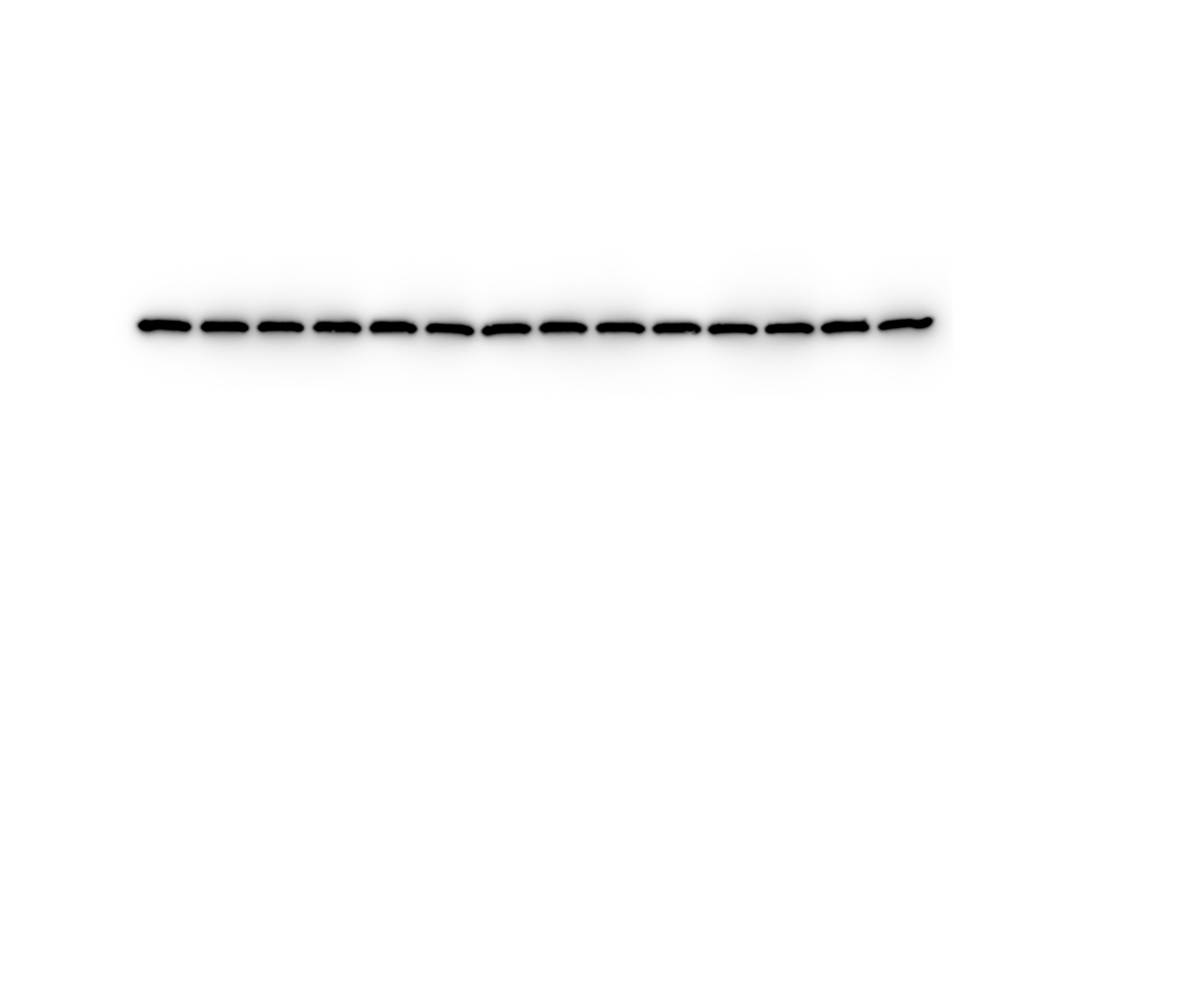

Supplement: Figure 6—figure supplement 1—source data 1. [file elife-103996-fig6-figsupp1-data1.zip › elife-103996-fig6-figsupp1-data1-v1/Figure 6-figure supplement 1B Actin.tif]

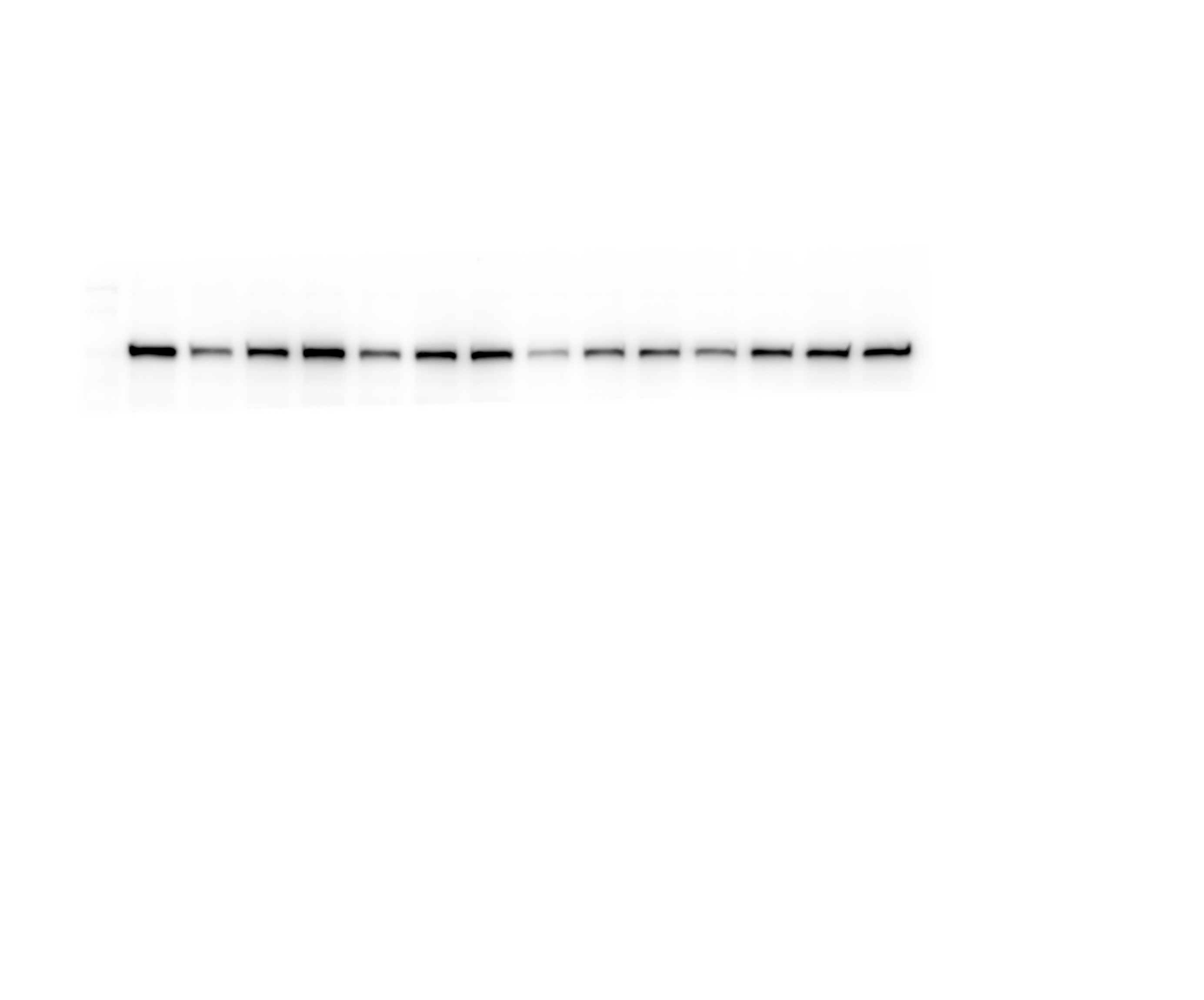

Supplement: Figure 6—figure supplement 1—source data 1. [file elife-103996-fig6-figsupp1-data1.zip › elife-103996-fig6-figsupp1-data1-v1/Figure 6-figure supplement 1B b-cat.tif]

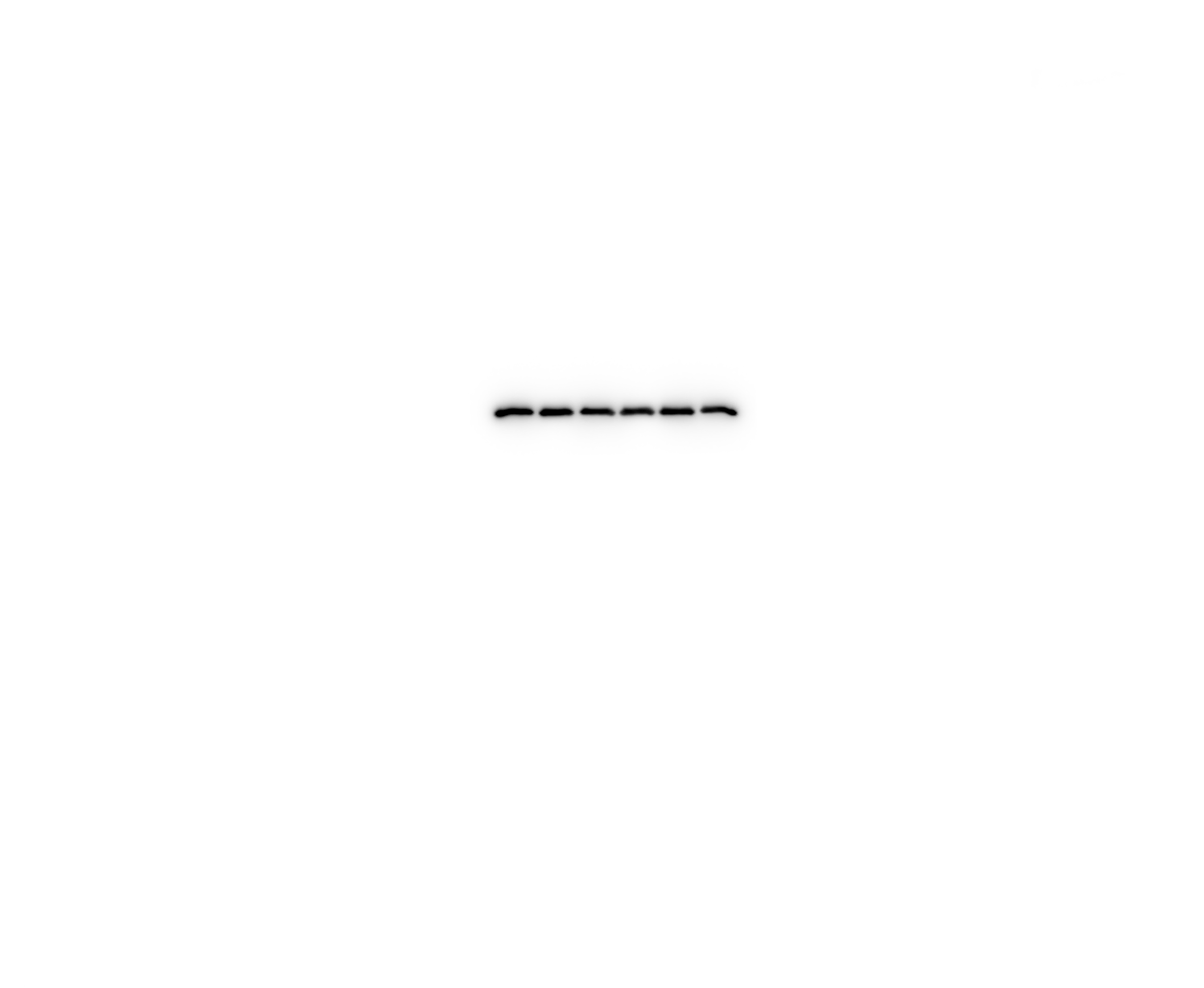

Supplement: Figure 7—source data 1. [file elife-103996-fig7-data1.zip › elife-103996-fig7-data1-v1/Figure 7A/Figure 7A Actin.tif]

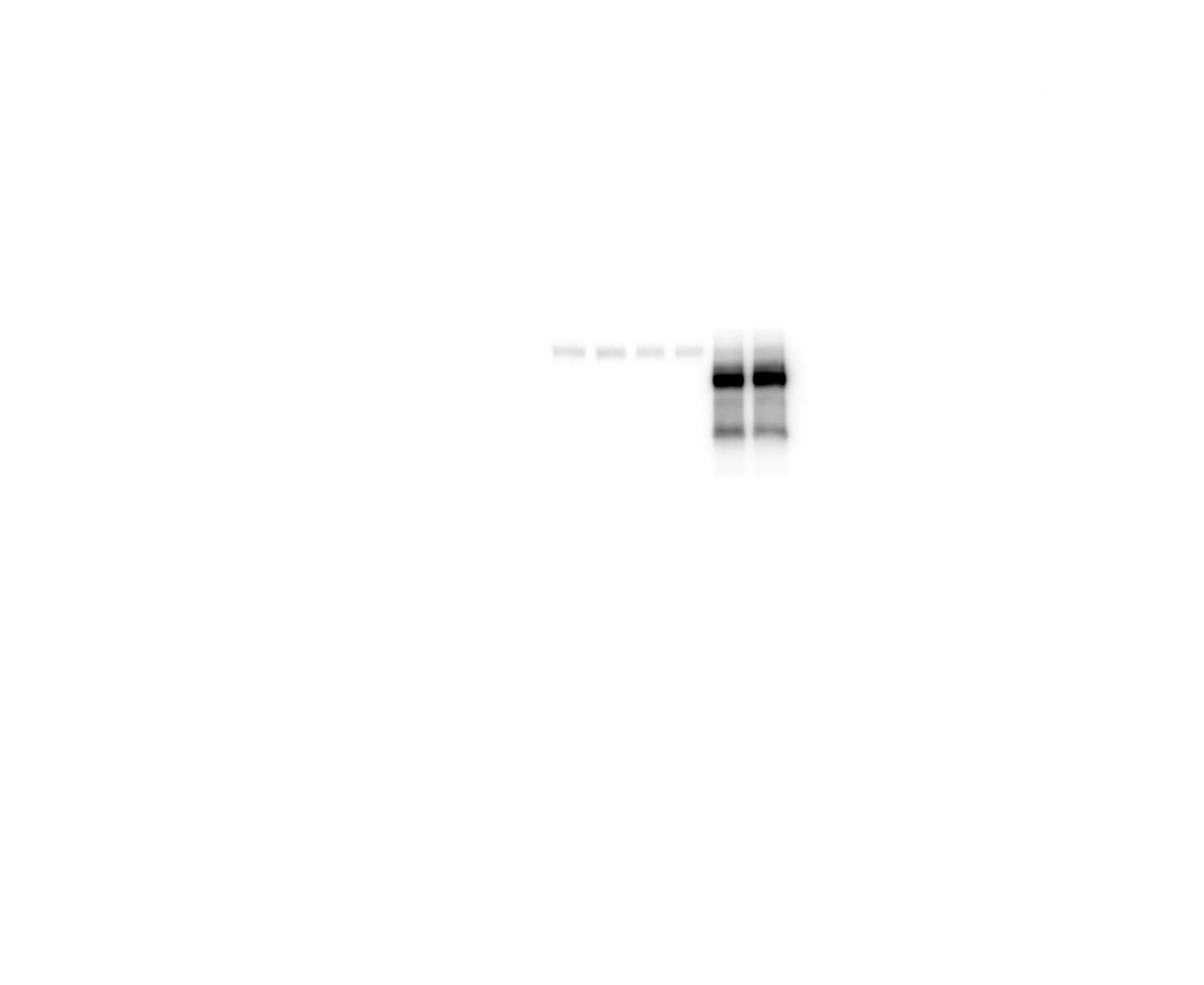

Supplement: Figure 7—source data 1. [file elife-103996-fig7-data1.zip › elife-103996-fig7-data1-v1/Figure 7A/Figure 7A HA.tif]

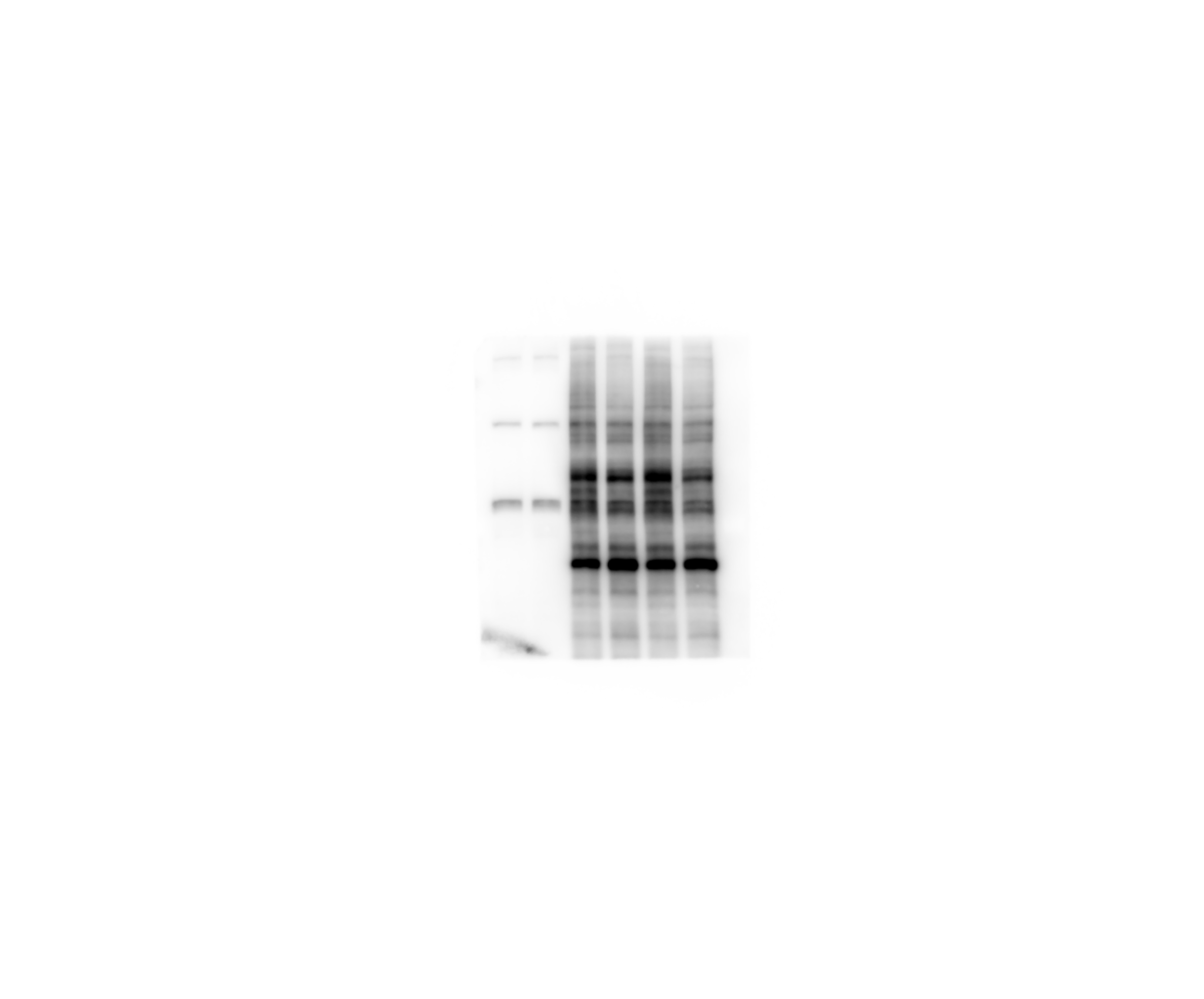

Supplement: Figure 7—source data 1. [file elife-103996-fig7-data1.zip › elife-103996-fig7-data1-v1/Figure 7A/Figure 7A StreptAvidin.tif]

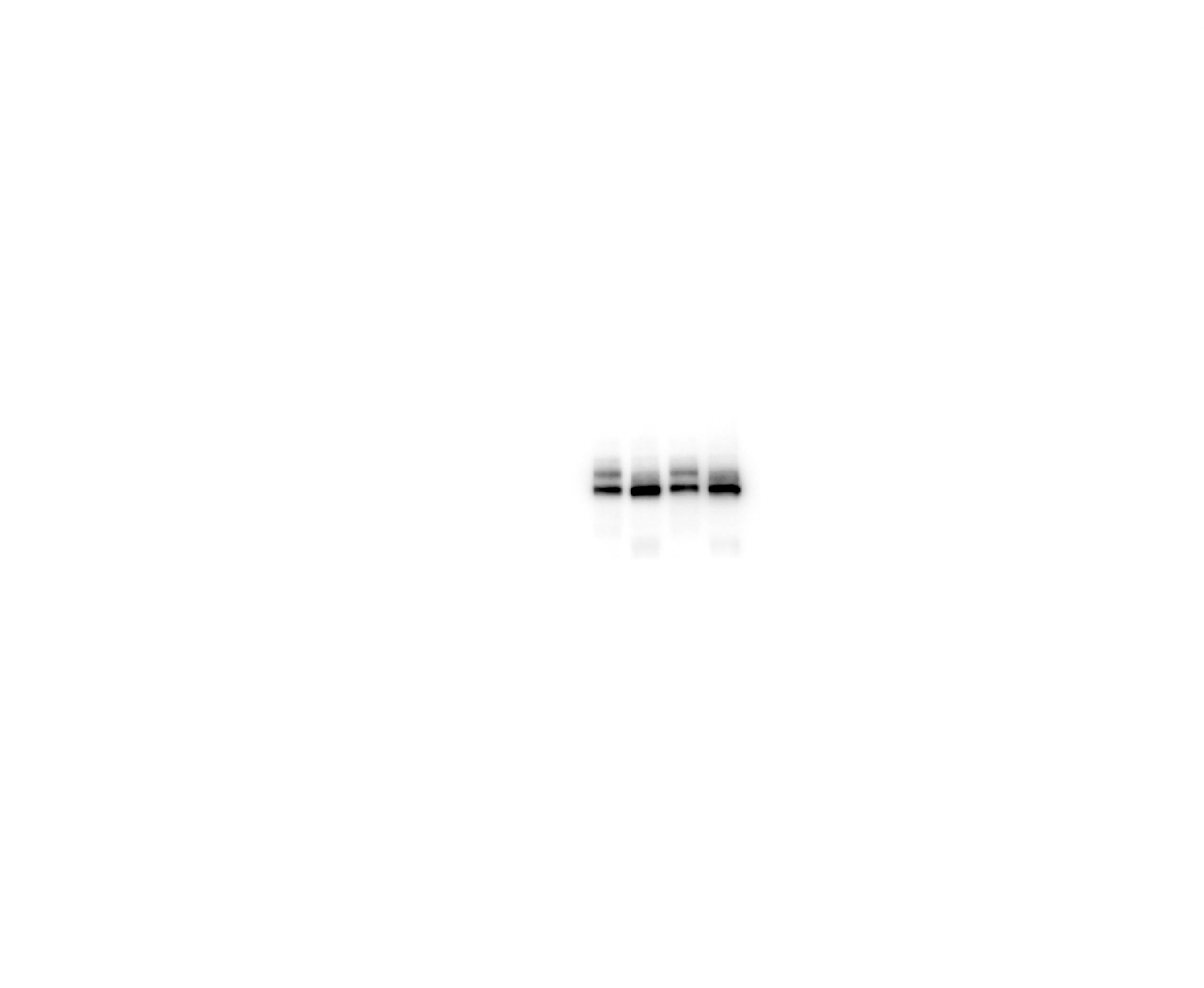

Supplement: Figure 7—source data 1. [file elife-103996-fig7-data1.zip › elife-103996-fig7-data1-v1/Figure 7A/Figure 7A V5.tif]

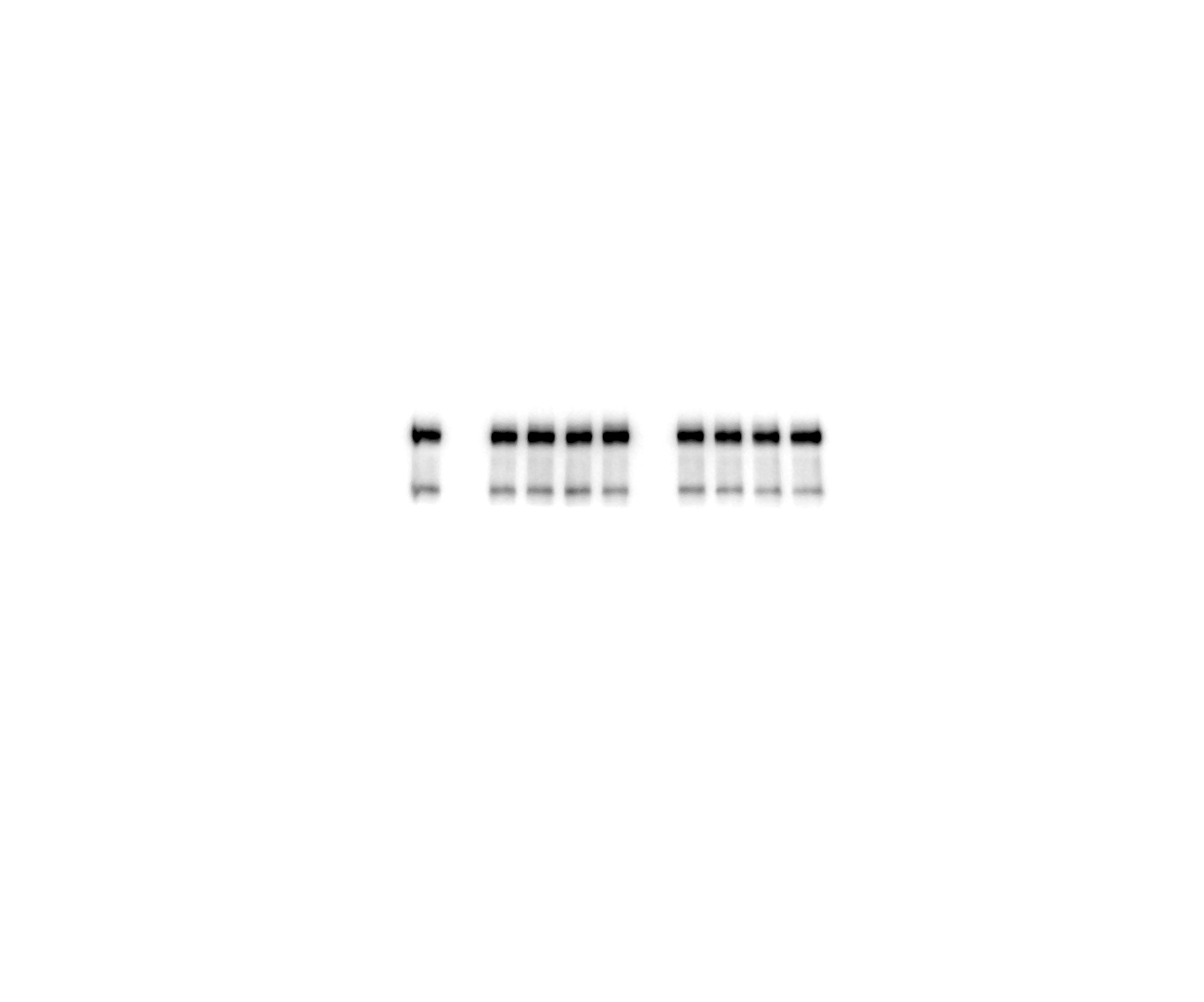

Supplement: Figure 7—source data 1. [file elife-103996-fig7-data1.zip › elife-103996-fig7-data1-v1/Figure 7B/Figure 7B Input HA.tif]

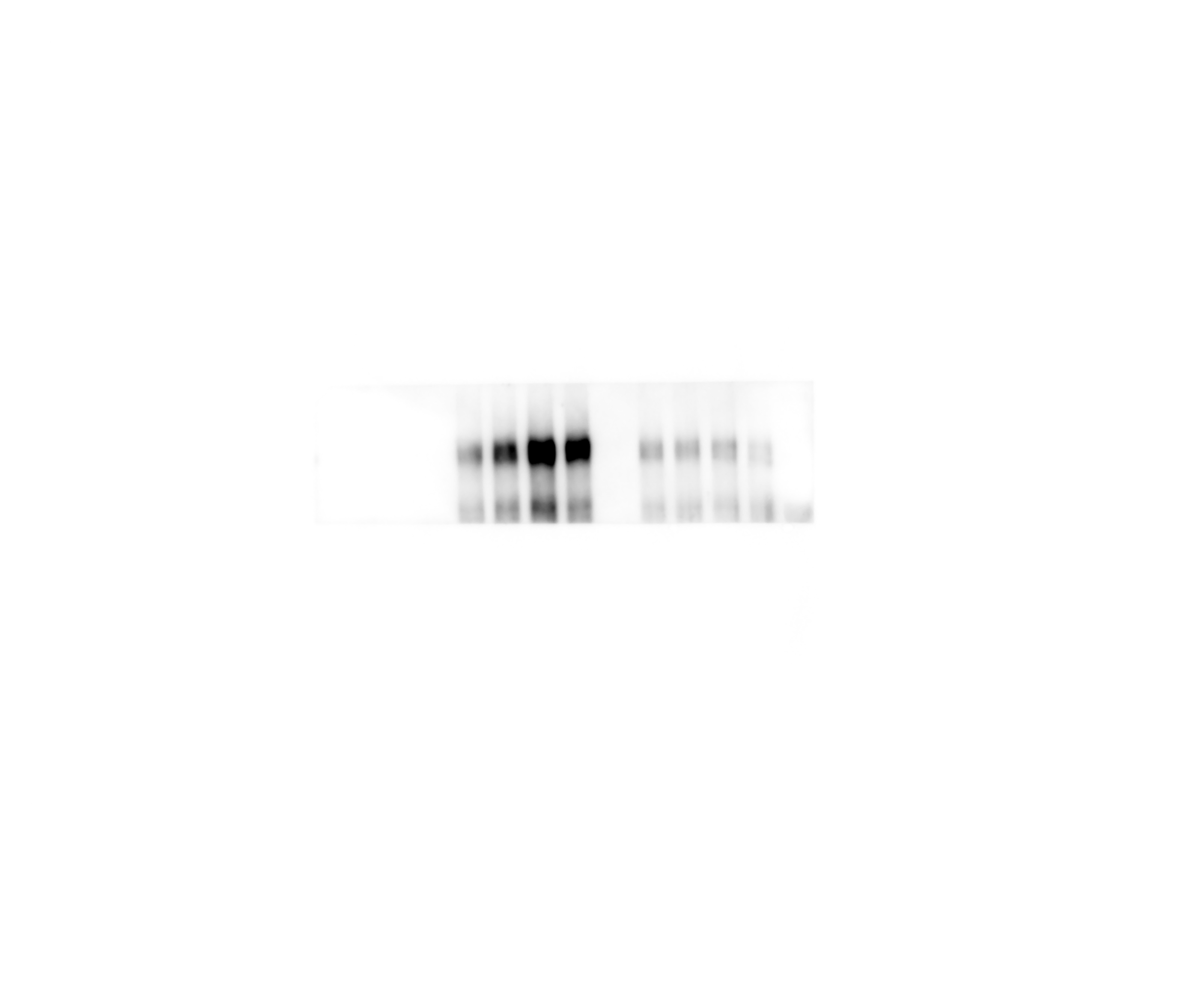

Supplement: Figure 7—source data 1. [file elife-103996-fig7-data1.zip › elife-103996-fig7-data1-v1/Figure 7B/Figure 7B IP HA.tif]

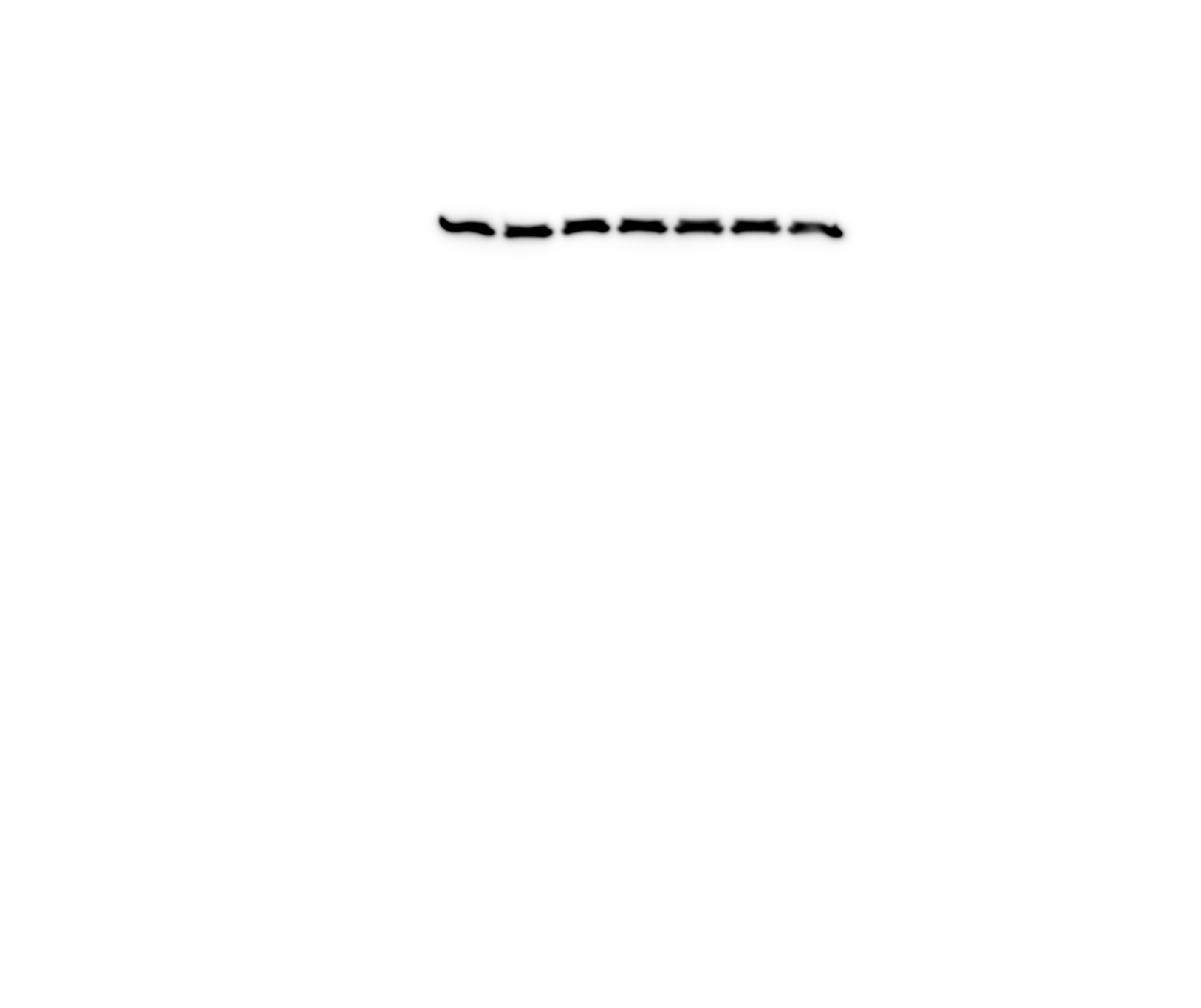

Supplement: Figure 7—figure supplement 1—source data 1. [file elife-103996-fig7-figsupp1-data1.zip › elife-103996-fig7-sigsupp1-data1-v1/Figure 7-figure supplement 1A/Figure 7-figure supplement 1A Actin.tif]

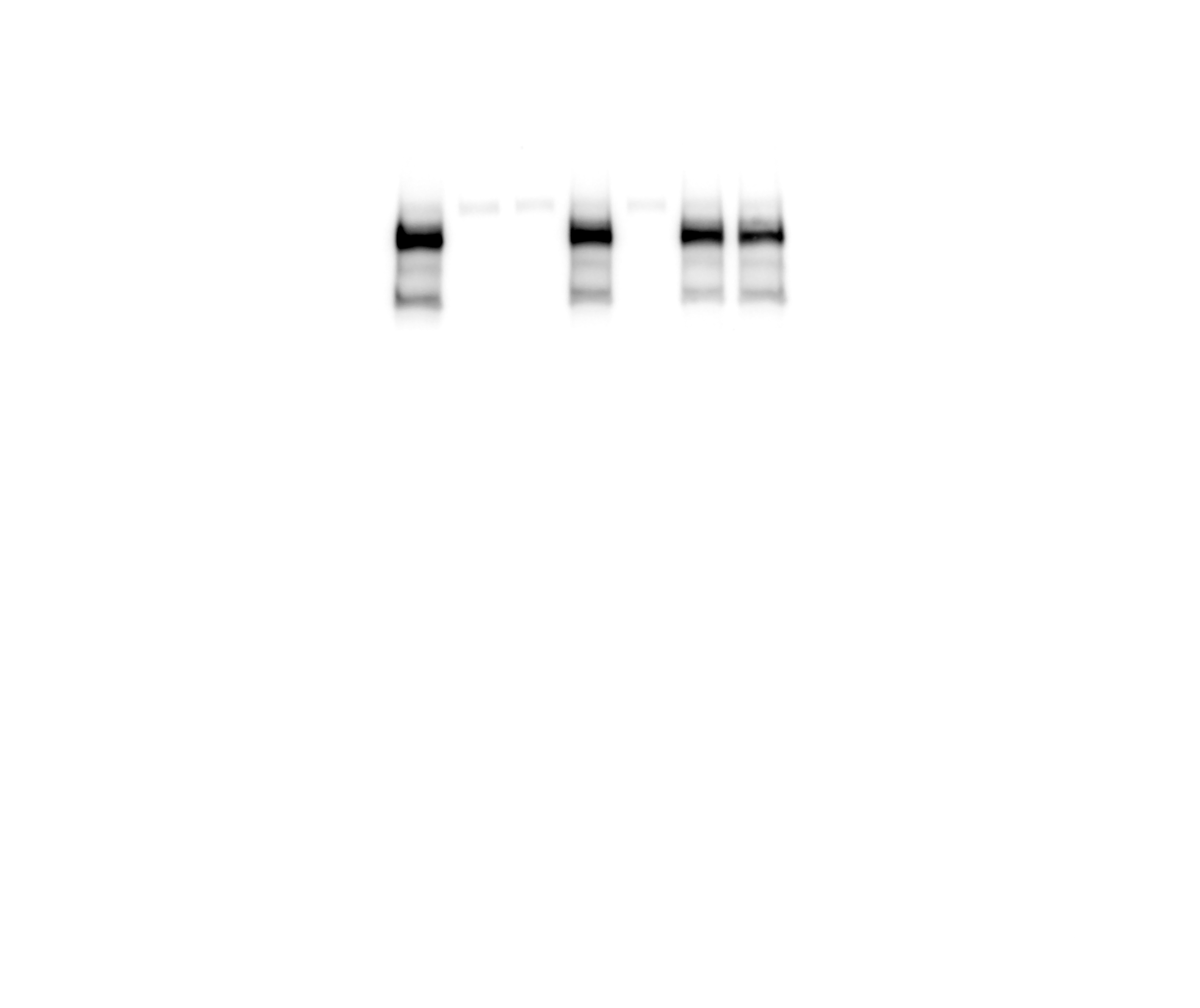

Supplement: Figure 7—figure supplement 1—source data 1. [file elife-103996-fig7-figsupp1-data1.zip › elife-103996-fig7-sigsupp1-data1-v1/Figure 7-figure supplement 1A/Figure 7-figure supplement 1A HA.tif]

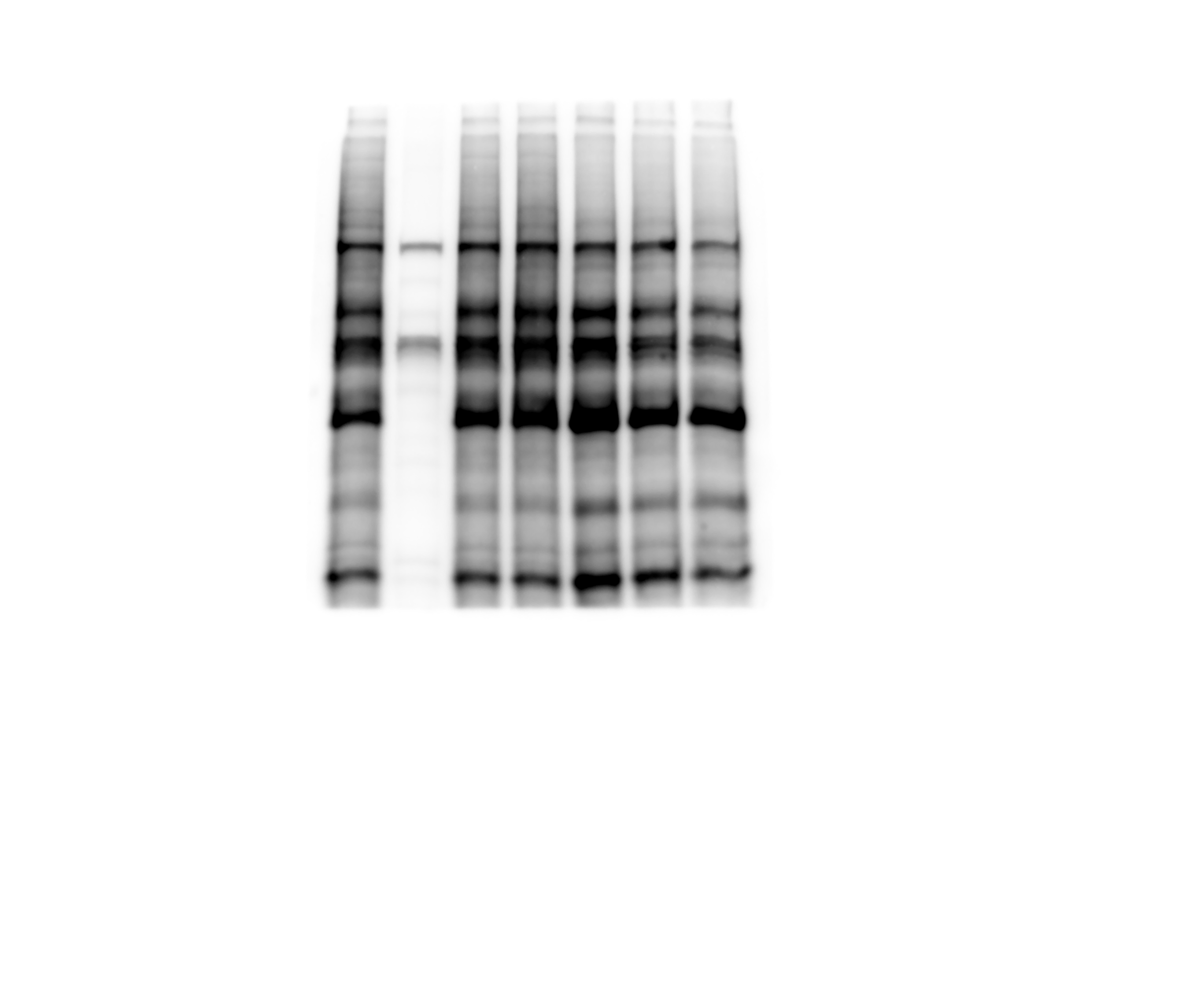

Supplement: Figure 7—figure supplement 1—source data 1. [file elife-103996-fig7-figsupp1-data1.zip › elife-103996-fig7-sigsupp1-data1-v1/Figure 7-figure supplement 1A/Figure 7-figure supplement 1A StreptAvidin.tif]

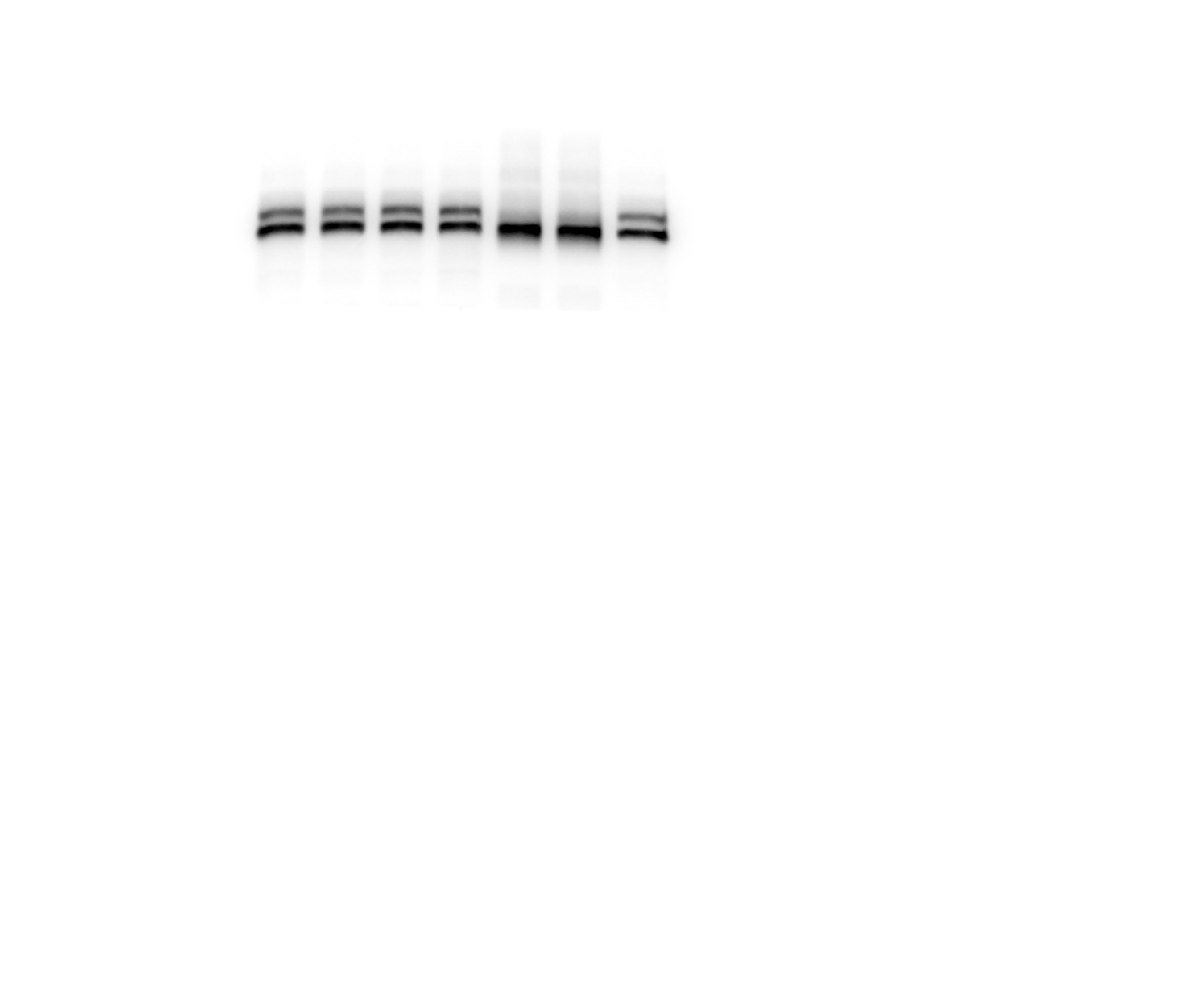

Supplement: Figure 7—figure supplement 1—source data 1. [file elife-103996-fig7-figsupp1-data1.zip › elife-103996-fig7-sigsupp1-data1-v1/Figure 7-figure supplement 1A/Figure 7-figure supplement 1A V5.tif]

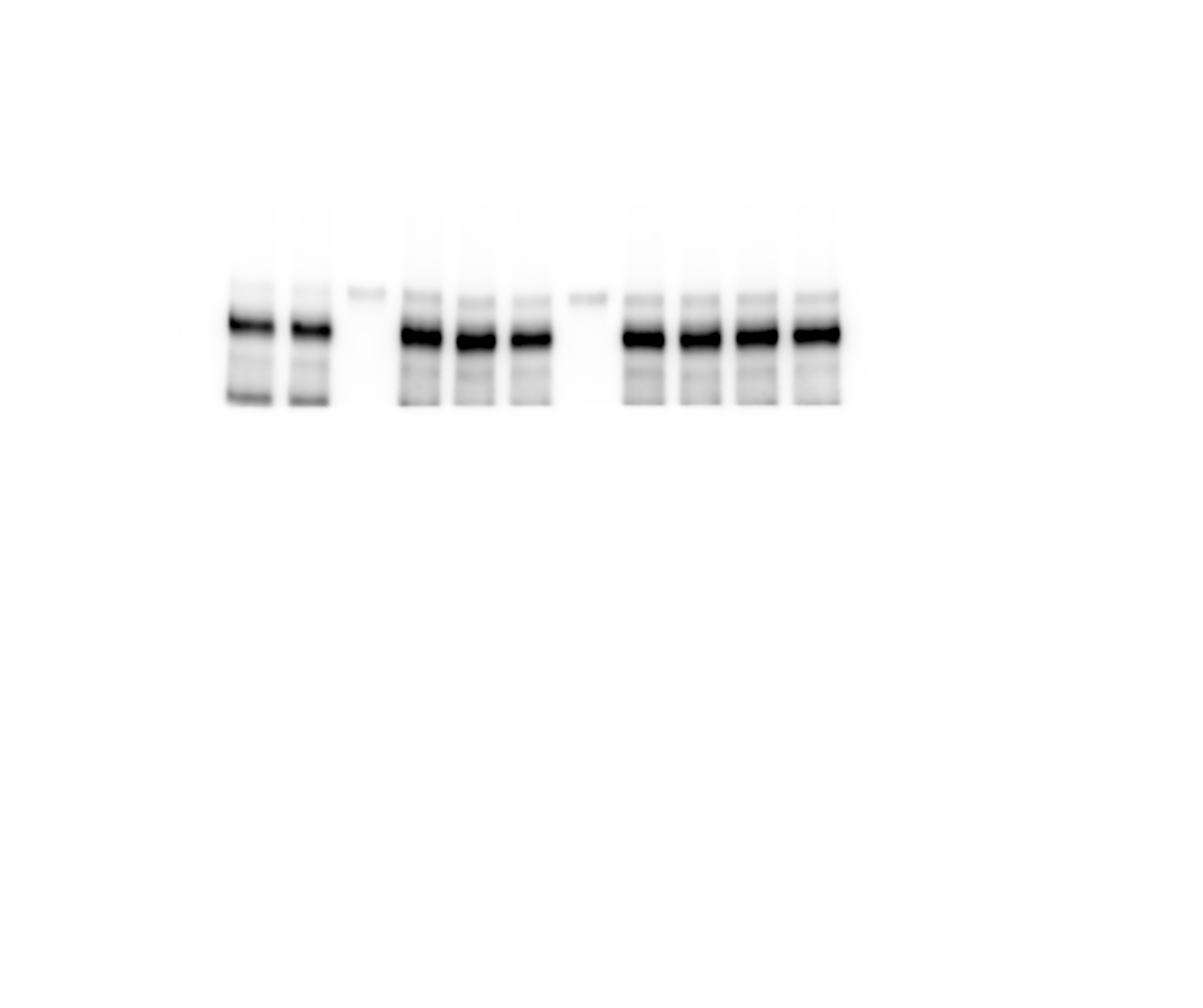

Supplement: Figure 7—figure supplement 1—source data 1. [file elife-103996-fig7-figsupp1-data1.zip › elife-103996-fig7-sigsupp1-data1-v1/Figure 7-figure supplement 1B/Figure 7-figure supplement 1B Input HA.tif]

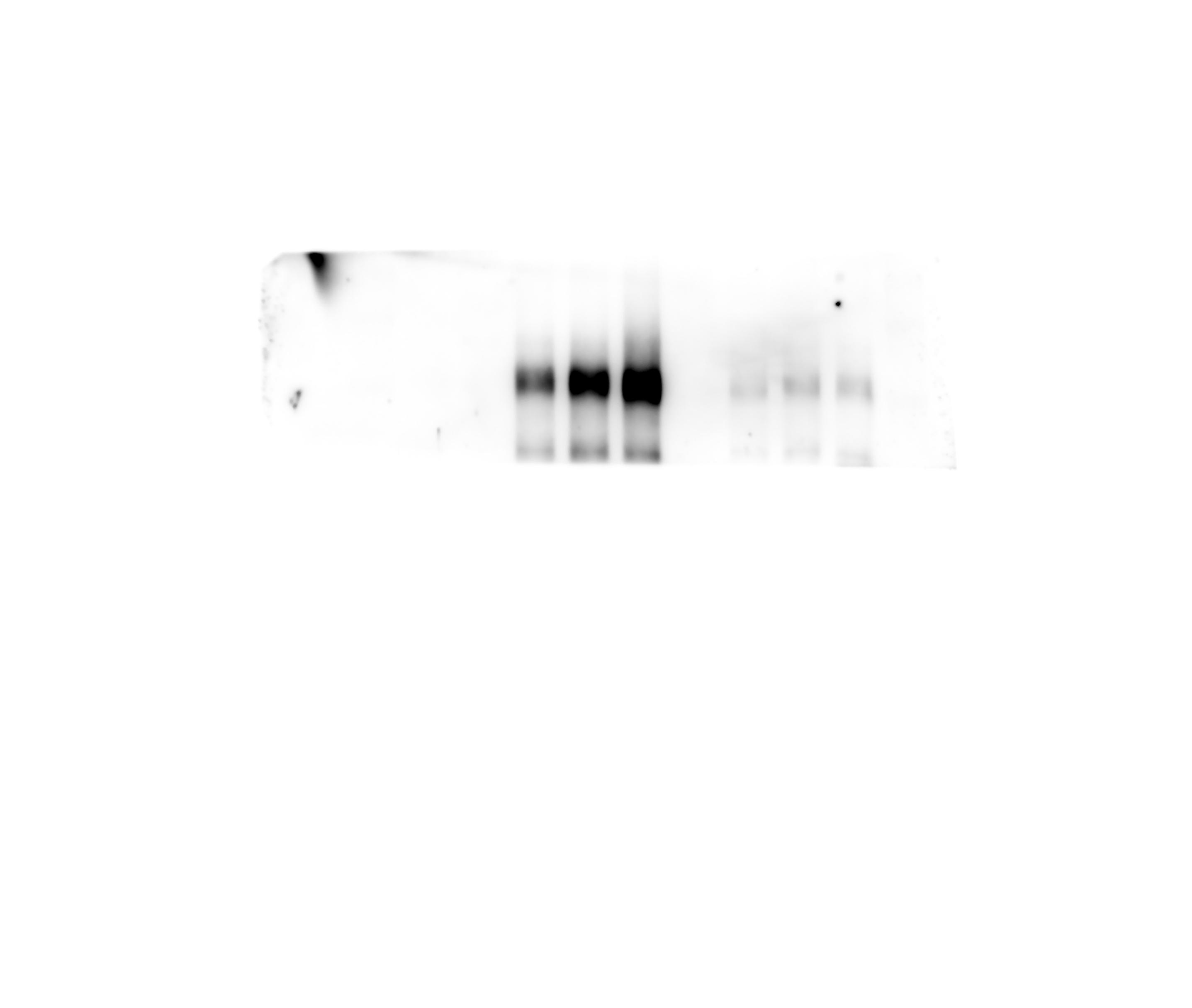

Supplement: Figure 7—figure supplement 1—source data 1. [file elife-103996-fig7-figsupp1-data1.zip › elife-103996-fig7-sigsupp1-data1-v1/Figure 7-figure supplement 1B/Figure 7-figure supplement 1B IP HA.tif]
