# Supplementary material for: Wnt induces FZD5/8 endocytosis and degradation and the involvement of RSPO-ZNRF3/RNF43 and DVL
Source: eLife. 2025 Oct 10;14:RP103996. doi: 10.7554/eLife.103996 (PMC12513720; doi:10.7554/eLife.103996)

Figure 1-source data

Figure 1B:
V5(FZD1-3)

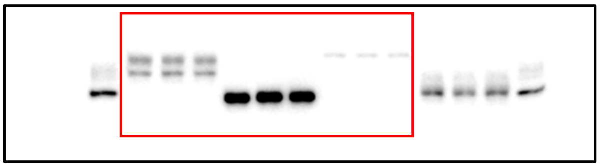

Figure 1B:
Actin(FZD5/6)

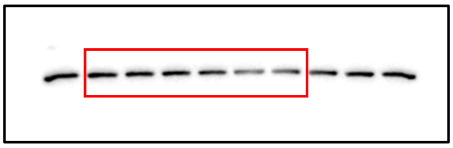

Figure 1B:
V5(FZD4/7)

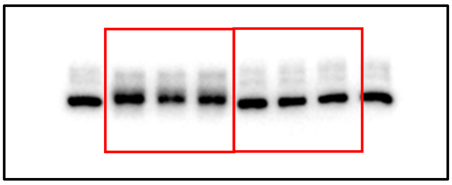

Figure 1B:
Actin(FZD9/10)

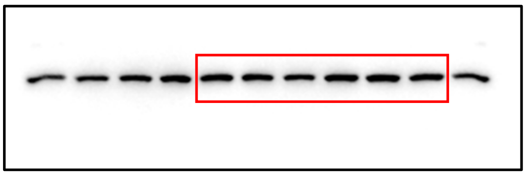

Figure 1B:
V5(FZD5/6)

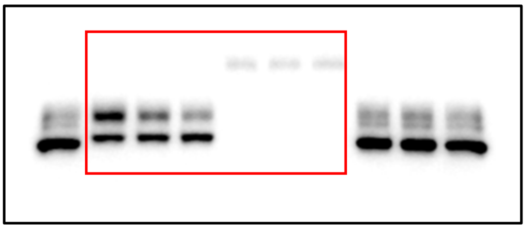

Figure 1B:
Actin(FZD1-3)

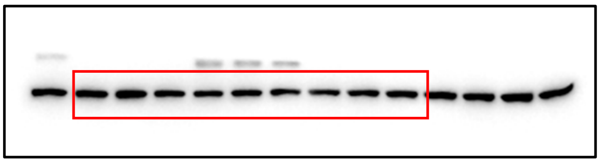

Figure 1B:
V5(FZD8)

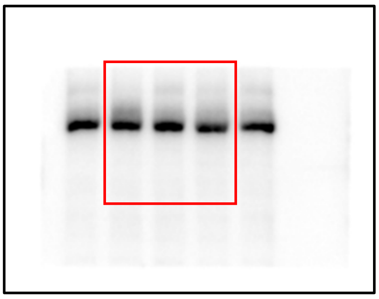

Figure 1B:
Actin(FZD4/7/8)

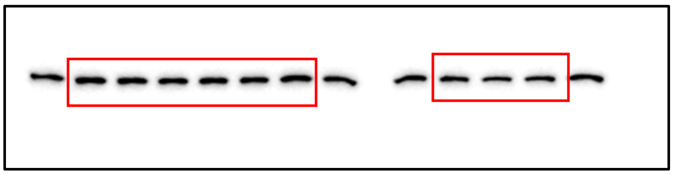

Figure 1B:
V5(FZD9/10)

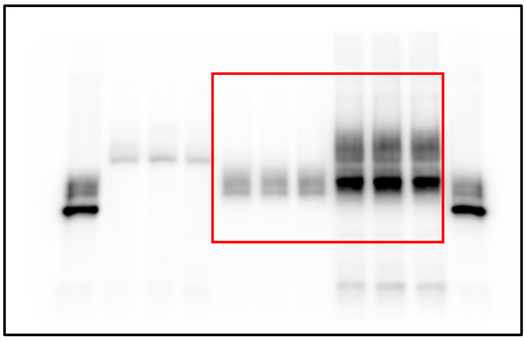

Figure 1C:
V5

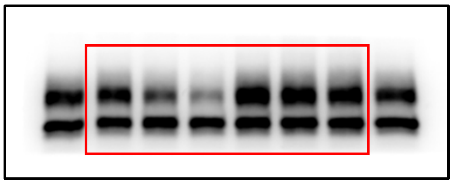

Figure 1C:
Actin

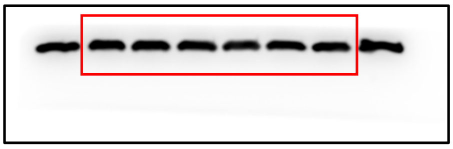

Supplement: Figure 1—source data 2. [file elife-103996-fig1-data2.zip › elife-103996-fig1-data2-v1.pdf]
