# Supplementary material for: Wnt induces FZD5/8 endocytosis and degradation and the involvement of RSPO-ZNRF3/RNF43 and DVL
Source: eLife. 2025 Oct 10;14:RP103996. doi: 10.7554/eLife.103996 (PMC12513720; doi:10.7554/eLife.103996)

Figure 1-figure supplement 1-source data

Figure 1-figure supplement 1B:
V5(FZD1/4)

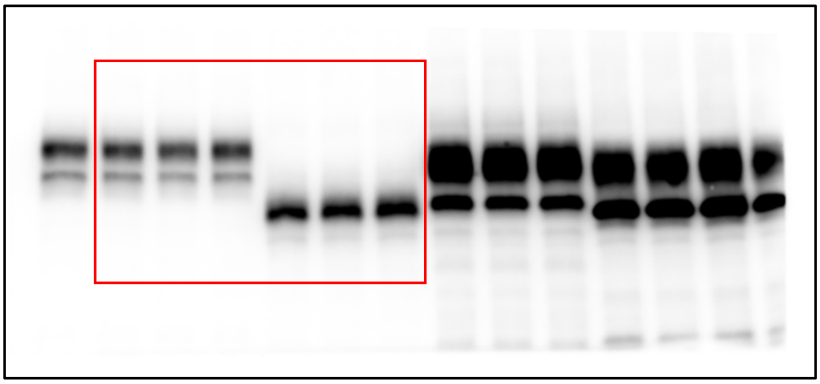

Figure 1-figure supplement 1B:
Actin(FZD1/4)

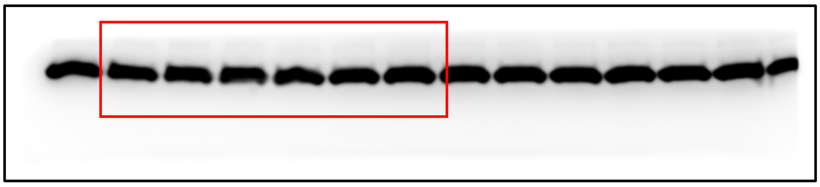

Figure 1-figure supplement 1C:
V5(FZD5)

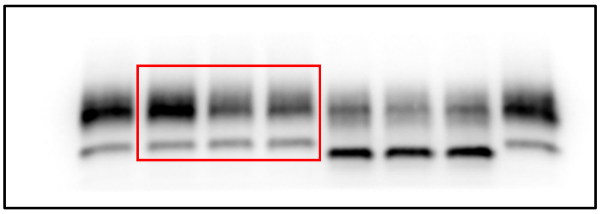

Figure 1-figure supplement 1C:
Actin(FZD5)

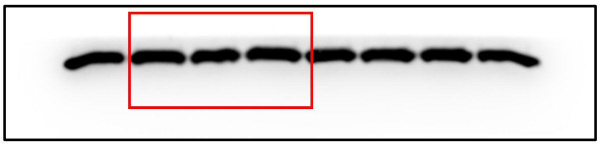

Figure 1-figure supplement 1D:
V5(FZD7)

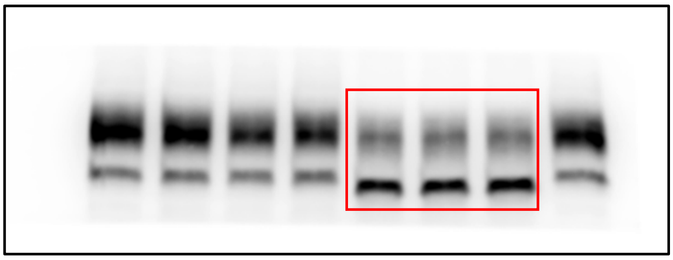

Figure 1-figure supplement 1D:
Actin(FZD7)

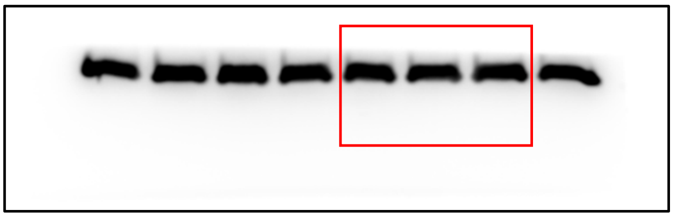

Supplement: Figure 1—figure supplement 1—source data 2. [file elife-103996-fig1-figsupp1-data2.zip › elife-103996-fig1-figsupp1-data2-v1.pdf]
