# Supplementary material for: Wnt induces FZD5/8 endocytosis and degradation and the involvement of RSPO-ZNRF3/RNF43 and DVL
Source: eLife. 2025 Oct 10;14:RP103996. doi: 10.7554/eLife.103996 (PMC12513720; doi:10.7554/eLife.103996)

Figure 2-source data

Figure 2A:
V5

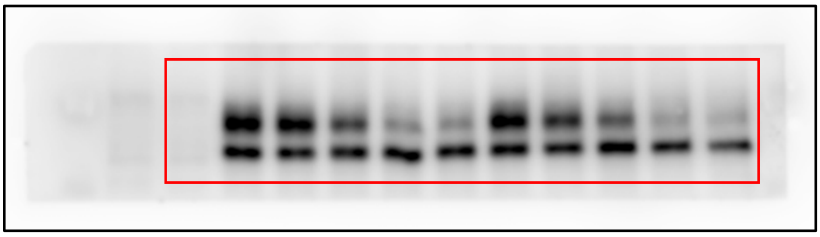

Figure 2A:
Actin

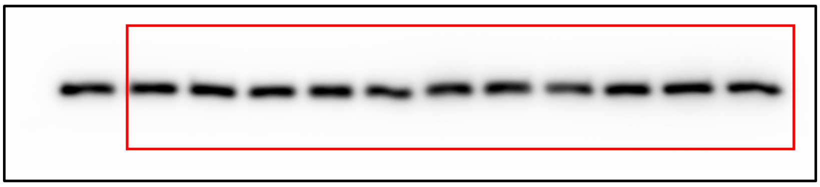

Figure 2B:
V5

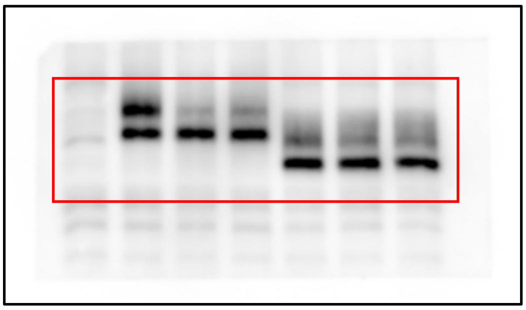

Figure 2B:
Actin

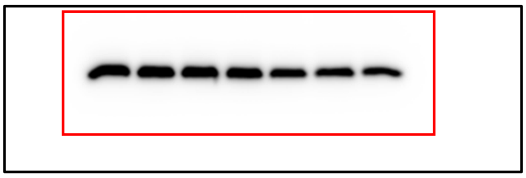

Figure 2C:
V5

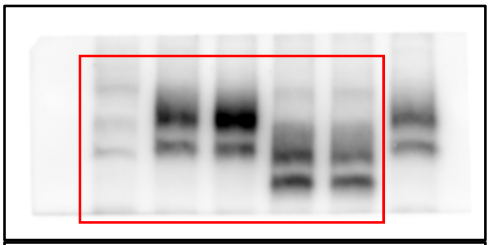

Figure 2C:
Actin

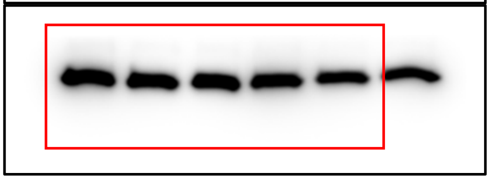

Figure 2H:
V5

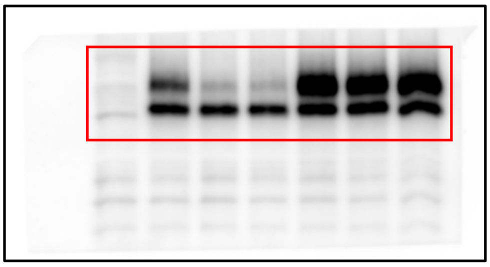

Figure 2H:
Actin

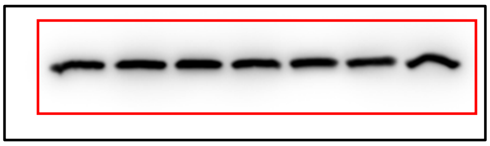

Figure 2I:
V5

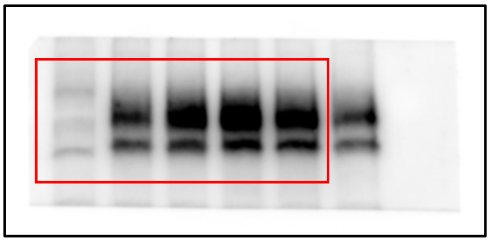

Figure 2I:
Actin

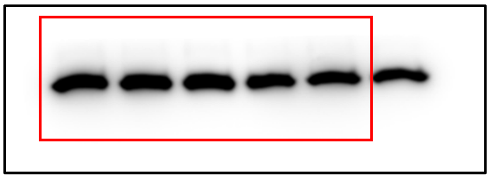

Figure 2J:
V5

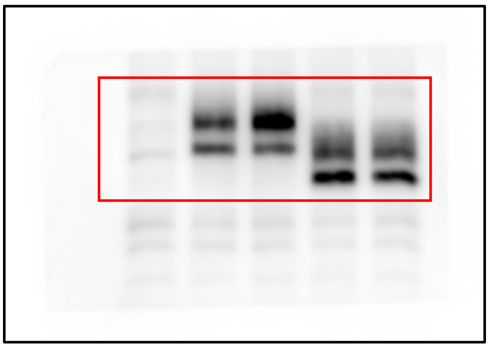

Figure 2J:
Actin

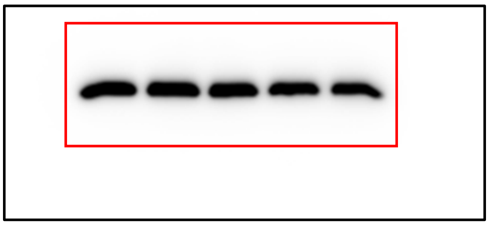

Figure 2K:
V5

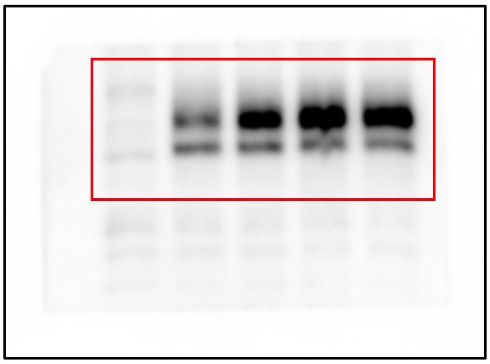

Figure 2K:
Actin

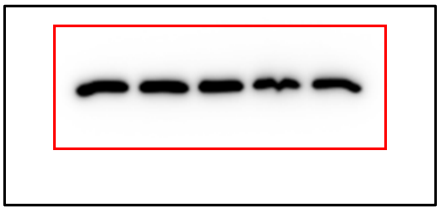

Figure 2L:
V5

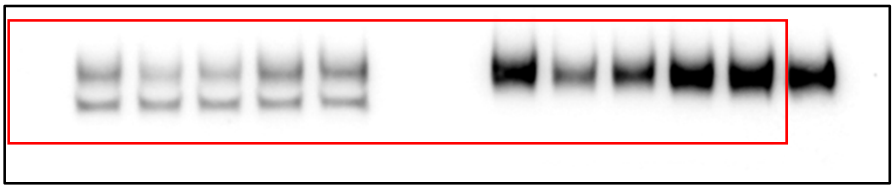

Figure 2L:
Actin

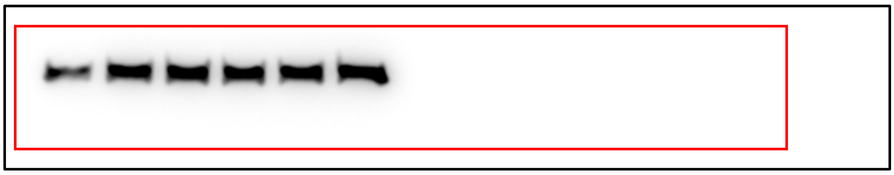

Supplement: Figure 2—source data 2. [file elife-103996-fig2-data2.zip › elife-103996-fig2-data2-v1.pdf]
