# Supplementary material for: Wnt induces FZD5/8 endocytosis and degradation and the involvement of RSPO-ZNRF3/RNF43 and DVL
Source: eLife. 2025 Oct 10;14:RP103996. doi: 10.7554/eLife.103996 (PMC12513720; doi:10.7554/eLife.103996)

Figure 3-source data

Figure 3A:
DVL1

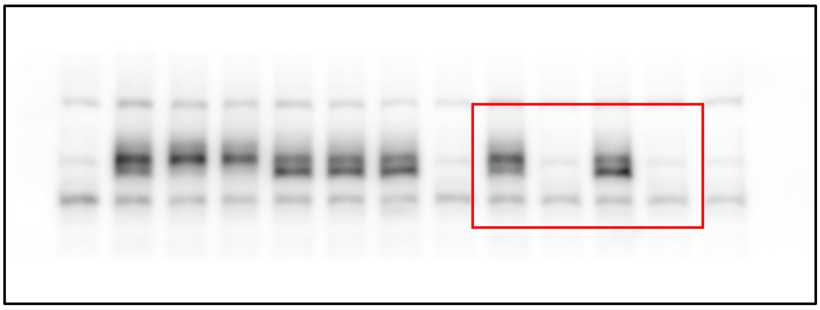

Figure 3A:
DVL2

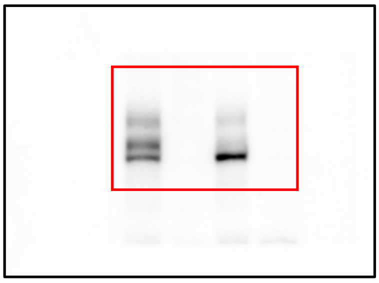

Figure 3A:
DVL3

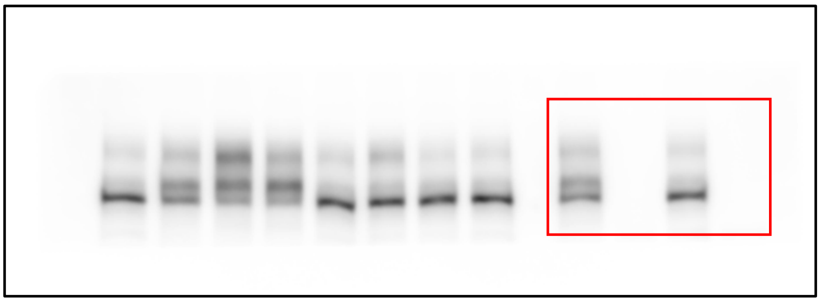

Figure 3F:
V5

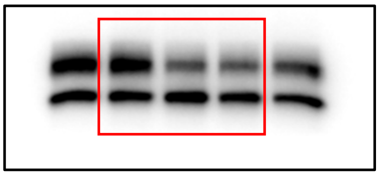

Figure 3F:
Actin

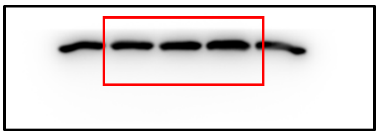

Figure 3K:
V5

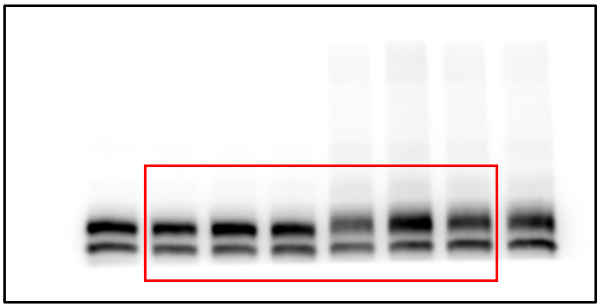

Figure 3K:
DVL2

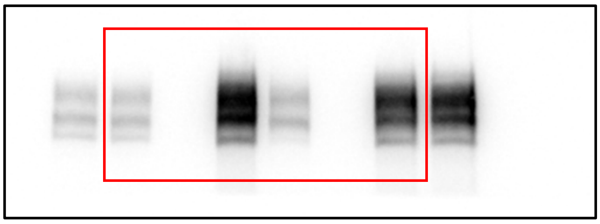

Figure 3K:
Actin

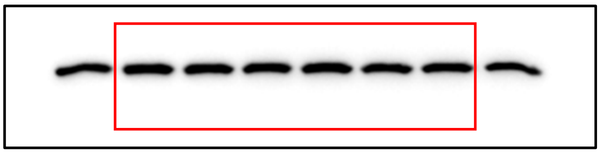

Supplement: Figure 3—source data 2. [file elife-103996-fig3-data2.zip › elife-103996-fig3-data2-v1.pdf]
