# Supplementary material for: Wnt induces FZD5/8 endocytosis and degradation and the involvement of RSPO-ZNRF3/RNF43 and DVL
Source: eLife. 2025 Oct 10;14:RP103996. doi: 10.7554/eLife.103996 (PMC12513720; doi:10.7554/eLife.103996)

Figure 5-source data

Figure 5B:
V5

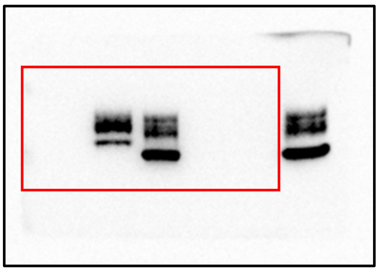

Figure 5B:
β-catenin

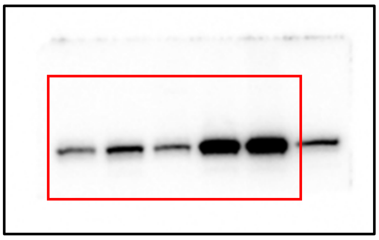

Figure 5B:
Actin

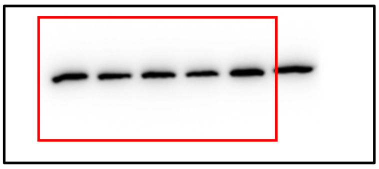

Figure 5C:
DVL1

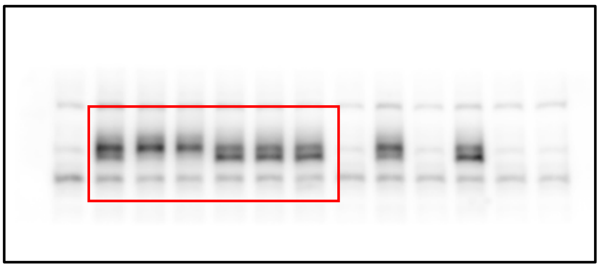

Figure 5C:
DVL2

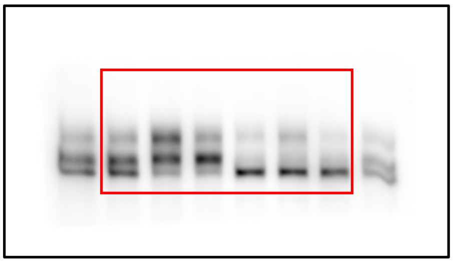

Figure 5C:
DVL3

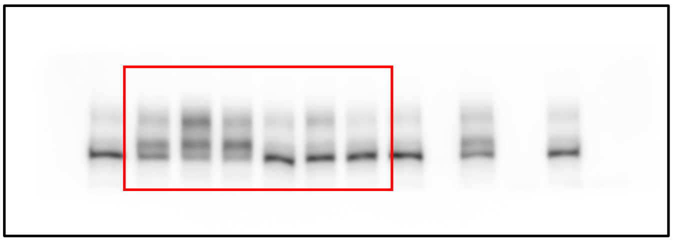

Figure 5C:
β-catenin

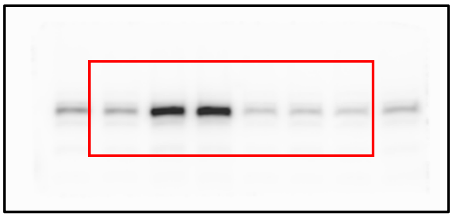

Figure 5C:
Actin

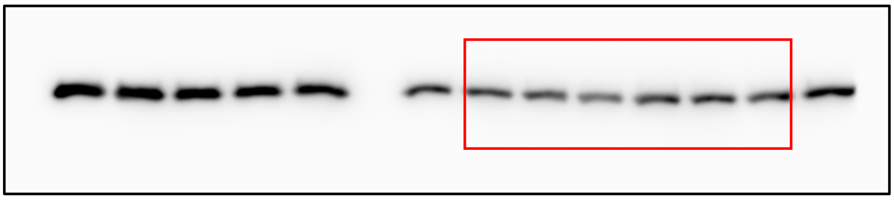

Figure 5D:
DVL1

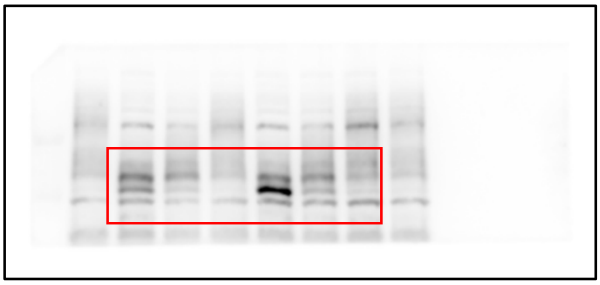

Figure 5D:
DVL2

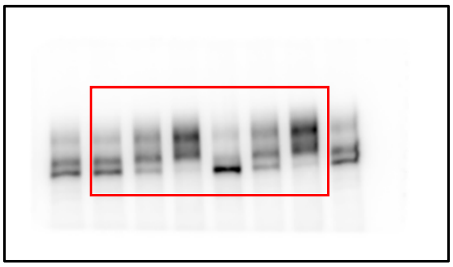

Figure 5D:
DVL3

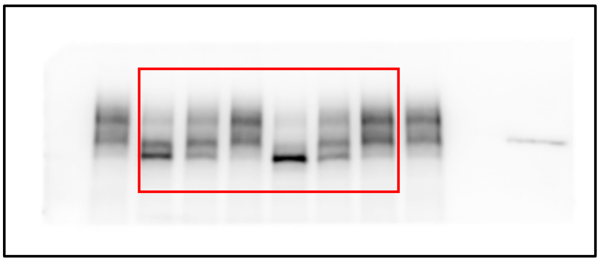

Figure 5D:
V5

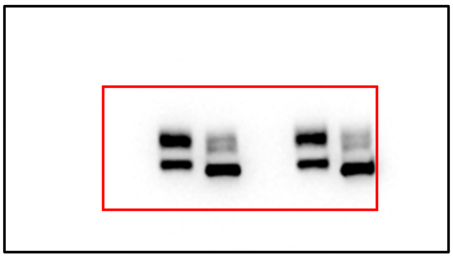

Figure 5D:
Actin

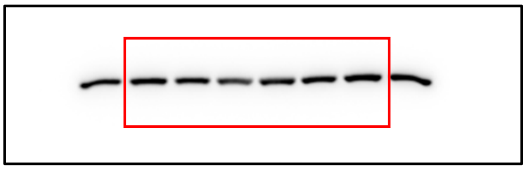

Figure 5E:
HA

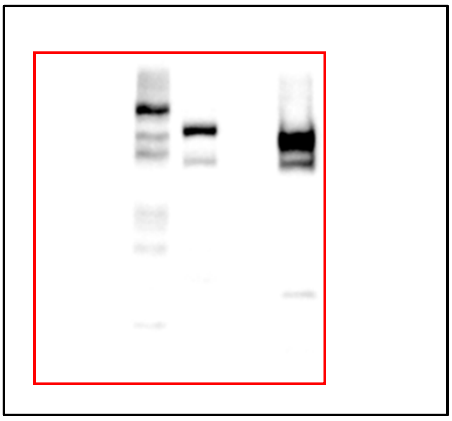

Figure 5E:
HA
(darker exposure)

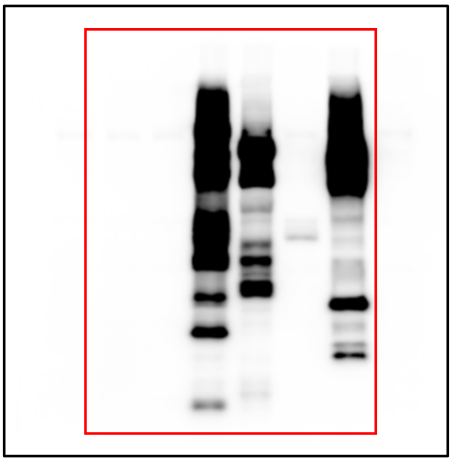

Figure 5E:
β-catenin

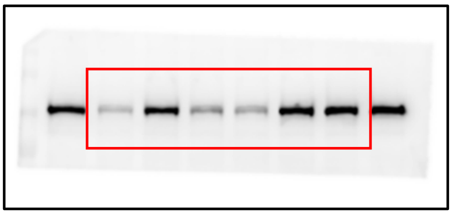

Figure 5E:
Actin

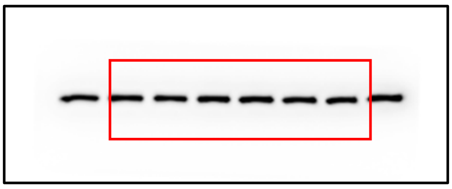

Figure 5F:
V5

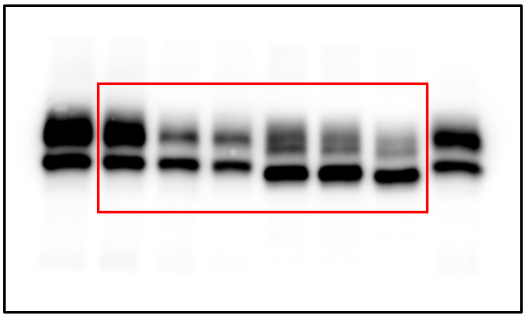

Figure 5F:
HA

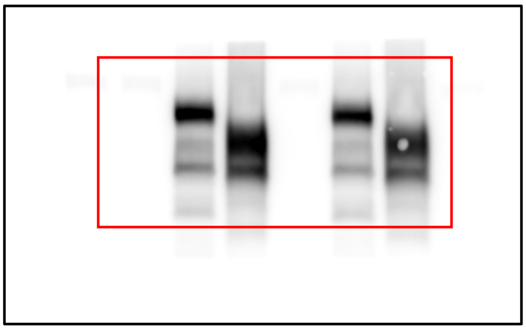

Figure 5E:
Actin

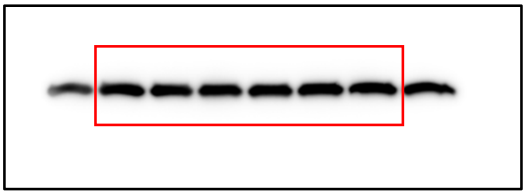

Supplement: Figure 5—source data 2. [file elife-103996-fig5-data2.zip › elife-103996-fig5-data2-v1.pdf]
