# Supplementary material for: Wnt induces FZD5/8 endocytosis and degradation and the involvement of RSPO-ZNRF3/RNF43 and DVL
Source: eLife. 2025 Oct 10;14:RP103996. doi: 10.7554/eLife.103996 (PMC12513720; doi:10.7554/eLife.103996)

Figure 6-source data

Figure 6B:
β-catenin

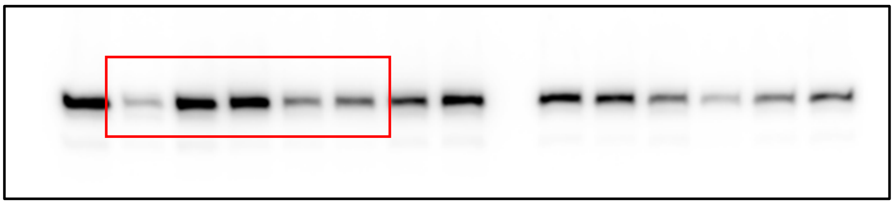

Figure 6B:
Actin

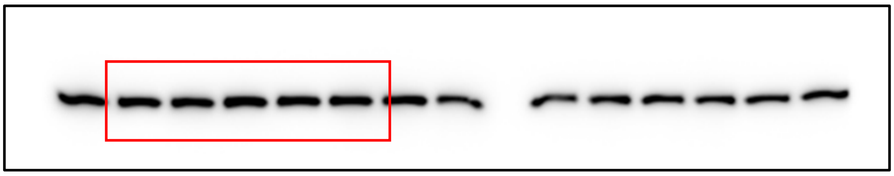

Figure 6C:
β-catenin

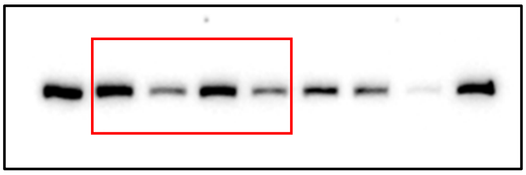

Figure 6C:
V5

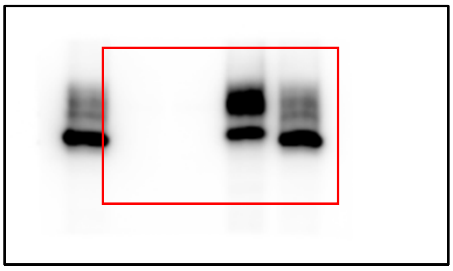

Figure 6C:
Actin

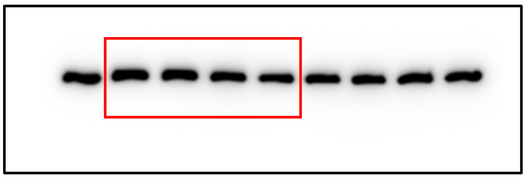

Figure 6D:
β-catenin

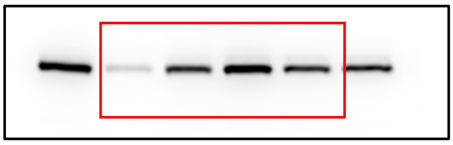

Figure 6D:
V5

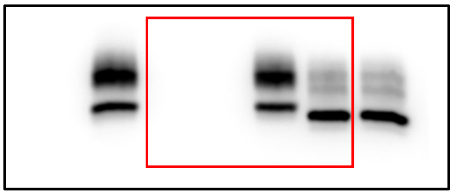

Figure 6D:
Actin

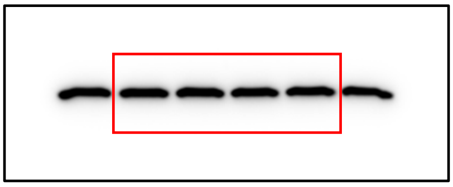

Figure 6E:
HA

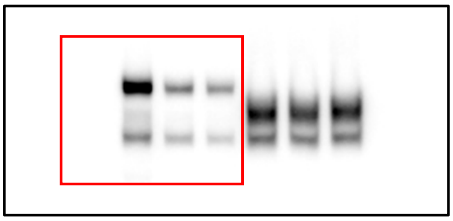

Figure 6E:
V5

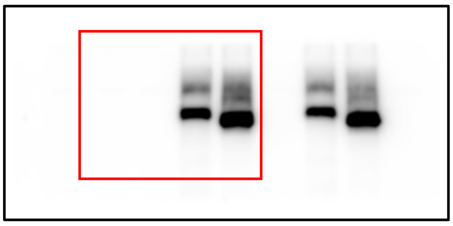

Figure 6E:
Actin

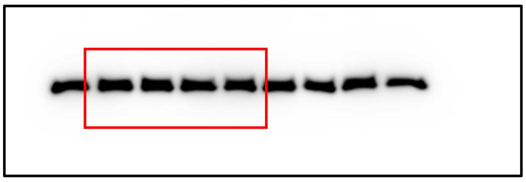

Figure 6F:
β-catenin

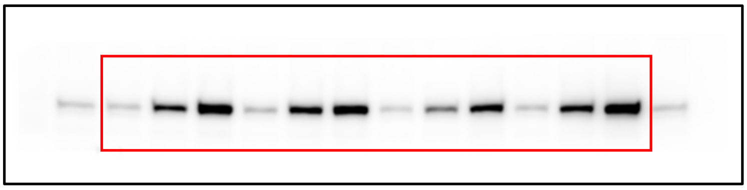

Figure 6F:
Actin

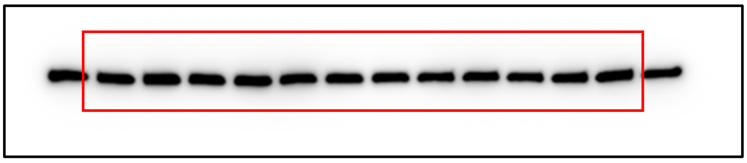

Figure 6H:
β-catenin

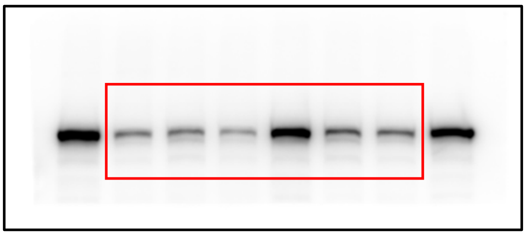

Figure 6H:
Actin

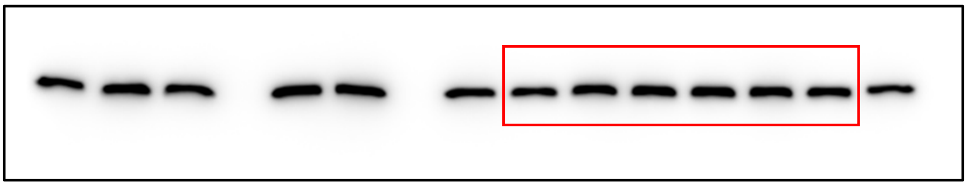

Figure 6I:
β-catenin

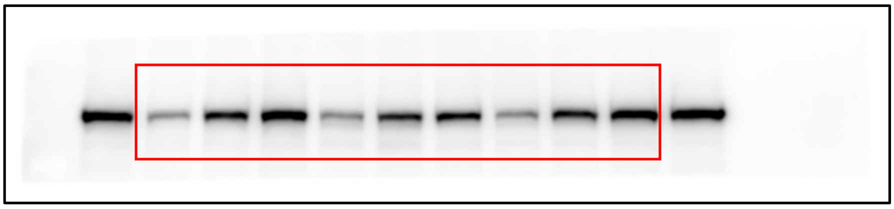

Figure 6I:
Actin

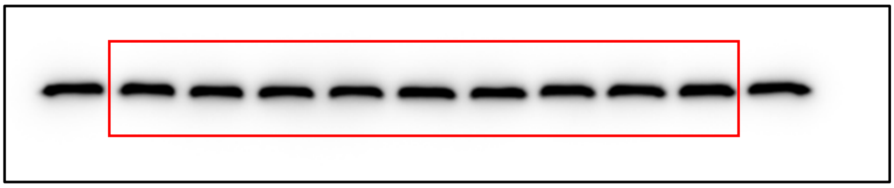

Figure 6J:
V5

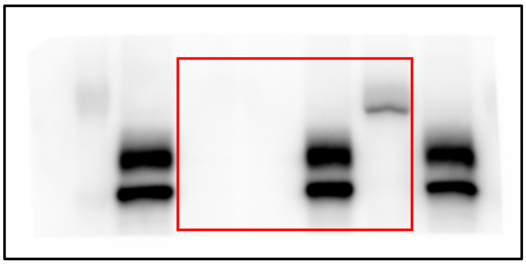

Figure 6J:
Actin

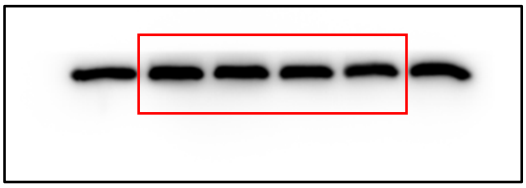

Figure 6K:
β-catenin

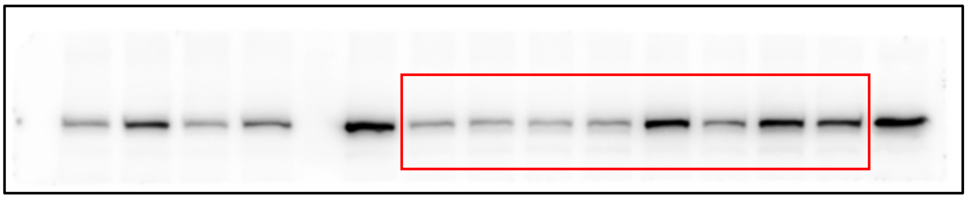

Figure 6K:
Actin

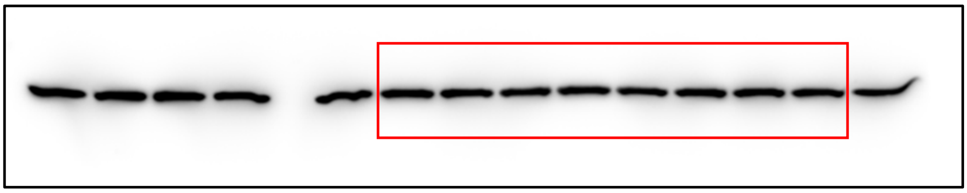

Supplement: Figure 6—source data 2. [file elife-103996-fig6-data2.zip › elife-103996-fig6-data2-v1.pdf]
