# Supplementary material for: Wnt induces FZD5/8 endocytosis and degradation and the involvement of RSPO-ZNRF3/RNF43 and DVL
Source: eLife. 2025 Oct 10;14:RP103996. doi: 10.7554/eLife.103996 (PMC12513720; doi:10.7554/eLife.103996)
Supplement: Figure 6—figure supplement 1—source data 2. [file elife-103996-fig6-figsupp1-data2.zip › elife-103996-fig6-figsupp1-data2-v1.pdf]

Figure 6-figure supplement 1-source data

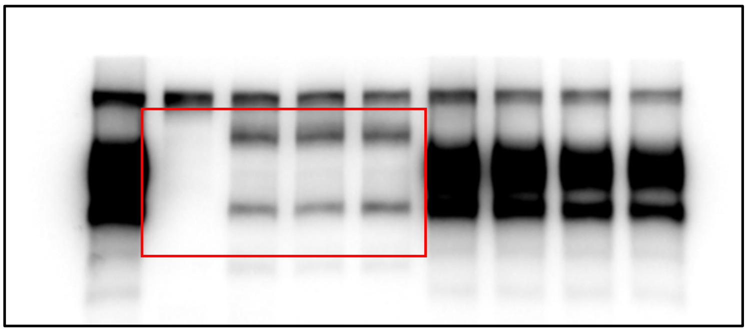

Figure 6-figure supplement 1A: HA

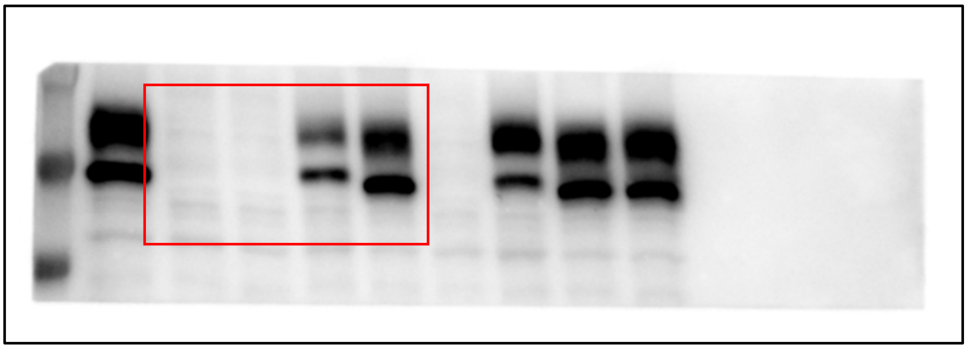

Figure 6-figure supplement 1A: V5

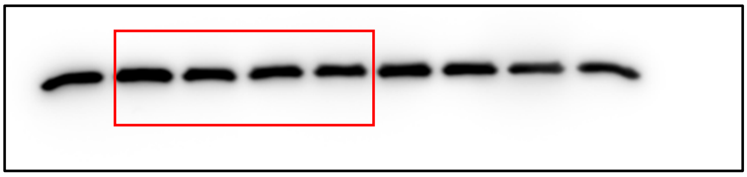

Figure 6-figure supplement 1A: Actin

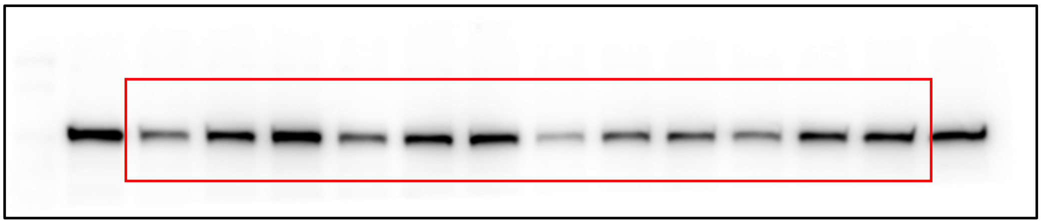

Figure 6-figure supplement 1B: β-catenin

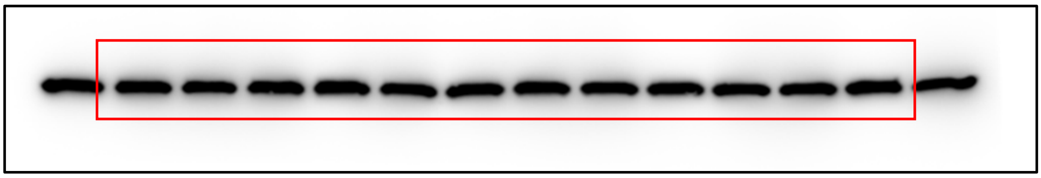

Figure 6-figure supplement 1B: Actin
