# Supplementary material for: Wnt induces FZD5/8 endocytosis and degradation and the involvement of RSPO-ZNRF3/RNF43 and DVL
Source: eLife. 2025 Oct 10;14:RP103996. doi: 10.7554/eLife.103996 (PMC12513720; doi:10.7554/eLife.103996)

Figure 7-source data

Figure 7A:
V5

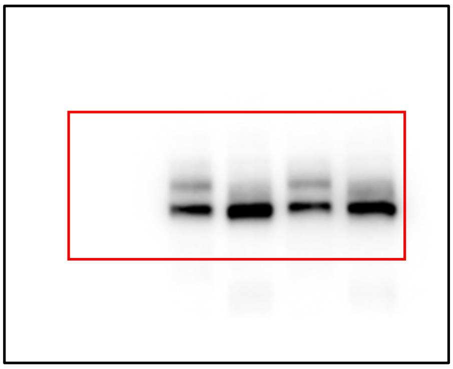

Figure 7A:
HA

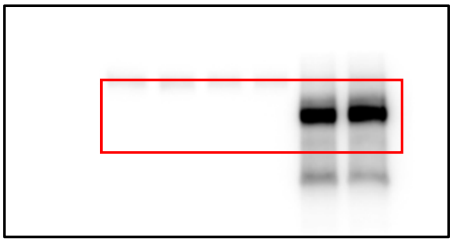

Figure 7A:
StreptAvidin

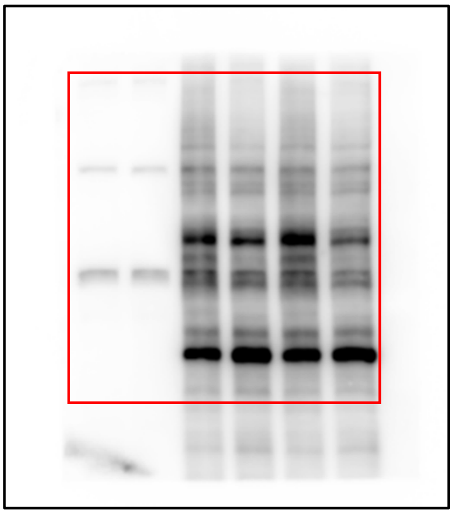

Figure 7A:
Actin

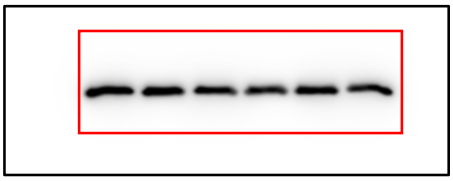

Figure 7B:
NeutrAvidin IP
HA

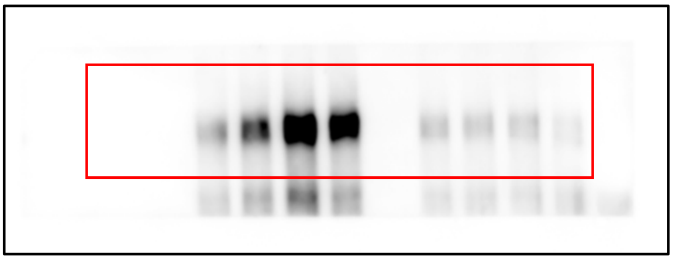

Figure 7B:
Input
HA

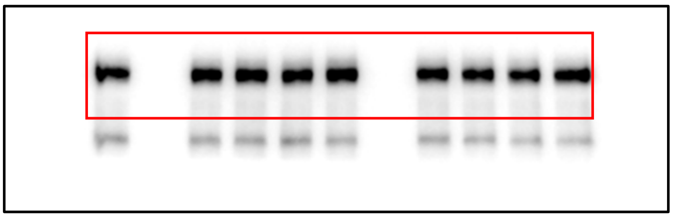

Supplement: Figure 7—source data 2. [file elife-103996-fig7-data2.zip › elife-103996-fig7-data2-v1.pdf]
